# The Association of Prenatal Dietary Factors with Child Autism Diagnosis and Autism-Related Traits Using a Mixtures Approach: Results from the Environmental Influences on Child Health Outcomes Cohort

**DOI:** 10.1016/j.tjnut.2025.02.025

**Published:** 2025-03-17

**Authors:** Megan G Bragg, Juliette Rando, Kecia N Carroll, Stephanie M Eick, Margaret R Karagas, Pi-I Lin, Rebecca J Schmidt, Kristen Lyall, ECHO Cohort Consortium, P Brian Smith, L Kristin Newby, Linda Adair, Lisa P Jacobson, Diane Catellier, Monica McGrath, Christian Douglas, Priya Duggal, Emily Knapp, Amii Kress, Courtney K Blackwell, Maxwell A Mansolf, Jin-Shei Lai, Emily Ho, David Cella, Richard Gershon, Michelle L Macy, Suman R Das, Jane E Freedman, Simon A Mallal, John A McLean, Ravi V Shah, Meghan H Shilts, Akram N Alshawabkeh, Jose F Cordero, John Meeker, Leonardo Trasande, Carlos A Camargo, Zhaozhong Zhu, Ashley F Sullivan, Dana Dabelea, Wei Perng, Traci A Bekelman, Greta Wilkening, Sheryl Magzamen, Brianna F Moore, Anne P Starling, Deborah J Rinehart, Daphne Koinis Mitchell, Viren D'Sa, Sean CL Deoni, Hans-Georg Mueller, Cristiane S Duarte, Catherine Monk, Glorisa Canino, Jonathan Posner, Tenneill Murray, Claudia Lugo-Candelas, Anne L Dunlop, Patricia A Brennan, Christine Hockett, Amy Elliott, Assiamira Ferrara, Lisa A Croen, Monique M Hedderson, John Ainsworth, Leonard B Bacharier, Casper G Bendixsen, James E Gern, Diane R Gold, Tina V Hartert, Daniel J Jackson, Christine C Johnson, Christine LM Joseph, Meyer Kattan, Gurjit K Khurana Hershey, Robert F Lemanske, Susan V Lynch, Rachel L Miller, George T O’Connor, Carole Ober, Dennis Ownby, Katherine Rivera-Spoljaric, Patrick H Ryan, Christine M Seroogy, Anne Marie Singh, Robert A Wood, Edward M Zoratti, Rima Habre, Shohreh Farzan, Frank D Gilliland, Irva Hertz-Picciotto, Deborah H Bennett, Julie B Schweitzer, Rebecca J Schmidt, Janine M LaSalle, Alison E Hipwell, Kate E Keenan, Catherine J Karr, Nicole R Bush, Kaja Z LeWinn, Sheela Sathyanarayana, Qi Zhao, Frances Tylavsky, Kecia N Carroll, Christine T Loftus, Leslie D Leve, Jody M Ganiban, Jenae M Neiderhiser, Scott T Weiss, Augusto A Litonjua, Cindy T McEvoy, Eliot R Spindel, Robert S Tepper, Craig J Newschaffer, Kristen Lyall, Heather E Volk, Rebecca Landa, Sally Ozonoff, Joseph Piven, Heather Hazlett, Juhi Pandey, Robert Schultz, Steven Dager, Kelly Botteron, Daniel Messinger, Wendy Stone, Jennifer Ames, Thomas G O'Connor, Richard K Miller, Emily Oken, Michele R Hacker, Tamarra James-Todd, T Michael O'Shea, Rebecca C Fry, Jean A Frazier, Rachana Singh, Caitlin Rollins, Angela Montgomery, Ruben Vaidya, Robert M Joseph, Lisa K Washburn, Semsa Gogcu, Kelly Bear, Julie V Rollins, Stephen R Hooper, Genevieve Taylor, Wesley Jackson, Amanda Thompson, Julie Daniels, Michelle Hernandez, Kun Lu, Michael Msall, Madeleine Lenski, Rawad Obeid, Steven L Pastyrnak, Elizabeth Jensen, Christina Sakai, Hudson Santos, Jean M Kerver, Nigel Paneth, Charles J Barone, Michael R Elliott, Douglas M Ruden, Chris Fussman, Julie B Herbstman, Amy Margolis, Susan L Schantz, Sarah Dee Geiger, Andrea Aguiar, Karen Tabb, Rita Strakovsky, Tracey Woodruff, Rachel Morello-Frosch, Amy Padula, Joseph B Stanford, Christina A Porucznik, Angelo P Giardino, Rosalind J Wright, Robert O Wright, Brent Collett, Nicole Baumann-Blackmore, Ronald Gangnon, Daniel J Jackson, Chris G McKennan, Jo Wilson, Matt Altman, Judy L Aschner, Annemarie Stroustrup, Stephanie L Merhar, Paul E Moore, Gloria S Pryhuber, Mark Hudak, Ann Marie Reynolds Lyndaker, Andrea L Lampland, Burton Rochelson, Sophia Jan, Matthew J Blitz, Michelle W Katzow, Zenobia Brown, Codruta Chiuzan, Timothy Rafael, Dawnette Lewis, Natalie Meirowitz, Brenda Poindexter, Tebeb Gebretsadik, Sarah Osmundson, Jennifer K Straughen, Amy Eapen, Andrea Cassidy-Bushrow, Ganesa Wegienka, Alex Sitarik, Kim Woodcroft, Audrey Urquhart, Albert Levin, Tisa Johnson-Hooper, Brent Davidson, Tengfei Ma, Emily S Barrett, Martin J Blaser, Maria Gloria Dominguez-Bello, Daniel B Horton, Manuel Jimenez, Todd Rosen, Kristy Palomares, Lyndsay A Avalos, Yeyi Zhu, Kelly J Hunt, Roger B Newman, Michael S Bloom, Mallory H Alkis, James R Roberts, Sunni L Mumford, Heather H Burris, Sara B DeMauro, Lynn M Yee, Aaron Hamvas, Antonia F Olidipo, Andrew S Haddad, Lisa R Eiland, Nicole T Spillane, Kirin N Suri, Stephanie A Fisher, Jeffrey A Goldstein, Leena B Mithal, Raye-Ann O DeRegnier, Nathalie L Maitre, Ruby HN Nguyen, Meghan M JaKa, Abbey C Sidebottom, Michael J Paidas, JoNell E Potter, Natale Ruby, Lunthita Duthely, Arumugam Jayakumar, Karen Young, Isabel Maldonado, Meghan Miller, Jonathan L Slaughter, Sarah A Keim, Courtney D Lynch, Kartik K Venkatesh, Kristina W Whitworth, Elaine Symanski, Thomas F Northrup, Hector Mendez-Figueroa, Ricardo A Mosquera, Margaret R Karagas, Juliette C Madan, Debra M MacKenzie, Johnnye L Lewis, Brandon J Rennie, Bennett L Leventhal, Young Shin Kim, Somer Bishop, Sara S Nozadi, Li Luo, Barry M Lester, Carmen J Marsit, Todd Everson, Cynthia M Loncar, Elisabeth C McGowan, Stephen J Sheinkopf, Brian S Carter, Jennifer Check, Jennifer B Helderman, Charles R Neal, Lynne M Smith

**Affiliations:** 7 Division of Neonatology, Department of Pediatrics, Duke Clinical Research Institute, Duke University School of Medicine, Durham, North Carolina, USA; 8 Division of Cardiology, Department of Medicine, Duke Clinical Research Institute, Duke University School of Medicine, Durham, North Carolina, USA; 9 Department of Nutrition, Gillings School of Global Public Health, University of North Carolina at Chapel Hill, Chapel Hill, North Carolina, USA; 10 Department of Epidemiology, Johns Hopkins University, Bloomberg School of Public Health, Baltimore, Maryland, USA; 11 N/A, Research Triangle Institute, Research Triangle Park, North Carolina, USA; 12 Department of Medical Social Sciences, Feinberg School of Medicine, Northwestern University, Chicago, Illinois, USA; 13 Department of Pediatrics, Feinberg School of Medicine, Northwestern University and Ann & Robert H. Lurie Children's Hospital of Chicago, Chicago, Illinois, USA; 14 Division of Infectious Diseases, Department of Medicine, Vanderbilt University Medical Center, Nashville, Tennessee, USA; 15 Division of Cardiovascular Medicine, Department of Medicine, Vanderbilt University Medical Center, Nashville, Tennessee, USA; 16 Department of Chemistry, Vanderbilt University, Nashville, Tennessee, USA; 17 College of Engineering, Northeastern University, Boston, Massachusetts, USA; 18 College of Public Health, Department of Epidemiology & Biostatistics, University of Georgia, Athens, Georgia, USA; 19 Environmental Health Sciences, School of Public Health, University of Michigan, Ann Arbor, Michigan, USA; 20 Departments of Pediatrics and Population Health, NYU Grossman School of Medicine, New York, New York, USA; 21 Department of Emergency Medicine, Massachusetts General Hospital, Harvard Medical School, Boston, Massachusetts, USA; 22 Lifecourse Epidemiology of Adiposity and Diabetes (LEAD) Center, University of Colorado Anschutz Medical Campus, Aurora, Colorado, USA; 23 Environmental and Radiological Health Sciences, Colorado School of Public Health, Colorado State University, Fort Collins, Colorado, USA; 24 Epidemiology, University of North Carolina at Chapel Hill, Chapel Hill, North Carolina, USA; 25 Center for Health Systems Research, Denver Health and Hospital Authority, Denver, Colorado, USA; 26 Department of Pediatrics, Rhode Island Hospital, The Alpert Medical School of Brown University, Providence, Rhode Island, USA; 27 Division of Gender Equality, Maternal, Newborn & Child Health Discovery & Tools Team, Bill & Melinda Gates Foundation, Seattle, Washington, USA; 28 Department of Statistics, University of California, Davis, Davis, California, USA; 29 Division of Child and Adolescent Psychiatry, Columbia University - NYSPI, New York, New York, USA; 30 Department of Obstetrics & Gynecology, Columbia University - NYSPI, New York, New York, USA; 31 Behavioral Sciences Research Institute, University of Puerto Rico, School of Medicine, Rio Piedras, Puerto Rico; 32 Child & Family Mental Health & Community Psychiatry Division, Duke University School of Medicine, Duke Psychiatry & Behavioral Sciences, Durham, North Carolina, USA; 33 Department of Gynecology and Obstetrics, Emory University School of Medicine, Atlanta, Georgia, USA; 34 Department of Psychology, Emory University, Atlanta, Georgia, USA; 35 N/A, Department of Pediatrics, Avera Research Institute, University of South Dakota School of Medicine, Rapid City, South Dakota, USA; 36 N/A, Department of Pediatrics, Avera Research Institute, University of South Dakota School of Medicine, Sioux Falls, South Dakota, USA; 37 Division of Research, Kaiser Permanente Northern California, Oakland, California, USA; 38 Centre for Health Informatics, University of Manchester, Manchester, United Kingdom; 39 Department of Pediatrics, Monroe Carell Jr Children’s Hospital at Vanderbilt, Vanderbilt University Medical Center, Nashville, Tennessee, USA; 40 National Farm Medicine Center, Marshfield Clinic Research Institute, Marshfield, Wisconsin, USA; 41 Department of Pediatrics, University of Wisconsin School of Medicine and Public Health, Madison, Wisconsin, USA; 42 The Channing Division of Network Medicine, Department of Medicine, Brigham and Women’s Hospital, Harvard Medical School, Boston, Massachusetts, USA; 43 Division of Pediatric Allergy, Immunology, and Pulmonary Medicine, Department of Medicine, Department of Pediatrics, Vanderbilt University Medical Center, Nashville, Tennessee, USA; 44 Department of Public Health Sciences, Henry Ford Health, Detroit, Michigan, USA; 45 Department of Pediatrics, Columbia University Medical Center, New York, New York, USA; 46 Division of Asthma Research, Cincinnati Children’s Hospital Medical Center, Cincinnati, Ohio, USA; 47 Department of Medicine, University of California, San Francisco, California, USA; 48 Department of Medicine, Division of Clinical Immunology, Icahn School of Medicine at Mount Sinai, New York, New York, USA; 49 Department of Pediatrics, Boston University School of Medicine, Boston, Massachusetts, USA; 50 Department of Human Genetics, University of Chicago, Chicago, Illinois, USA; 51 Department of Pediatrics, Washington University School of Medicine, St Louis, Missouri, USA; 52 Department of Pediatrics and College of Medicine, Division of Biostatistics and Epidemiology, University of Cincinnati, Cincinnati, Ohio, USA; 53 Department of Pediatrics, Johns Hopkins University School of Medicine, Baltimore, Maryland, USA; 54 Division of Allergy and Clinical Immunology, Henry Ford Health, Detroit, Michigan, USA; 55 Department of Population and Public Health Sciences, University of Southern California, Los Angeles, California, USA; 56 MIND Institute and Department of Public Health Sciences, University of California, Davis, Davis, California, USA; 57 Department of Public Health Sciences, University of California, Davis, Davis, California, USA; 58 Department of Psychiatry and Behavioral Science and the MIND Institute, University of California, Davis, Davis, California, USA; 59 Psychiatry and Psychology, University of Pittsburgh, Pittsburgh, Pennsylvania, USA; 60 Psychiatry and Behavioral Neuroscience, University of Chicago, Chicago, Illinois, USA; 61 Department of Pediatrics, School of Medicine, Department of Environmental and Occupational Health Sciences, School of Public Health, University of Washington, Seattle, Washington, USA; 62 Department of Psychiatry and Behavioral Sciences and Department of Pediatrics, School of Medicine, University of California, San Francisco, San Francisco, California, USA; 63 Department of Psychiatry and Behavioral Sciences, School of Medicine, University of California, San Francisco, San Francisco, California, USA; 64 Department of Pediatrics, School of Medicine, Department of Environmental and Occupational Health Sciences, School of Public Health, University of Washington and Seattle Children's Research Institute, Seattle, Washington, USA; 65 Department of Preventive Medicine, University of Tennessee Health Science Center, Memphis, Tennessee, USA; 66 Department of Pediatrics, Department of Environmental Medicine & Public Health, Icahn School of Medicine at Mount Sinai, New York, New York, USA; 67 Department of Environmental and Occupational Health Sciences, School of Public Health, University of Washington, Seattle, Washington, USA; 68 Department of Counseling Psychology and Human Services & Prevention Science Institute, University of Oregon, Eugene, Oregon, USA; 69 Department of Psychological and Behavioral Sciences, George Washington University, Washington, DC, USA; 70 Department of Psychology, Penn State University, University Park, Pennsylvania, USA; 71 Channing Division of Network Medicine, Department of Medicine, Brigham and Women's Hospital and Harvard Medical School, Boston, Massachusetts, USA; 72 Pediatric Pulmonary Division, Department of Pediatrics, Golisano Children's Hospital, University of Rochester, Rochester, New York, USA; 73 Division of Neonatology, Department of Pediatrics, Oregon Health & Science University, Portland, Oregon, USA; 74 Division of Neuroscience, Oregon National Primate Research Center, Beaverton, Oregon, USA; 75 Division of Pediatric Pulmonology, Department of Pediatrics, Indiana School of Medicine, Indianapolis, Indiana, USA; 76 College of Health and Human Development, Penn State, State College, Pennsylvania, USA; 77 AJ Drexel Autism Institute, Drexel University, Philadelphia, Pennsylvania, USA; 78 Mental Health, Johns Hopkins University, Baltimore, Maryland, USA; 79 Department of Psychiatry and Behavioral Sciences, Center for Autism and Related Disorders, Kennedy Krieger Institute, Johns Hopkins University, Baltimore, Maryland, USA; 80 MIND Institute, Department of Psychiatry, University of California Davis, Sacramento, California, USA; 81 Department of Psychiatry, University of North Carolina, Chapel Hill, North Carolina, USA; 82 Center for Autism Research, Children's Hospital of Philadelphia, Philadelphia, Pennsylvania, USA; 83 Department of Radiology, University of Washington, Seattle, Washington, USA; 84 Department of Psychiatry, Washington University, St Louis, Missouri, USA; 85 Department of Psychology, University of Miami, Miami, Florida, USA; 86 Department of Psychology, University of Washington, Seattle, Washington, USA; 87 Kaiser Permanente Division of Research, Kaiser Permanente, Oakland, California, USA; 88 Departments of Psychiatry, Neuroscience, Obstetrics and Gynecology, University of Rochester, Rochester, New York, USA; 89 Departments of Obstetrics and Gynecology, University of Rochester, Rochester, New York, USA; 90 Division of Chronic Disease Research Across the Lifecourse, Department of Population Medicine, Harvard Pilgrim Health Care Institute and Harvard Medical School, Boston, Massachusetts, USA; 91 Department of Obstetrics and Gynecology, Beth Israel Deaconess Medical Center, Boston, Massachusetts, USA; 92 Department of Environmental Health, Harvard Chan School of Public Health, Boston, Massachusetts, USA; 93 Division of Neonatology, Department of Pediatrics, University of North Carolina School of Medicine, Chapel Hill, North Carolina, USA; 94 Department of Environmental Sciences and Engineering, University of North Carolina Gillings School of Global Public Health, Chapel Hill, North Carolina, USA; 95 EK Shriver Center and Psychiatry, UMASS Chan Medical School, Worcster, Massachusetts, USA; 96 Department of Pediatrics, Tufts University School of Medicine, Boston, Massachusetts, USA; 97 Department of Neurology, Harvard Medical School, Boston, Massachusetts, USA; 98 Division of Neonatology, Department of Pediatrics, Yale School of Medicine, New Haven, Connecticut, USA; 99 Department of Pediatrics, University of Massachusetts Chan Medical School-Baystate, Springfield, Massachusetts, USA; 100 Department of Anatomy & Neurobiology, Boston University Chobanian & Avedisian School of Medicine, Boston, Massachusetts, USA; 101 Pediatrics, Wake Forest School of Medicine, Winston-Salem, North Carolina, USA; 102 Section of Neonatology, Department of Pediatrics, Wake Forest School of Medicine, Wake Forest University School of Medicine/Atrium Health Wake Forest, Winston-Salem, North Carolina, USA; 103 Section of Neonatology, Department of Pediatrics, ECU Health, Greenville, North Carolina, USA; 104 Department of Health Sciences, School of Medicine, University of North Carolina at Chapel Hill, Chapel Hill, North Carolina, USA; 105 Pediatrics, School of Medicine, University of North Carolina at Chapel Hill, Chapel Hill, North Carolina, USA; 106 Department of Anthropology, Department of Nutrition, University of North Carolina at Chapel Hill, Gillings School of Global Public Health, Chapel Hill, North Carolina, USA; 107 Epidemiology and Maternal and Child Health, University of North Carolina at Chapel Hill, Gillings School of Global Public Health, Chapel Hill, North Carolina, USA; 108 Environmental Sciences and Engineering, Gillings School of Global Public Health, University of North Carolina at Chapel Hill, Chapel Hill, North Carolina, USA; 109 Kennedy Research Center on Intellectual and Neurodevelopmental Disabilities, University of Chicago Medicine: Comer Children's Hospital, Chicago Illinois, USA; 110 Department of Epidemiology and Biostatistics, Michigan State University, East Lansing, Michigan, USA; 111 Pediatrics, Beaumont Hospital, Royal Oak, Michigan, USA; 112 Pediatrics, Corewell Health, Helen DeVos Children's Hospital, Grand Rapids, Michigan, USA; 113 Epidemiology and Prevention, Wake Forest University School of Medicine, Winston-Salem, North Carolina, USA; 114 Pediatrics, Mass General Hospital for Children, Boston, Massachusetts, USA; 115 Dean's Office Graduate School, School of Nursing and Health Studies, University of Miami, Coral Gables, Florida, USA; 116 Departments of Epidemiology & Biostatistics, and Pediatrics & Human Development, Michigan State University, College of Human Medicine, East Lansing, Michigan, USA; 117 Department of Pediatrics, Henry Ford Health, Detroit, Michigan, USA; 118 Department of Biostatistics, University of Michigan, Ann Arbor, Michigan, USA; 119 Department of Obstetrics and Gynecology, Institute of Environmental Health Sciences (IEHS), C.S. Mott Center for Human Health and Development, Wayne State University, Detroit, Michigan, USA; 120 Lifecourse Epidemiology and Genomics Division, Michigan Department of Health and Human Services (MDHHS), Lansing, Michigan, USA; 121 Department of Environmental Health Sciences, Columbia University Mailman School of Public Health, New York, New York, USA; 122 Department of Psychiatry, Columbia University Irving Medical Center, New York, New York, USA; 123 Beckman Institute for Advanced Science and Technology, Department of Comparative Biosciences, University of Illinois Urbana-Champaign, Urbana, Illinois, USA; 124 Beckman Institute for Advanced Science and Technology, Department of Kinesiology and Community Health, University of Illinois Urbana-Champaign, Urbana, Illinois, USA; 125 Beckman Institute for Advanced Science and Technology, Department of Social Work, University of Illinois Urbana-Champaign, Urbana, Illinois, USA; 126 Department of Food Science and Human Nutrition, Michigan State University, East Lansing, Michigan, USA; 127 Program on Reproductive Health and the Environment, University of California, San Francisco, San Francisco, California, USA; 128 Department of Environmental Science, Policy and Management and School of Public Health, University of California, Berkeley, Berkeley, California, USA; 129 Department of Family and Preventive Medicine, Spencer Fox Eccles School of Medicine, University of Utah, Salt Lake City, Utah, USA; 130 Department of Pediatrics, Spencer Fox Eccles School of Medicine, University of Utah, Salt Lake City, Utah, USA; 131 Department of Environmental Medicine & Public Health, Icahn School of Medicine at Mount Sinai, New York, New York, USA; 132 Department of Psychiatry and Behavioral Medicine, University of Washington, Seattle Children's Research Institute, Seattle, Washington, USA; 133 Department of Population Health Sciences, University of Wisconsin, Madison, Wisconsin, USA; 134 Department of Statistics, University of Pittsburgh, Pittsburgh, Pennsylvania, USA; 135 Department of Medicine, University of Washington, Seattle, Washington, USA; 136 Department of Pediatrics, Albert Einstein College of Medicine, Hackensack Meridian School of Medicine, Center for Discovery and Innovation, Bronx, New York, USA, Nutley, New Jersey, USA; 137 Department of Pediatrics, Northwell Health, Cohen Children's Medical Center, and the Zucker School of Medicine at Hofstra / Northwell, New Hyde Park, New York, USA; 138 Department of Pediatrics, Cincinnati Children's, Cincinnati, Ohio, USA; 139 Department of Pediatrics, Vanderbilt University Medical Center, Nashville, Tennessee, USA; 140 Department of Pediatrics, University of Rochester Medical Center, Rochester, New York, USA; 141 Department of Pediatrics, University of Florida College of Medicine, Jacksonville, Florida, USA; 142 Department of Pediatrics, University of Buffalo Jacobs School of Medicine and Biomedical Sciences, Buffalo, New York, USA; 143 Department of Pediatrics, Children's Minnesota, Minneapolis, Minnesota, USA; 144 Department of Obstetrics and Gynecology, Northwell Health and the Zucker School of Medicine at Hofstra / Northwell, New Hyde Park, New York, USA; 145 Department of Science Education, Northwell Health and the Zucker School of Medicine at Hofstra / Northwell, New Hyde Park, New York, USA; 146 Institute of Health System Science, Northwell Health, Feinstein Institutes for Medical Research, Manhasset, New York, USA; 147 Department of Pediatrics, Children's Healthcare of Atlanta Emory University, Atlanta, Georgia, USA; 148 Department of Biostatistics, Vanderbilt University Medical Center, Nashville, Tennessee, USA; 149 Department of Obstetrics and Gynecology, Vanderbilt University Medical Center, Nashville, Tennessee, USA; 150 Department of Women's Health, Henry Ford Health, Detroit, Michigan, USA; 151 Department of Biostatistics and Epidemiology, Environmental and Occupational Health Sciences Institute, Rutgers University, Piscataway, New Jersey, USA; 152 Center for Advanced Biotechnology & Medicine, Rutgers University, Piscataway, New Jersey, USA; 153 Departments of Biochemistry and Microbiology & Anthropology, Rutgers University, New Brunswick, New Jersey, USA; 154 Department of Pediatrics, Robert Wood Johnson Medical School, Rutgers University, New Brunswick, New Jersey, USA; 155 Departments of Pediatrics, Family Medicine, and Community Health, Robert Wood Johnson Medical School, Rutgers University, New Brunswick, New Jersey, USA; 156 Department of Obstetrics, Gynecology, and Reproductive Sciences, Robert Wood Johnson Medical School, Rutgers University, New Brunswick, New Jersey, USA; 157 Department of Obstetrics and Gynecology, Saint Peter's University Hospital, New Brunswick, New Jersey, USA; 158 Department of Public Health Sciences, Medical University of South Carolina, Charleston, South Carolina, USA; 159 Department of Obstetrics and Gynecology, Medical University of South Carolina, Charleston, South Carolina, USA; 160 Department of Global and Community Health, George Mason University, Fairfax, Virginia, USA; 161 Department of Pediatrics, Medical University of South Carolina, Charleston, South Carolina, USA; 162 Department of Biostatistics, Epidemiology and Informatics, Department of Obstetrics and Gynecology, University of Pennsylvania Perelman School of Medicine, Philadelphia, Pennsylvania, USA; 163 Division of Neonatology, Department of Pediatrics, Children's Hospital of Philadelphia, University of Pennsylvania Perelman School of Medicine, Philadelphia, Pennsylvania, USA; 164 Division of Maternal-Fetal Medicine, Department of Obstetrics & Gynecology, Feinberg School of Medicine, Northwestern University, Chicago, Illinois, USA; 165 Division of Neonatology, Department of Pediatrics, Ann & Robert H. Lurie Children's Hospital, Feinberg School of Medicine, Northwestern University, Chicago, Illinois, USA; 166 Division of Maternal-Fetal Medicine, Department of Obstetrics & Gynecology, Hackensack University Medical Center, Hackensack Meridian School of Medicine, Nutley, New Jersey, USA; 167 Division of Neonatology, Department of Pediatrics, Hackensack University Medical Center, Hackensack Meridian School of Medicine, Nutley, New Jersey, USA; 168 Division of Developmental and Behavioral Pediatrics, Department of Pediatrics, Hackensack University Medical Center, Hackensack Meridian School of Medicine, Nutley, New Jersey, USA; 169 Department of Pathology, Feinberg School of Medicine, Northwestern University, Chicago, Illinois, USA; 170 Division of Infectious Diseases, Department of Pediatrics, Ann & Robert H. Lurie Children's Hospital, Feinberg School of Medicine, Northwestern University, Chicago, Illinois, USA; 171 Division of Neonatology, Department of Pediatrics, Emory University School of Medicine and Cerebral Palsy Foundation, Atlanta, Georgia, USA New York, New York, USA; 172 Division of Epidemiology & Community Health, School of Public Health, University of Minnesota, Minneapolis, Minnesota, USA; 173 Division of Research & Evaluation, HealthPartners Institute, Minneapolis, Minnesota, USA; 174 Care Delivery Research, Allina Health, Minneapolis, Minnesota, USA; 175 Department of Obstetrics and Gynecology, University of Miami Miller School of Medicine, Miami, Florida, USA; 176 Department of Obstetrics, Gynecology and Reproductive Sciences, University of Miami Miller School of Medicine, Miami, Florida, USA; 177 Mailman Center for Child Development, University of Miami Miller School of Medicine, Miami, Florida, USA; 178 Department of Obstetrics, Gynecology and Reproductive Sciences and Department of Public Health Sciences, University of Miami School of Medicine, Miami, Florida, USA; 179 Department of Pediatrics, University of Miami Miller School of Medicine, Miami, Florida, USA; 180 School of Nursing and Health Studies, University of Miami, Miami, Florida, USA; 181 Psychiatry and Behavioral Sciences, MIND Institute, University of California Davis, Sacramento, California, USA; 182 Center for Perinatal Research, Abigail Wexner Research Institute and Division of Neonatology, Nationwide Children's Hospital and Department of Pediatrics, College of Medicine and Division of Epidemiology, College of Public Health, The Ohio State University, Nationwide Children's Hospital and The Ohio State University, Columbus, Ohio, USA; 183 Center for Biobehavioral Health, Abigail Wexner Research Institute, Nationwide Children's Hospital and Department of Pediatrics, College of Medicine and Division of Epidemiology, College of Public Health, The Ohio State University, Nationwide Children's Hospital and The Ohio State University, Columbus, Ohio, USA; 184 Division of Maternal-Fetal Medicine, Department of Obstetrics and Gynecology, College of Medicine and Division of Epidemiology, College of Public Health, The Ohio State University, Columbus, Ohio, USA; 185 Center for Precision Environmental Health and Department of Medicine, Baylor College of Medicine, Houston, Texas, USA; 186 Department of Family and Community Medicine, University of Texas Health Science Center at Houston (UTHealth Houston) McGovern Medical School, Houston, Texas, USA; 187 Department of Obstetrics, Gynecology and Reproductive Sciences, University of Texas Health Science Center at Houston (UTHealth Houston) McGovern Medical School, Houston, Texas, USA; 188 Department of Pediatrics, University of Texas Health Science Center at Houston (UTHealth Houston) McGovern Medical School, Houston, Texas, USA; 189 Department of Epidemiology, Geisel School of Medicine at Dartmouth, Hanover, New Hampshire, USA; 190 Departments of Psychiatry, Pediatrics & Epidemiology, Geisel School of Medicine at Dartmouth, Dartmouth Hitchcock Medical Center, Hanover, New Hampshire, USA; 191 Community Environmental Health Program, Department of Pharmaceutical Sciences, College of Pharmacy, University of New Mexico Health Sciences Center, Albuquerque, New Mexico, USA; 192 Center for Development and Disability, University of New Mexico, Albuquerque, New Mexico, USA; 193 Community Environmental Health Program, Department of Pharmaceutical Sciences UNM, College of Pharmacy, University of New Mexico Health Sciences Center, University of Chicago, Albuquerque, New Mexico, USA, Chicago, Illinois, USA; 194 Department of Psychiatry and Behavioral Sciences, University of California, San Francisco, San Francisco, California, USA; 195 Department of Internal Medicine, Comprehensive Cancer Center, University of New Mexico Health Sciences Center, Albuquerque, New Mexico, USA; 196 Department of Pediatrics, Department of Psychiatry and Human Behavior, Warren Alpert Medical School of Brown University, Providence, Rhode Island, USA; 197 Department of Environmental Health, Rollins School of Public Health, Emory University, Atlanta, Georgia, USA; 198 Department of Psychiatry and Human Behavior, Warren Alpert Medical School of Brown University, Providence, Rhode Island, USA; 199 Department of Pediatrics, Warren Alpert Medical School of Brown University, Providence, Rhode Island, USA; 200 Department of Pediatrics, Thompson Center for Autism & Neurodevelopment, University of Missouri, Columbia, Missouri, USA; 201 Department of Pediatrics, Children's Mercy-Kansas City, Kansas City, Missouri, USA; 202 Department of Pediatrics, Wake Forest School of Medicine, Winston-Salem North Carolina, USA; 203 Department of Pediatrics, University of Hawaii John A Burns School of Medicine, Honolulu, Hawaii, USA; 204 Department of Pediatrics, UCLA Clinical and Translational Science Institute at The Lundquist Institute, Harbor-UCLA Medical Center, Los Angeles, California, USA; 1 AJ Drexel Autism Institute, Drexel University, Philadelphia, PA, United States; 2 The Icahn School of Medicine at Mount Sinai, New York, NY, United States; 3 Rollins School of Public Health, Emory University, Atlanta, GA, United States; 4 Geisel School of Medicine, Dartmouth College, Hanover, NH, United States; 5 Department of Population Medicine, Harvard Medical School and Harvard Pilgrim Health Care Institute, Boston, MA, United States; 6 Department of Public Health Sciences, School of Medicine, University of California Davis, Davis, CA, United States

**Keywords:** neurodevelopment, nutrients, statistical mixtures methods, epidemiology, autism

## Abstract

**Background:**

Previous research on the role of maternal diet in relation to autism has focused on examining individual nutrient associations. Few studies have examined associations with multiple nutrients using mixtures approaches, which may better reflect true exposure scenarios.

**Objectives:**

This study aims to examine associations of nutrient mixtures with children’s autism diagnosis and trait scores within a large, diverse population.

**Methods:**

Participants were drawn from the United States Environmental influences on Child Health Outcomes (ECHO) consortium. Maternal prenatal diet was reported via validated food frequency questionnaires. Children’s autism-related traits were measured using the Social Responsiveness Scale (SRS) and autism diagnoses were from parent reports of physician diagnosis. Bayesian kernel machine regression was used to examine the overall mixture effect and interactions between a set of 5 primary nutrients (folate, vitamin D, omega 3 and omega 6 fatty acids, and iron), adjusted for potential confounders, in relationship to child outcomes. Secondary analyses were conducted in a subset of cohorts with an expanded set of 14 nutrients. Traditional linear and logistic regression models were also analyzed for comparison of results to mixture models.

**Results:**

A total of 2614 participants drawn from 7 ECHO cohorts were included in primary analysis. Mixture analyses suggested that increasing the overall 5-nutrient mixture was associated with lower SRS scores. Individual U-shaped associations and bivariate interactions between folate and omega 3 fatty acids were suggested. In the subset included in the secondary analyses of the 14-nutrient mixture, a modest inverse trend remained, but individual nutrient associations were altered, with vitamin D demonstrating higher relative importance than other nutrients. Strong associations with autism diagnosis were not observed.

**Conclusions:**

In this large sample, we found evidence for combined nutrient effects with broader autism-related traits. Because results for individual nutrients were sensitive to mixture components, replication of combined associations between nutrients and autism-related outcomes is needed.

## Introduction

Prenatal nutrition is critical for children’s neurodevelopment. Nutrients such as iron, fatty acids, and folic acid have been associated with neurodevelopmental outcomes [1] and could potentially influence the development of autism, but the evidence is inconclusive [2]. Autism is a neurodevelopmental condition that significantly affects communication and social interaction. In the United States, the prevalence of autism has increased over time, with the most recent estimate suggesting 1 in 36 children have an autism diagnosis by age 8 [3]. The etiology of autism is thought to be multifactorial, including both genetic and environmental contributions [4,5], with the perinatal period potentially a crucial window for autism development [6,7]. Additionally, evidence suggests traits related to autism follow a continuous distribution in the general population and may be useful to consider for etiologic investigations [8,9].

Higher prenatal intakes of folic acid, polyunsaturated fatty acids (PUFAs), and vitamin D have each been associated with lower odds of autism diagnosis and autism traits scores [2]. These nutrients are thought to influence neurodevelopment via effects on gene expression, oxidation, and inflammation, among other pathways [10,11]. For example, folic acid intake during early pregnancy has been associated with lower odds of autism diagnosis in several studies [12,13]. Folic acid influences 1-carbon metabolism and gene methylation, a pathway implicated in autism development, given evidence for altered methylation patterns among autistic individuals [[12], [13], [14], [15]]. PUFAs and vitamin D have also been associated with autism and general neurodevelopment [[16], [17], [18], [19], [20]]. These nutrients influence multiple pathways, including those related to inflammation and oxidation, which have in turn been associated with autism development [21]. Other nutrients that influence these pathways (such as choline and betaine, which influence 1-carbon metabolism) may also have the potential to influence autism outcomes but have been less studied to date.

Although previous work has focused on the role of individual nutrients (that is, folic acid) in relationship to autism, nutrients occur together within foods and dietary patterns, and intakes are highly correlated. Nutrients may also interact with each other biologically, underscoring the importance of considering their coexposures. Traditional statistical models may not adequately capture these biological interactions and correlations. Moreover, statistical methods designed to assess mixtures are particularly useful for examining multiple, interrelated exposures. One such method is Bayesian kernel machine regression (BKMR), which uses a kernel function to flexibly estimate exposure–response associations for a mixture of potentially correlated exposures with adjustment for covariates [22]. One strength of BKMR is that it can accommodate nonlinear, nonadditive relationships, and the exposure–response relationship does not have to be in the same direction (that is, positive or negative) for all exposures in the mixture. Additionally, BKMR allows for hierarchical grouping of exposures based on correlation or hypothesized relationship. Full descriptions of BKMR are available elsewhere [22,23]. Similar mixtures methods have been used to examine associations of environmental chemical mixtures with neurodevelopmental outcomes [24,25], but the application of these methods for diet-related analyses is far less common. In prior work, members of our team used BKMR to examine the association of prenatal intake of 10 nutrients with autism outcomes in 2 prospective cohorts: the Early Autism Risk Longitudinal Investigation (EARLI) study, a familial autism cohort study following younger siblings of autistic children, and the Nurses Health Study II (NHSII), a cohort of registered United States nurses drawn from the general population [26]. Although we did not find strong individual or combined associations of nutrients with autism traits scores in the EARLI familial autism cohort, there were moderate inverse associations of the overall mixture and of individual nutrients (omega 6 fatty acids, iron, vitamin B_12_) with Social Responsiveness Scale (SRS) score and moderate positive associations with omega 3 fatty acids in the NHSII cohort. However, this prior analysis included relatively small samples (*n* = 127 in EARLI and 713 in NHSII) with limited racial/ethnic diversity, potentially limiting the ability to detect associations.

Therefore, to address the role of combined effects of a suite of nutrients and clarify associations with autism-related outcomes and specific key nutrients in a large diverse sample, we employed mixture methods using data from the United States Environmental influences on Child Health Outcomes (ECHO) cohort consortium [27]. We hypothesized, given prior signals with individual nutrients, that overall intake of a mixture of neurodevelopmentally relevant nutrients would be inversely associated with autism related-outcomes, and that novel interactions between nutrients may be identified using mixture modeling approaches.

## Methods

### Participants

The ECHO Program includes over 38,000 pregnancies and 60,000 children with an overall goal of understanding the role of early-life exposures on children’s health, including neurodevelopment [27]. As of 2023, 69 prenatal and pediatric cohort studies across the United States had joined the ECHO Program, representing births spanning from the 1980s to the present [28], contributing previously collected data and agreeing to further data collection under a common ECHO Protocol. The ECHO Cohort includes pregnancies at enriched risk of autism along with those drawn from the general population [28].

All dyads with available maternal prenatal food frequency data and child autism outcomes data as of 1 March, 2023 were included in the current study, as long as *1*) both mother and child had consented to participation in ECHO, and *2*) children were the result of a singleton pregnancy. For mothers with multiple eligible children, we randomly selected 1 child. Descriptions of the 7 ECHO cohort study sites (corresponding to 2614 unique mother–child dyads) that contributed data for this analysis are provided in Supplemental Table 1.

### Prenatal nutrient intake measures

ECHO cohort study sites have collected several types of dietary data, including 24-h recalls and food frequency questionnaires (FFQs) [29]. Differences in nutrient intake by the assessment method have been documented in ECHO [30] and elsewhere [31,32]. For this analysis, only prenatal FFQ data were included, as FFQs better capture usual daily intake and dietary patterns [33].

Three different FFQs were collected by the cohorts included in this analysis: the Block FFQ [34], the Harvard Willett FFQ [35], and the Diet History Questionnaire (DHQ) [36]. Each of these validated [37] questionnaires asks respondents to describe how often they consumed a list of >100 foods and beverages over a specified time frame. Participants in a subset of cohorts were also asked about supplement use. All questionnaires were completed by participants during pregnancy. If mothers completed >1 FFQ during pregnancy, the first FFQ was chosen to align with the early pregnancy period, a timepoint in which nutritional exposures have been linked to autism outcomes in previous studies [2].

Cohorts used standardized nutrient databases appropriate for their FFQ to convert raw food frequency data into usual nutrient intake and then shared the data with ECHO analysts, who harmonized nutrients to standard units. Because of differences in the underlying nutrient databases and original cohort study aims, the nutrients available for each cohort varied slightly. All cohorts provided data on dietary intake of our 5 priority nutrients: folate, vitamin D, omega 3 fatty acids, omega 6 fatty acids, and iron. These nutrients were a priori selected for analyses based on their associations with autism outcomes in previous literature [2]. Of the 7 cohorts, 4 also provided data on 9 additional nutrients: vitamin B_12_, vitamin B_6_, choline, betaine, methionine, zinc, vitamin E (alpha-tocopherol), vitamin A, and vitamin C. These nutrients are involved in related biological pathways (including antioxidant, inflammatory, and epigenetic) that may influence neurodevelopmental outcomes but have been less studied in prior literature. Because dietary supplement use was not consistently captured across cohorts, only intake from dietary sources is included in the primary analysis.

### Children’s autism outcomes

Two autism outcomes were considered in this analysis: autism diagnosis (yes/no) and scores from the SRS-2, a quantitative measure of autism-related traits [38,39]. Parents were asked whether a doctor or other health care provider ever told them that their child had autism, autism spectrum disorder (ASD), Asperger’s disorder, or pervasive developmental disorder. In some cohorts, diagnoses were confirmed according to clinical assessments or medical record abstraction; however, parent-reported diagnosis was used here because it was harmonized across cohorts. The SRS is a validated and widely used informant-report questionnaire that quantitatively measures children’s autism-related traits, including social communication, social cognition, restricted repetitive behaviors, autistic mannerisms, attention, and social motivation [40]. The SRS has well-established psychometric properties, with high internal validity, reliability, and reproducibility [40] and can be applied to both clinical populations and the general population [8]. Higher scores from general population samples have also been shown to correlate well with clinical autism diagnosis [40]. There are several forms of the SRS based on age at administration and length: a preschool form (for children aged 2.5–4.5 y), a school-age form (4–18 y), full-length forms (65 items), and short forms (16 items). All versions of the SRS have been validated and yield comparable total raw scores, with higher scores reflecting greater deficits in social communication. Short-form scores are scaled to yield the same total score range as full forms. We included all versions of the SRS in this analysis, as they have been found to have strong agreement in their distributional properties, risk factor associations, predictions of autism diagnosis, and familiality, including in the ECHO population [[41], [42], [43], [44], [45]]. T-scores can also be calculated; in this case, raw scores were chosen for primary analysis, rather than T-scores, as recommended by the publisher for use in studies of population-level effects. Forms were excluded if there was >15% missingness or the age of administration was <2.5 or >18 y.

Although all cohorts included in this analysis collected information on both autism diagnosis and SRS scores, the number of individuals with data varied across autism outcomes (that is, some participants only had information on autism diagnosis, whereas some only had SRS scores). Because our previous work with BKMR has focused on continuous outcomes [26] and because the evidence supports the use of quantitative traits scores in autism research [9], SRS was the primary, continuous outcome examined in this analysis. Autism diagnosis was considered a secondary, binary outcome given the modest number of individuals with an autism diagnosis (*n* = 137) in our analytic sample.

### Statistical analysis

Descriptive statistics were calculated for nutrient intakes, autism outcomes, and covariates of interest. Dietary nutrient intakes were compared with United States recommended dietary allowances or adequate intake levels for pregnancy where available. United States recommended intakes during pregnancy were not available for total omega 3 or total omega 6 fatty acids, betaine, or methionine. Because some of the dietary intake variables had skewed distributions, Spearman’s correlations were calculated to examine monotonic relationships among nutrients.

### Primary analysis: BKMR analysis of 5 nutrients with SRS score

We used BKMR to examine the combined, individual, and joint associations of maternal dietary nutrient intakes with SRS total raw scores, adjusting for potential confounders.

We used the R package *bkmr* (version 0.2.2) for analysis. First, we ran the Markov chain Monte Carlo (MCMC) sampler, which randomly samples from the distribution to fit the model. To improve computing performance while maximizing the number of iterations, parallel chains were utilized, with 5 chains and 5000 iterations per chain. Default prior parameters were used. The R package *bkmrhat* (version 1.1.3) was used to assess model fit. Prior work has shown that BKMR is able to detect modest associations in sample sizes as small as 100, suggesting our sample size was more than adequate for estimating associations under this approach [22].

BKMR models were used to estimate exposure–outcome relationships in different ways, each illustrated in a separate figure. First, we estimated the overall mixture effect, or the combined association of the nutrient mixture with SRS scores. Second, we estimated individual nutrient associations with SRS scores while holding the remaining nutrients constant at their median value. Third, we examined how associations of single nutrients with SRS scores changed when the remaining nutrients were set to their 25th, 50th, or 75th percentiles. Finally, we used BKMR to estimate bivariate exposure–response functions, which show the association of a single nutrient with SRS at varying levels of a second nutrient.

In addition, BKMR produces posterior inclusion probabilities (PIPs) for the inclusion of a nutrient in the MCMC sampler, representing the relative importance of each exposure in the model. PIPs range from 0 to 1, with higher values representing higher importance. In the same way, group PIPs describe the relative importance of a group of nutrients. Conditional PIPs describe the relative strength of each nutrient within a group, as given by the percentage of iterations in which the MCMC sampler identifies each nutrient as the most important when the group is included in the model [22].

For the primary model, we included 5 nutrients in our exposure mixture: folate, vitamin D, omega 3 fatty acids, omega 6 fatty acids, and iron. Before inclusion in the models, all nutrients were energy-adjusted and standardized using a *z*-score. In addition to treating each nutrient independently within the model, we used a hierarchical grouping of nutrients, with vitamin D, iron, and omega 3, omega 6 fatty acids representing an “inflammatory pathways” group, and folate in a separate “1-carbon metabolism” group.

Models were adjusted by covariates with previously reported associations with autism outcomes or maternal prenatal diet: maternal age (continuous), prepregnancy BMI (continuous), cohort type (familial autism cohort or general population), maternal race and ethnicity (non-Hispanic White, non-Hispanic Black, Hispanic, and other), maternal education (less than high school, high school degree/General Educational Development test (GED), some college/associate’s/trade school, bachelor’s degree, master’s/professional/doctorate degree), maternal smoking (yes/no), child sex (male, female), and child year of birth (1998–2004, 2005–2009, 2010–2014, 2015+). We include race and ethnicity as a social construct which may serve as a proxy for structural inequities associated with maternal diet and children’s autism-related outcomes [46]. Missingness was low (0%–3%) for variables included in primary analyses. Complete information on missingness is included in Table 1. Missing values were filled using single imputation estimated from linear regression using values from all other covariates.

**TABLE 1:** Characteristics of ECHO participants included in primary analysis of nutrient intake with SRS scores (*n* = 2614).

Characteristic	*n* (%)
Cohort type	
Autism (familial) enriched risk	222 (8.49)
Nonenriched/population-based	2392 (91.51)
Maternal race/ethnicity	
Non-Hispanic White	1719 (65.76)
Non-Hispanic Black	536 (20.50)
Hispanic	178 (6.81)
Other	176 (6.73)
Missing	5 (0.19)
Maternal smoking	
Yes	145 (5.55)
No	2448 (93.65)
Missing	21 (0.8)
Prenatal supplement use (ever)	
Yes	2102 (80.41)
No	134 (5.13)
Missing	378 (14.46)
Maternal education	
<High school	102 (3.9)
High school degree, GED	427 (16.34)
Some college/associate’s/trade school	653 (24.98)
Bachelor’s degree	648 (24.79)
Master’s, professional or doctorate degree	704 (26.93)
Missing	80 (3.06)
Household income	
<$30,000	225 (8.61)
$30,000–$49,999	142 (5.43)
$50,000–$74,999	188 (7.19)
$75,000–$99,999	214 (8.19)
$100,000 or more	767 (29.34)
Missing	1078 (41.24)
Child sex	
Male	1313 (50.23)
Female	1301 (49.77)
Preterm birth	
Term (≥37 wk gestation)	2451 (93.76)
Preterm (<37 gestation)	<165^1^
Missing	<5^1^
Child birth year	
1998–2004	574 (21.96)
2005–2009	453 (17.333)
2010–2014	1261 (48.24)
2015+	326 (12.47)
Breastfed (ever)	
Yes	1546 (59.14)
No	24 (0.92)
Missing	1044 (39.94)
Child autism diagnosis	
Autism	107 (4.09)
No autism	2285 (87.41)
Missing	222 (8.49)
SRS-2 instrument	
16 items	380 (14.54)
65 items	2234 (85.46)
SRS-2 form type	
School age	2123 (81.22)
Preschool	491 (18.78)
Diet form type	
Block	952 (36.42)
DHQ2	91 (3.48)
Harvard	1571 (60.10)

	Mean (SD)

Maternal age	31.30 (5.42)
Birthweight	3393.05 (541.70)
Prepregnancy BMI	26.08 (6.37)
Parity	0.95 (1.03)
SRS total raw score	25.17 (20.62)
SRS total T score	46.60 (8.03)

Abbreviations: DHQ, Diet History Questionnaire; ECHO, Environmental influences on Child Health Outcomes; GED, General Educational Development Test; SRS, Social Responsiveness Scale.

1 To preserve confidentiality, we have suppressed exact values in bins with small *n*’s (<5), along with the value for ≥1 other bin in that variable.

### Secondary analyses

We performed 3 secondary analyses. First, we included an expanded suite of 14 nutrients as our exposure mixture in the BKMR model. The additional nutrients were as follows: vitamin B_12_, vitamin B_6_, choline, betaine, and methionine (included with folate in the “1-carbon metabolism” group); vitamin E and zinc (included in the “inflammatory pathways” group); and vitamins A and C, which were grouped together as the “antioxidants” group. These analyses were secondary because only a subset of participants had complete data on all 14 nutrients. Second, we used BKMR models with a probit link function to examine the association of nutrient intakes with dichotomous autism diagnosis, using the same methods as described previously, in the sample with diagnostic data. Third, to compare with previous work assessing individual nutrients, we also conducted analyses using traditional regression models. Nutrients were individually examined in separate quantile regression (for SRS, modeled at the 50th percentile) and logistic regression (for autism diagnosis) models. Bivariate interactions suggested in BKMR analysis were then also explored in standard adjusted regression models using interaction terms to obtain a *P* value for the interaction.

### Sensitivity analyses

To test the robustness of results in our primary analysis, we performed several sensitivity analyses. First, BKMR analysis of the 5 primary nutrients was examined in the analytic subsample of participants who had data for all 14 nutrients to assess differences between the subsample and the full study group. Second, BKMR analysis was performed for total intake from diet and supplements of the 5 primary nutrients in the analytic subsample with data on supplement intake. Although supplemental intake of omega 6 fatty acids was not measured in any cohort, dietary intake of this nutrient was included in the model because we considered omega 6 intake from supplements to be uncommon [47]. Because Bayesian kernal machine regression (BKMR) is computationally intensive, the remaining sensitivity analyses were conducted in traditional regression models; if differences from the primary analysis were identified, the sensitivity analysis was also performed using BKMR models. To assess the role of potential cohort-specific effects, we conducted covariate-adjusted quantile regression analyses of nutrient-SRS associations leaving out individual cohorts 1 at a time. Additionally, we tested primary models for further adjustment of several additional variables of interest, which were not included in primary models because of high missingness or limited variability within the sample, including prenatal vitamin use (ever during pregnancy), pregnancy complications, preterm birth, breastfeeding (ever), parity, and income. For variables with <25% missingness, imputation was used to assign missing values as in the primary analysis, whereas for variables with higher missingness (25%–50%), a “missing” category was created. Finally, we included adjustment for FFQ form type (Block compared with Willett compared with DHQ), which was not included in primary models because of the high correlation with cohort type. Because the addition of this variable altered regression results, we performed regression models stratified by FFQ type and, separately, tested FFQ type as a covariate in primary BKMR models.

## Results

### Participant characteristics

A total of 2614 maternal-child dyads from 7 ECHO cohort study sites were included in our primary analysis of nutrient intake and SRS scores (Supplemental Figure 1). Participants in this analysis were broadly comparable to the general ECHO population [28], with most maternal participants being non-Hispanic White, nonsmokers, with more than a high school education, and enrolled in cohorts that sampled from the general population (Table 1). Most children were born at term (≥37 wk gestational age). The mean (SD) SRS total raw score was 25.2 (20.6). The majority of dietary data included in our primary sample was collected using the Harvard/Willett FFQ (60%), with most of the remaining data collected via the Block FFQ. Average intakes of folate, zinc, and vitamins B_6_, B_12_, A, and C approached or exceeded United States recommended intakes for pregnancy, whereas average intakes of vitamins D, E, iron, and choline were below recommendations (Supplemental Table 2). A Spearman correlation matrix revealed associations between several nutrient pairs, including a strong positive correlation between omega 3 and omega 6 fatty acids for both the 5-nutrient and 14-nutrient samples (0.78), as well as iron and folate (0.82) and vitamin B_12_ and zinc (0.72) for the 14-nutrient sample (Supplemental Figure 2). A comparison of nutrient intake values by cohort is shown in Supplemental Table 3.

### Primary analysis: BKMR analysis of 5 nutrients with SRS score

**FIGURE 1:**
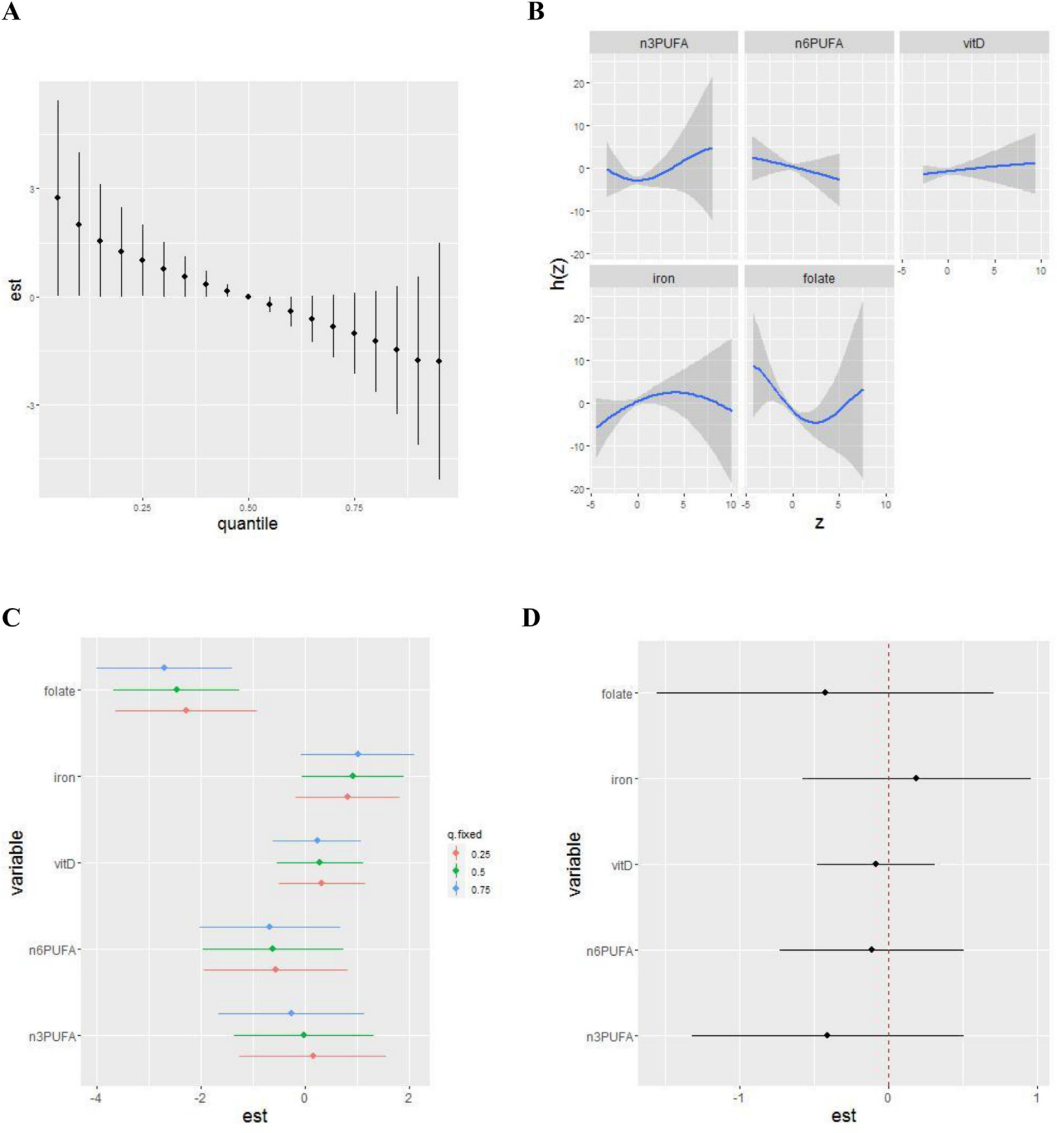
Adjusted associations between prenatal intake of 5 nutrients and child SRS scores using hierarchical BKMR (*n* = 2614). Results of Bayesian kernel machine regression (BKMR) analyses in ECHO including energy-adjusted nutrient intake for omega 3 and omega 6 fatty acids, iron, vitamin D, and folate, adjusted for maternal age, maternal prepregnancy BMI, child sex (male and female), cohort type (enriched familial autism probability and general population), maternal ethnicity/race (non-Hispanic White, non-Hispanic Black, Hispanic, and other), maternal education (less than high school, high school/GED, some college/associates degree/trade school, bachelor’s degree, and graduate degree), maternal smoking (yes and no), and child year of birth (1998–2004, 2005–2009, 2010–2014, and 2015+). Plots show: (A) the overall mixture effect, or the relationship between the nutrient mixture and child SRS scores. (B) Individual nutrient associations with SRS scores, holding all other nutrients at their 50th percentile. (C) Single-exposure effects; plot shows the impact on a child SRS score when each exposure increases from its 25th to 75th percentile (whereas other exposures are fixed at their 25th, 50th, or 75th percentiles). (D) Interaction parameters; plot compares the single-exposure health risk of each exposure (associated with a change from its 25th to 75th percentile) when other exposures are fixed to their 75th percentile compared with their 25th percentile. The primary set of nutrients included here was selected as described in the text based on a priori interest for neurodevelopmental relevance and grouped by shared pathway. ECHO, Environmental influences on Child Health Outcomes; SRS, Social Responsiveness Scale.

We ran BKMR models to estimate combined, individual, and bivariate associations of a suite of nutrients with autism outcomes. Convergence criteria were met for all models. Because results were similar with and without hierarchical grouping of exposures, we have only presented results from hierarchical models here. In covariate-adjusted models, we observed an inverse association between the overall 5-nutrient mixture and SRS scores. A dose–response trend across the mixture was suggested, with increases of ∼3 points in SRS scores for the lowest quantile of nutrient intake (with credible intervals narrowly excluding the null) to reductions of ∼1.5–2 points at higher levels of the mixture, compared with median intakes (Figure 1A). Plots of the associations of individual nutrients with SRS revealed U-shaped relationships with total omega 3 fatty acids and folate, with credible intervals excluding the null at certain points in the distribution (Figure 1B). Inverse associations of folate and omega 3 fatty acids with SRS were somewhat stronger when other nutrients were fixed at the 75th quantile (compared with 50th or 25th) (Figure 1C), but credible intervals for these differences included the null (Figure 1D).

**FIGURE 2:**
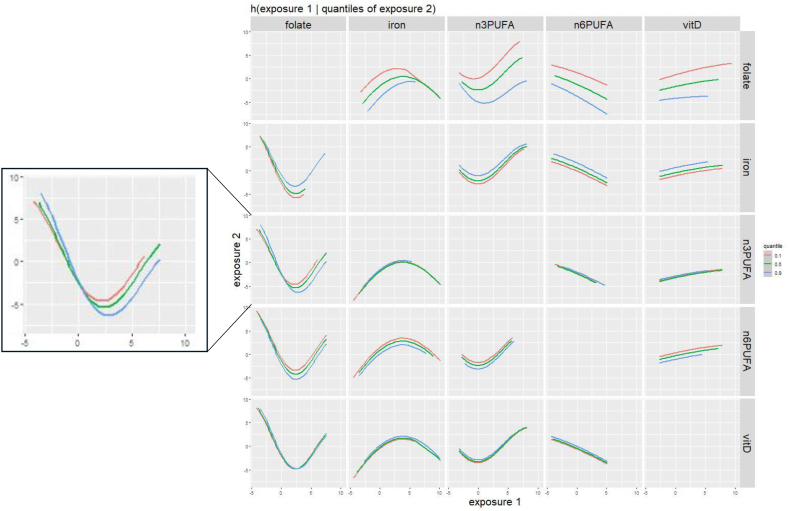
Adjusted bivariate associations between prenatal intake of 5 nutrients and child SRS scores using hierarchical Bayesian kernel machine regression (BKMR) (*n* = 2614). Results of BKMR analyses in ECHO including energy-adjusted nutrient intake for omega 3 and omega 6 fatty acids, iron, vitamin D, and folate, adjusted for maternal age, maternal prepregnancy BMI, child sex (male and female), cohort type (enriched familial autism probability and general population), maternal ethnicity/race (non-Hispanic White, non-Hispanic Black, Hispanic, and other), maternal education (less than high school, high school/GED, some college/associates degree/trade school, bachelor’s degree, and graduate degree), maternal smoking (yes and no), and child year of birth (1998–2004, 2005–2009, 2010–2014, and 2015+). Plots show bivariate exposure–response function for 2 predictors, where the second predictor is fixed at various percentiles (10th, 50th, and 90th), and all other predictors are fixed at the 50th percentile. The magnified plot shows the association of folate with SRS score at different quantiles of omega 3 fatty acids. Intersecting lines suggest an interaction between folate and omega 3 fatty acids. ECHO, Environmental influences on Child Health Outcomes; SRS, Social Responsiveness Scale.

When assessing bivariate nutrient interactions, there was suggestive evidence of a complex interaction between folate and omega 3 fatty acids, as evidenced by intersecting slopes in Figure 2. At lower folate intakes, there was a slightly stronger inverse association with SRS scores if omega 3 fatty acid intake was in the highest quantile, compared with lower quantiles. However, at higher folate intakes, there were stronger positive associations with SRS scores if omega 3 fatty acid intake was in the highest quantile. This suggests that omega 3 fatty acid intake may enhance associations of folate with SRS score (both in a positive and negative direction).

When examining the relative contribution of each nutrient and group of nutrients, results suggested folate played the strongest role in relationships with SRS scores, as evidenced by a higher group PIP compared with the “inflammatory pathways” group (Table 2) [22]. Within the “inflammatory pathway” group, omega 3 fatty acids appeared to play the strongest role, with the highest conditional PIP.

**TABLE 2:** Posterior inclusion probabilities (PIPs) from adjusted, hierarchical BKMR with child SRS scores in 5-nutrient and 14-nutrient analyses^1^.

	5 nutrient analysis (*n* = 2614)			14 nutrient analysis (*n* = 952)		
Nutrient	Group	Group PIP	Conditional PIP	Group	Group PIP	Conditional PIP
Folate	1-C metab	0.86	1	1-C metab	0.21	0.06
Omega 3 fatty acids	Inflammatory	0.47	0.51	Inflammatory	0.54	0.04
Iron	Inflammatory	0.47	0.37	Inflammatory	0.54	0.04
Omega 6 fatty acids	Inflammatory	0.47	0.09	Inflammatory	0.54	0.01
Vitamin D	Inflammatory	0.47	0.03	Inflammatory	0.54	0.84
Zinc				Inflammatory	0.54	0.04
Vitamin E				Inflammatory	0.54	0.02
Vitamin A				Antioxidant	0.27	0.91
Vitamin C				Antioxidant	0.27	0.09
Betaine				1-C metab	0.21	0.44
Methionine				1-C metab	0.21	0.24
Vitamin B_6_				1-C metab	0.21	0.12
Choline				1-C metab	0.21	0.08
Vitamin B_12_				1-C metab	0.21	0.07

Abbreviations: 1-C metab, 1-carbon metabolism pathway; BKMR, Bayesian kernel machine regression; SRS, Social Responsiveness Scale.

1 PIPs are from BKMR models of the association of prenatal nutrient intake with child SRS total raw score, adjusted for maternal age, maternal prepregnancy BMI, child sex, cohort type, maternal ethnicity/race, maternal education, maternal smoking, and child year of birth. PIPs are the probabilities for the inclusion of a nutrient in the Markov Chain Monte Carlo (MCMC) sampler, representing the relative importance of each exposure in the model. PIPs range from 0 to 1, with higher values representing higher importance. In the same way, group PIPs describe the relative importance of a group of nutrients. Conditional PIPs describe the relative strength of each nutrient within a group, as given by the percentage of iterations in which the MCMC sampler identifies each nutrient as the most important when the group is included in the model [22].

### Secondary analyses

#### BKMR analysis of 14 nutrients with SRS score

**FIGURE 3:**
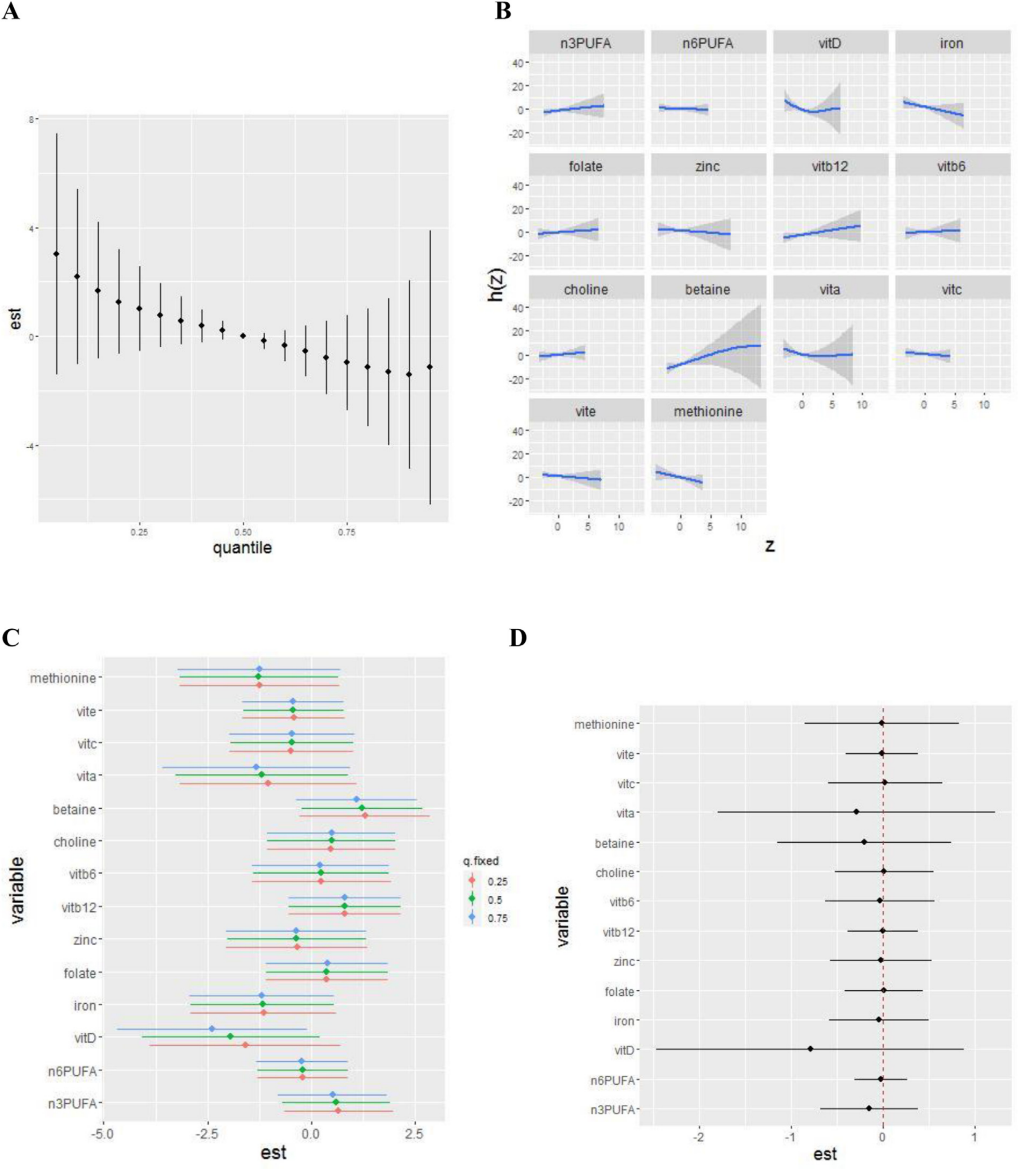
Adjusted associations between prenatal intake of 14 nutrients and child SRS scores using hierarchical Bayesian kernel machine regression (BKMR) (*n* = 952). Results of BKMR analyses in ECHO including energy-adjusted nutrient intake for omega 3 and omega 6 fatty acids, iron, vitamin D, folate, zinc, vitamin B_12_, vitamin B_6_, choline, betaine, vitamin A, vitamin C, vitamin E, and methionine, adjusted for maternal age, maternal prepregnancy BMI, child sex (male, female), cohort type (enriched familial autism probability, general population), maternal ethnicity/race (non-Hispanic White, non-Hispanic Black, Hispanic, and other), maternal education (less than high school, high school/GED, some college/associates degree/trade school, bachelor’s degree, and graduate degree), maternal smoking (yes, no), and child year of birth (1998–2004, 2005–2009, 2010–2014, and 2015+). Plots show: (A) the overall mixture effect, or the relationship between the nutrient mixture and child SRS scores. (B) Individual nutrient associations with SRS scores, holding all other nutrients at their 50th percentile. (C) Single-exposure effects; plot shows the impact on child SRS score when each exposure increases from its 25th to 75th percentile (whereas other exposures are fixed at their 25th, 50th, or 75th percentiles). (D) Interaction parameters; plot compares the single-exposure health risk of each exposure (associated with a change from its 25th to 75th percentile) when other exposures are fixed to their 75th percentile compared with their 25th percentile. The set of nutrients included here was selected as described in the text based on a priori interest for neurodevelopmental relevance and grouped by shared pathway. ECHO, Environmental influences on Child Health Outcomes; SRS, Social Responsiveness Scale.

**FIGURE 4:**
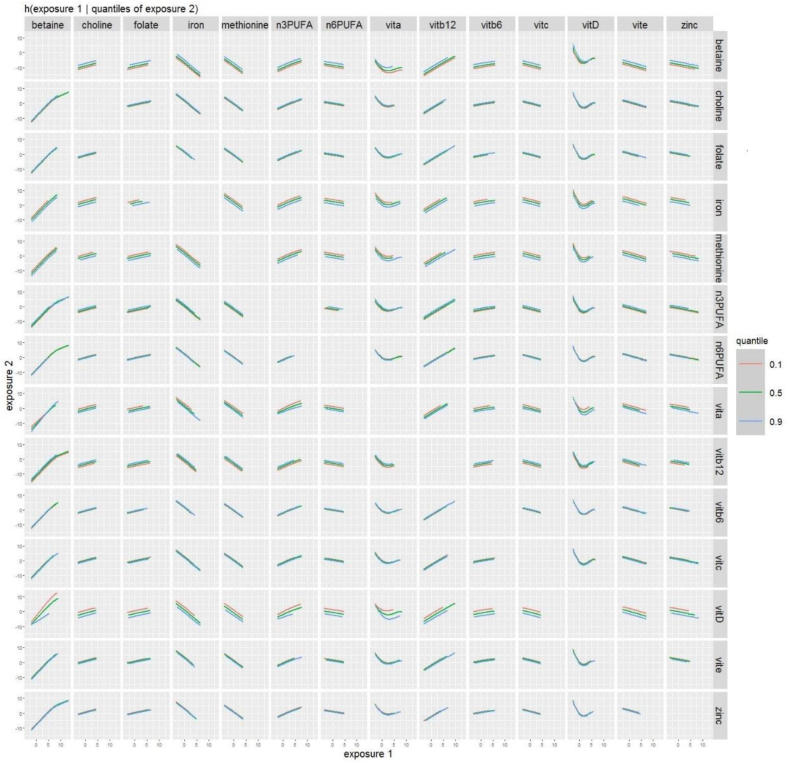
Adjusted bivariate associations between prenatal intake of 14 nutrients and child SRS scores using hierarchical Bayesian kernel machine regression (BKMR) (*n* = 952). Results of BKMR analyses in ECHO including energy-adjusted nutrient intake for omega 3 and omega 6 fatty acids, iron, vitamin D, folate, zinc, vitamin B_12_, vitamin B_6_, choline, betaine, vitamin A, vitamin C, vitamin E, and methionine, adjusted for maternal age, maternal prepregnancy BMI, child sex (male, female), cohort type (enriched familial autism probability, and general population), maternal ethnicity/race (non-Hispanic White, non-Hispanic Black, Hispanic, and other), maternal education (less than high school, high school/GED, some college/associates degree/trade school, bachelor’s degree, and graduate degree), maternal smoking (yes and no), and child year of birth (1998–2004, 2005–2009, 2010–2014 and 2015+). Plots show bivariate exposure–response function for 2 predictors, where the second predictor is fixed at various percentiles (10th, 50th, and 90th) and all other predictors are fixed at the 50th percentile. The set of nutrients included here was selected as described in the text based on a priori interest for neurodevelopmental relevance and grouped by shared pathway. ECHO, Environmental influences on Child Health Outcomes.

Compared with participants included in the primary analysis, maternal participants in the subsample with complete data on intake of 14 nutrients (4 cohorts, *n* = 952) were more likely to be non-Hispanic Black, have only a high school education, and to be drawn from an autism familial enriched risk cohort (Supplemental Table 4). Nutrient intakes were similar to those in the primary analysis (Supplemental Table 2). The association of the overall mixture with SRS scores remained similar in the expanded 14-nutrient mixture (Figure 3A). However, individual nutrient associations with SRS were altered. Previously suggested relationships with folate and omega 3 fatty acids were null in this nutrient mixture, and betaine was positively associated with SRS score, though credible intervals were wide (Figure 3B). Inverse associations of vitamin D with SRS were somewhat stronger when other nutrients were fixed at the 75th quantile (compared with 50th or 25th) (Figure 3C), but this difference had credible intervals that crossed the null (Figure 3D). There was no clear evidence of bivariate nutrient interactions (Figure 4). Assessing relative contributions in this nutrient mixture, the “inflammatory pathways” nutrient group had the highest group PIP, with vitamin D having the highest conditional PIP within the group, suggesting the strongest relationship with SRS score (Table 2). Within the “1 carbon metabolism” and “antioxidant” groups, betaine and vitamin A had the highest conditional PIPs, respectively.

#### BKMR analysis of nutrients with autism diagnosis

Most (91%) of the participants in the primary analyses also provided information on children’s autism diagnosis and were included in this secondary analysis. A total of 3280 dyads provided diagnostic information, with 137 children (4.2%) identified as autism cases. Participant characteristics were similar to those in the primary analysis (Supplemental Table 5). As in primary analyses, we conducted BKMR with a 5-nutrient mixture (7 cohorts, *n* = 3280) and a 14-nutrient mixture (4 cohorts, *n* = 1596); nutrient intakes across these groups were broadly comparable (Supplemental Table 6).

In covariate-adjusted probit BKMR models, credible intervals included the null for associations of the overall mixture and individual nutrients with an autism diagnosis, either when examining 5 nutrients (Supplemental Figure 3) or 14 nutrients (Supplemental Figure 4). When assessing relative contributions in these nutrient mixtures, the inflammatory group and omega 6 fatty acids had the highest group and conditional PIPs in the 5-nutrient analysis, respectively, and the inflammatory group and vitamin E and omega 6 fatty acids had the highest group and conditional PIPs in the 14-nutrient analysis (Supplemental Table 7).

#### Comparison analyses using traditional regression approaches

To compare with previous work assessing individual nutrients, we conducted regression analyses of single nutrients with autism outcomes. Using adjusted quantile regression models to examine the association of individual nutrients with SRS scores, statistically significant associations with SRS scores were identified for omega 3 and omega 6 fatty acid intake. Compared with the lowest quartile of intake, higher intake was consistently inversely associated with SRS score, with beta coefficients ranging from –1.63 to –3.91 (Supplemental Table 8). Folate intake above the first quartile was also consistently, inversely associated with SRS score; however, the results were not statistically significant. Given the suggestive evidence for an interaction of folate and omega 3 fatty acids in the primary BKMR model, we examined these 2 nutrients in an adjusted regression model together with an interaction term, but the term was not statistically significant (*P*-interaction = 0.98). When the expanded suite of 14 nutrients was examined in the subsample with complete data, vitamins A, B_6_, B_12_, D, and zinc had statistically significant inverse associations with SRS score in ≥1 quartile, with betas ranging from –2.18 to –5.40 (Supplemental Table 9). In adjusted logistic regression models, no nutrients were consistently significantly associated with autism diagnosis across intake quartiles, whether considering the primary or expanded sets of nutrients (Supplemental Tables 10 and 11).

#### Sensitivity analyses

In sensitivity analyses, the association of the overall mixture remained similar, but associations with individual nutrients changed. For example, when the primary 5-nutrient BKMR analysis was performed in the subsample with complete data on 14 nutrients (4 cohorts, *n* = 952), vitamin D and iron were inversely associated with SRS score, and folate was positively associated (Supplemental Figure 5). In analyses examining total intake of the 5 primary nutrients from diet and supplements (4 cohorts, *n* = 952; Supplemental Table 12), vitamin D was inversely associated with SRS score, and folate was positively associated with SRS score at higher folate intakes (Supplemental Figure 6). In both sensitivity analyses, there was no clear association with omega 3 fatty acids.

Given that these subsamples included only 4 ECHO cohorts (compared with 7), we examined whether associations changed with the exclusion of individual cohorts using sensitivity analyses excluding 1 cohort at a time. In quantile regression models, the removal of the large New Hampshire Birth Cohort Study (NHBCS) led to changes in estimates for associations of nutrients with SRS score (Supplemental Figure 7). In particular, associations with vitamin D became inverse rather than positive, and associations with folate and omega 6 fatty acids became null. Sensitivity analyses examining additional adjustment for covariates in regression models did not substantially alter results, with the exception of adjustment for FFQ type, which attenuated the significant inverse associations of omega 3 and omega 6 fatty acids with SRS score (Supplemental Table 13). In regression models stratified by FFQ type (not conducted for DHQ given small sample size), although there was some variability for certain nutrients across FFQs, overall patterns of associations with SRS and ASD were similar across stratified and full sample results (Supplemental Tables 14 and 15). When FFQ type was added as a covariate to the primary BKMR analysis, the overall mixture effect was attenuated (Supplemental Figure 8), although FFQ type differed by cohort (Supplemental Table 1).

## Discussion

In this large and geographically and demographically diverse sample from the ECHO cohort consortium, we found some evidence for associations of prenatal dietary nutrient intake with children’s autism outcomes. By employing a Bayesian mixtures approach, we examined complex relationships among a suite of nutrients while accounting for multicollinearity. In our primary models, we found evidence for U-shaped relationships of folate and omega 3 fatty acids with children’s autism-related traits, as well as a trend for lower SRS scores as the overall nutrient mixture—which included several nutrients important for neurodevelopment—increased. These results align with prior work suggesting the importance of prenatal intake of these nutrients for neurodevelopment and involvement in autism development. After expanding our models to include a larger suite of nutrients, associations with specific nutrients were altered, with vitamin D gaining importance in the model, suggesting the need to carefully consider the relative contributions of components of the diet and their potential combined effects.

To our knowledge, this is among the largest analyses of dietary exposures and neurodevelopmental outcomes using mixture models [24,48,49]. Previously, we performed BKMR analysis using data from the NHSII (*n* = 713) and the EARLI study (*n* = 127) [26]. In the EARLI familial autism cohort, there was no evidence for associations of maternal nutrient intake with children’s SRS scores; however, in the NHSII general population cohort, there was suggestive evidence for a positive association of omega 3 fatty acids and negative associations of omega 6 fatty acids, iron, and vitamin B_12_ with SRS score, as well as a trend for lower SRS score with increasing values of the overall nutrient mixture. In the current study, we did not find evidence for associations with the same nutrients; however, we did identify a similar trend in overall mixtures effects, underscoring the importance of taking a comprehensive look at diet and dietary patterns for neurodevelopment. Of note, the EARLI study was also included in this analysis, as it is an ECHO cohort study site. However, it contributed a relatively small sample and did not strongly influence results in leave-one-out sensitivity analysis.

There are several possible explanations for differences in the individual nutrient associations identified with BKMR, across studies and between the primary (5 nutrient) and secondary (14 nutrient) analyses of the current study. First, the differences may be because of the inclusion of different combinations of nutrients in BKMR models. The results of these models may be sensitive to the complex interrelationships characteristic of nutrient intake, including true biological interactions. This is a strength of mixtures modeling; however, researchers should be thoughtful when selecting nutrients to include in future analyses. For example, in this analysis, the relative importance of folate was reduced after the inclusion of other nutrients on the methylation pathway, especially betaine. In fact, betaine was identified as being individually associated with SRS score. Given that betaine and folate are complementary sources of 1-carbon units used in methylation pathways [50], researchers should consider the complex interplay of these nutrients in gene methylation and autism development.

Differences in associations identified by BKMR models could also be because of differences in sample characteristics, such as nutrient intake or SRS scores. In the current analysis, the exclusion of the NHBCS influenced associations with vitamin D, folate, and omega 6 fatty acids in traditional regression models. NHBCS is a largely rural cohort, with different access to food systems as well as health care. Participants also tended to be non-Hispanic White, with a relatively high level of education, in part due to the selection of pregnancies that were using private, unregulated water systems at their place of residence [51]. Despite these differences, nutrient intake distributions and SRS scores were similar between this cohort and others included in this analysis. Furthermore, we included adjustments for sample characteristics including race, ethnicity, and education. It is possible that these results are because of the reduced sample size of models excluding NHBCS, as this was also the largest cohort included in the analysis, contributing nearly 40% of participants.

Differing results could also be because of variations in assessment methodology across cohorts. For example, this analysis used dietary data collected from 3 different validated FFQs. The 14-nutrient sample data were all drawn from the Block FFQ, whereas the 5-nutrient sample had greater contributions from the Harvard/Willett FFQ. Though prior work supports the comparability of nutrient data from these sources [37], minor measurement differences may have contributed to variability. We also observed differences in the overall mixture effect when further adjusting for diet form type, although this was also highly related to cohort and may have inflated variance in coadjustment, particularly in standard models.

Most previous work examining links between prenatal diet and children’s autism development has used traditional single-exposure regression models, rather than mixtures models. A description of this work, focusing on folate, fatty acids, and vitamin D, is included next. In our analysis, associations of certain nutrients with autism outcomes were present in traditional regression models but not in mixtures models. This suggests that concerns of coexposure confounding across nutrients may impact traditional analyses of nutrient-autism associations. Mixtures models may be better able to handle complex associations between nutrients and within metabolic pathways.

Despite the challenges of traditional regression models, studies using these models have also identified similar associations between folate, omega 3 fatty acids, and vitamin D with autism outcomes as those identified by mixtures models here. Folate, in particular, has been studied for its role in autism development [52]. Previous work has generally reported negative associations between supplemental folic acid intake and autism diagnosis or traits [12,53], although there is also evidence for positive associations at the highest intake level [13]. In this study, we found evidence for a U-shaped relationship between dietary folate and SRS score, supporting previous research, although credible intervals were wide at higher folate intakes. Folate is thought to influence neurodevelopment primarily via its involvement in the 1-carbon metabolism pathway, which impacts gene methylation. Other nutrients on the 1-carbon metabolism pathway have been studied less. Prenatal betaine, and its precursor choline, have been positively associated with children’s neurodevelopment [54,55]. Evidence from animal models suggests that these nutrients may also influence autism development specifically [[56], [57], [58]]. Although there are no United States recommendations for betaine intake during pregnancy, insufficient intake of choline during pregnancy is common [59,60]. Given that betaine was associated with SRS score in this analysis, further research including all nutrients on the methylation pathway is recommended.

Omega 3 and 6 fatty acids were also associated with autism outcomes in this study and in traditional analyses [16,17]. These fatty acids are crucial for membrane formation, cell signaling, and inflammatory processes. In our analysis, we found a U-shaped association of omega 3 fatty acids with autism trait scores and a possible interaction with folate in the 5-nutrient analysis. Our data suggest that higher prenatal intake of omega 3 fatty acids may modestly increase both the inverse association (at moderate intake levels) and positive association (at extreme intake levels) of folate with SRS score. Other studies have noted a link between folate and omega 3 fatty acids (especially DHA). For example, maternal plasma levels of folate and DHA are positively associated during pregnancy [61]. Additionally, animal models have noted improvements in cognitive performance after prenatal co-supplementation of B vitamins (including folate) and fatty acids, above supplementation with a single nutrient [62]. However, to our knowledge, no other studies have identified this interaction in relation to autism development.

In this study, there was suggestive evidence for a negative association of dietary vitamin D with SRS score in mixtures models, similar to previous literature using traditional regression models [[18], [19], [20]], but only when a wider suite of nutrients was also included in the model. Perhaps this could be because of correlated food sources of certain nutrients (that is, fish as a source of both omega 3s and vitamin D), although the diet is not generally the major contributor to vitamin D status. Mechanistic studies of the role of vitamin D in the context of other nutrients may be needed to explain this finding more clearly.

Although this work represents the largest and most detailed BKMR mixtures analysis of nutrient intake and autism outcomes to date, several limitations apply. First, cohorts differed in the timing and type of FFQ used for dietary assessment. Although pooling of data is a major strength of the ECHO consortium, these differences may have contributed to our varying results across primary and secondary analyses, as noted above. Differences across dietary assessment methods also meant that we were not able to assess potential differences by trimester of pregnancy and could examine nutrient intake from supplements in only a subset of participants. Nutrient supplements are a major source of some of our primary nutrients of interest [47], and prenatal vitamin use has been consistently associated with autism development [12,53]. In sensitivity analyses with supplemental intake, differences in individual nutrient associations were noted, suggesting future analyses should further explore the role of nutrient supplements in complex nutrient-SRS associations. Because data collection continues in the ECHO study under a standardized protocol, harmonized information on dietary intake, supplement use, and nutrient biomarkers will become more widely available [29]. Additionally, all dietary intake assessments are subject to error and bias, and FFQs tend to overestimate intake [32]; however, we reduced the potential for these errors and biases by using only prospectively collected, validated FFQs and adjusting for energy intake.

Another limitation is that several cohorts only provided parent reports of physician autism diagnoses, without clinical confirmation. This may partly explain why we did not find similar associations of nutrients with autism diagnosis as with SRS score. However, 49% of our sample also conducted clinical assessments or medical record abstraction to confirm autism diagnosis, and results were similar in these cohorts. Alternatively, it may be that quantitative autism trait scores are more sensitive to associations with nutrients across the distribution.

Finally, without mechanistic studies, we cannot be certain that the differences in nutrient associations seen in the 5-nutrient primary analysis and 14-nutrient secondary analysis were because of true biological interactions rather than statistical modeling effects or differences in sample characteristics. As models evolve and improve, and as animal and human evidence increases, we may have a better understanding of the complex interrelationships of nutrients within and across pathways that influence neurodevelopment.

In conclusion, this study highlights the use of mixtures models to explore the interrelated associations of prenatal nutrient intakes with autism development. Using these models, we found evidence for individual nutrient associations previously documented using traditional regression analysis, as well as novel nutrient interactions. Continued examination of the complex associations between nutrients and autism development using mixtures models, complemented by mechanistic studies, can help refine our understanding of nutrition’s impact on neurodevelopment.

## Author contributions

The authors’ responsibilities were as follows – KNC, SME, MRK, P-IL, RJS, KL: directed cohort-level data collection; KL: designed the analysis; JR: performed statistical analysis; MGB: aided in study oversight, wrote the paper, and had primary responsibility for final content; and all authors: read and approved the final manuscript.

## Data availability

Select deidentified data from the ECHO Program are available through NICHD’s Data and Specimen Hub (DASH). Information on study data not available on DASH, such as some Indigenous datasets, can be found on the ECHO study DASH webpage.

## Funding

Research reported in this publication was supported by the Environmental influences on Child Health Outcomes (ECHO) Program, Office of the Director, National Institutes of Health, under Award Numbers U2COD023375 (Coordinating Center), U24OD023382 (Data Analysis Center), U24OD023319 with cofunding from the Office of Behavioral and Social Science Research (Measurement Core), U24OD035523 (Lab Core), ES0266542 (HHEAR), U24ES026539 (HHEAR Barbara O’Brien), U2CES026533 (HHEAR Lisa Peterson), U2CES026542 (HHEAR Patrick Parsons, Kannan Kurunthacalam), U2CES030859 (HHEAR Manish Arora), U2CES030857 (HHEAR Timothy R. Fennell, Susan J. Sumner, Xiuxia Du), U2CES026555 (HHEAR Susan L. Teitelbaum), U2CES026561 (HHEAR Robert O. Wright), U2CES030851 (HHEAR Heather M. Stapleton, P. Lee Ferguson), UG3/UH3OD023251 (Akram Alshawabkeh), UH3OD023320 and UG3OD035546 (Judy Aschner), UH3OD023332 (Clancy Blair, Leonardo Trasande), UG3/UH3OD023253 (Carlos Camargo), UG3/UH3OD023248 and UG3OD035526 (Dana Dabelea), UG3/UH3OD023313 (Daphne Koinis Mitchell), UH3OD023328 (Cristiane Duarte), UH3OD023318 (Anne Dunlop), UG3/UH3OD023279 (Amy Elliott), UG3/UH3OD023289 (Assiamira Ferrara), UG3/UH3OD023282 (James Gern), UH3OD023287 (Carrie Breton), UG3/UH3OD023365 (Irva Hertz-Picciotto), UG3/UH3OD023244 (Alison Hipwell), UG3/UH3OD023275 (Margaret Karagas), UH3OD023271 and UG3OD035528 (Catherine Karr), UH3OD023347 (Barry Lester), UG3/UH3OD023389 (Leslie Leve), UG3/UH3OD023344 (Debra MacKenzie), UH3OD023268 (Scott Weiss), UG3/UH3OD023288 (Cynthia McEvoy), UG3/UH3OD023342 (Kristen Lyall), UG3/UH3OD023349 (Thomas O’Connor), UH3OD023286 and UG3OD035533 (Emily Oken), UG3/UH3OD023348 (Mike O’Shea), UG3/UH3OD023285 (Jean Kerver), UG3/UH3OD023290 (Julie Herbstman), UG3/UH3OD023272 (Susan Schantz), UG3/UH3OD023249 (Joseph Stanford), UG3/UH3OD023305 (Leonardo Trasande), UG3/UH3OD023337 (Rosalind Wright), UG3OD035508 (Sheela Sathyanarayana), UG3OD035509 (Anne Marie Singh), UG3OD035513 and UG3OD035532 (Annemarie Stroustrup), UG3OD035516 and UG3OD035517 (Tina Hartert), UG3OD035518 (Jennifer Straughen), UG3OD035519 (Qi Zhao), UG3OD035521 (Katherine Rivera-Spoljaric), UG3OD035527 (Emily S Barrett), UG3OD035540 (Monique Marie Hedderson), UG3OD035543 (Kelly J Hunt), UG3OD035537 (Sunni L Mumford), UG3OD035529 (Hong-Ngoc Nguyen), UG3OD035542 (Hudson Santos), UG3OD035550 (Rebecca Schmidt), UG3OD035536 (Jonathan Slaughter), UG3OD035544 (Kristina Whitworth).

## Conflict of interest

The content is solely the responsibility of the authors and does not necessarily represent the official views of the National Institutes of Health. The authors report no conflicts of interest.
