# Supplementary material for: The Association of Prenatal Dietary Factors with Child Autism Diagnosis and Autism-Related Traits Using a Mixtures Approach: Results from the Environmental Influences on Child Health Outcomes Cohort
Source: J Nutr. 2025 Mar 17;155(6):1938–51. doi: 10.1016/j.tjnut.2025.02.025 (PMC12264552; doi:10.1016/j.tjnut.2025.02.025)
Supplement: multimedia component 1 [file mmc1.docx]

The association of prenatal dietary factors with child autism diagnosis and autism-related traits using a mixtures approach: Results from the ECHO Cohort

Megan G Bragg, et al.

Supplementary Materials

Supplemental Table 1 – ECHO cohort study sites included in this analysis of nutrients and autism outcomes.

Supplemental Table 2 – Comparison of nutrient intake values of ECHO participants included in the primary analysis of 5 nutrients versus 14 nutrients.

Supplemental Table 3 – Comparison of mean (SD) nutrient intake values of ECHO participants included in the primary analysis of 5 nutrients by cohort (n=2,614).

Supplemental Table 4 – Characteristics of ECHO participants included in the primary analysis of nutrient intake and children’s Social Responsiveness Scale scores, comparing the 5-nutrient and 14-nutrients samples.

Supplemental Table 5 – Characteristics of ECHO participants in the secondary analyses of nutrient intake and autism diagnosis, comparing the 5-nutrient and 14-nutrient samples.

Supplemental Table 6 – Comparison of nutrient intake values of ECHO participants included in the secondary analyses of nutrient intake and autism diagnosis, comparing the 5-nutrient and 14-nutrient samples.

Supplemental Table 7 - Posterior Inclusion Probabilities from adjusted, hierarchical BKMR with child autism diagnosis in 5 nutrient and 14 nutrient analyses.

Supplemental Table 8 - Association between maternal intake of 5 nutrients during pregnancy and child SRS raw scores using quantile regression at the 50th percentile of SRS scores (n=2,614).

Supplemental Table 9 - Association between maternal nutrient intake during pregnancy and child SRS raw scores using quantile regression at the 50th percentile of SRS scores in the 14-nutrient subsample (n=952).

Supplemental Table 10 - Association between maternal intake of 5 nutrients during pregnancy and child autism diagnosis using logistic regression (n=3280).

Supplemental Table 11 - Association between maternal intake of 14 nutrients during pregnancy and child autism diagnosis using logistic regression (n= 1596).

Supplemental Table 12 – Intake from supplements among nutrients included in sensitivity analysis of total nutrient intake with SRS scores (n=952)**.**

Supplemental Table 13 – Association between maternal intake of 5 nutrients during pregnancy and child SRS raw scores using quantile regression at the 50th percentile, with additional adjustment for covariates.

Supplemental Table 14 – Association between maternal nutrient intake during pregnancy and child SRS raw scores in the 5-nutrient sample using adjusted quantile regression (SRS at 50^th^ percentile), stratified by stratified by food frequency questionnaire (FFQ).

Supplemental Table 15 – Association between maternal nutrient intake during pregnancy and child autism diagnosis in the 5-nutrient sample using adjusted logistic regression, stratified by food frequency questionnaire (FFQ).

Supplemental Figure 1 – Participant selection flow chart for this analysis of nutrient and autism outcomes in the ECHO cohort.

Supplemental Figure 2 – Heat maps of Spearman correlations of nutrients in the a) 5-nutrient sample and b) 14-nutrient sample.

Supplemental Figure 3 - Adjusted associations between prenatal intake of 5 nutrients and child autism diagnosis using hierarchical BKMR (n=3280).

Supplemental Figure 4 - Adjusted associations between prenatal intake of 14 nutrients and child autism diagnosis using hierarchical BKMR (n=1596).

Supplemental Figure 5 - Adjusted associations between prenatal intake of 5 nutrients and child SRS scores using hierarchical BKMR, limited to the subsample with complete data on 14 nutrients (n=952).

Supplemental Figure 6 - Adjusted associations between prenatal intake of 5 nutrients (from diet and supplements) and child SRS scores using hierarchical BKMR (n=952).

Supplemental Figure 7 – Leave-one-out analyses examining the influence of excluding individual ECHO cohorts on the association between maternal intake of 5 nutrients during pregnancy and child SRS raw scores using quantile regression at the 50th percentile of SRS scores.

Supplemental Figure 8 - Adjusted associations between prenatal intake of 5 nutrients and child SRS scores using hierarchical BKMR, with additional adjustment for food frequency questionnaire type (n= 2614).

Appendix A. ECHO Collaborators

Supplemental Table 1 – ECHO cohort study sites included in this analysis of nutrients and autism outcomes.

| **Cohort name** | **N included in primary analysis** | **Participants** | **Prenatal Food Frequency Questionnaire (FFQ)** | **Child autism outcomes** |
| --- | --- | --- | --- | --- |
| Atlanta ECHO Cohort of Emory University ^a^ | 68 | Pregnancy cohort of healthy African American women | Block FFQ administered during early pregnancy (8-14 weeks gestation) and mid-pregnancy (24-30 weeks gestation) | SRS; parent-reported autism diagnosis confirmed by medical record |
| Conditions Affecting Neurocognitive Development and Learning in Early Childhood (CANDLE) ^a^ | 600 | Pregnancy cohort from the general population with a singleton, low medical risk pregnancy | Block FFQ administered during the 2^nd^ trimester of pregnancy; assessed dietary intakes in the past 3 months | SRS; parent-reported autism diagnosis |
| University of California – Markers of Autism Risk in Babies  (MARBLES) ^a^ | 131 | Pregnancy cohort of families who already had a child with autism | Block FFQ administered at mid-pregnancy and end of pregnancy | SRS; clinical autism diagnosis |
| PRogramming of Intergenerational Stress Mechanisms (PRISM) ^a^ | 153 | Pregnancy cohort of healthy women | Modified Block98 FFQ administered during the 2^nd^ trimester; assessed dietary intakes in the past 3 months | SRS; parent-reported autism diagnosis |
| New Hampshire Birth Cohort Study (NHBCS) | 997 | Pregnancy cohort from the general population of a rural region & restricted to those with private water systems | Harvard/Willett FFQ administered at mid-pregnancy (~24-28 weeks gestation) | SRS; autism diagnosis confirmed by medical record |
| Project Viva | 574 | Pregnancy cohort of healthy women | Harvard/Willett FFQ administered at 1^st^ trimester visit | SRS; parent-reported autism diagnosis |
| Early Autism Risk Longitudinal Investigation (EARLI) | 91 | Pregnancy cohort of families who already had a child with autism | Diet History Questionnaire II administered at mid-pregnancy (20 weeks gestation) and end of pregnancy | SRS; clinical autism diagnosis |

SRS – Social Responsiveness Scale

^a^ Data from these cohorts were included in the secondary analysis of 14 nutrients.

Supplemental Table 2 – Comparison of nutrient intake values of ECHO participants included in the primary analysis of 5 nutrients versus 14 nutrients.^a^

|  | **Recommended Dietary Allowance or Adequate Intake (*) for Pregnancy** | **5-nutrient sample**  **(n=2614)** | **14-nutrient sub-sample**  **(n = 952)** |
| --- | --- | --- | --- |
| **Nutrient** |  | **Mean (SD)** | |
| Dietary Folate (mcg/d DFE) | 600 | 518.96 (251.40) | 613.27 (295.28) |
| Dietary Vitamin D (mcg/d) | 15* | 5.49 (3.69) | 4.15 (2.95) |
| Dietary Omega 3 fatty acids (g/d) |  | 1.56 (0.81) | 1.81 (0.94) |
| Dietary Omega 6 fatty acids (g/d) |  | 13.48 (6.41) | 15.67 (7.67) |
| Dietary Iron (mg/d) | 27 | 16.0 (6.96) | 15.74 (7.10) |
| Dietary Zinc (mg/d) | 11 | 12.47 (5.09) | 11.75 (5.19) |
| Dietary Vitamin B12 (mcg/d) | 2.6 | 6.01 (3.56) | 5.36 (3.02) |
| Dietary Vitamin B6 (mg/d) | 1.9 | 2.20 (0.91) | 2.11 (0.97) |
| Dietary Choline (mg/d) | 450* | 339.81 (146.01)^b^ | 320.97 (154.73) |
| Dietary Betaine (mg/d) |  | 154.13 (105.56)^c^ | 192.65 (129.83) |
| Dietary Vitamin A (mcg RAE/d) | 770 | 960.17 (502.87)^d^ | 909.11 (497.50) |
| Dietary Vitamin C (mg/d) | 85 | 149.73 (89.25) | 152.84 (93.33) |
| Dietary Vitamin E (mg ATE/d) | 15 | 8.56 (6.03)^e^ | 8.50 (3.83) |
| Dietary Methionine (g/d) |  | 1.85 (0.75) | 1.63 (0.77) |

^a^ Values in this table are not energy adjusted; however, energy adjustment was performed prior to analysis.

^b^n=1949

^c^n=1949

^d^n=2418

^e^n=1617

Supplemental Table 3 – Comparison of mean (SD) nutrient intake values of ECHO participants included in the primary analysis of 5 nutrients by cohort (n=2,614).^a^

|  | **Atlanta ECHO^b^**  **(n=68)** | **CANDLE^b^**  **(n=600)** | **MARBLES^b^**  **(n=131)** | **PRISM^b^**  **(n=153)** | **NHBCS**  **(n=997)** | **Project Viva (n=574)** | **EARLI**  **(n=91)** |
| --- | --- | --- | --- | --- | --- | --- | --- |
| Total Energy (mcg/d DFE) | 1831.10 (854.94) | 2321.96 (980.21) | 1596.82 (600.99) | 2035.61 (775.94) | 2092.93 (678.22) | 2088.44 (638.80) | 1919.79 (861.54) |
| Dietary Folate (mcg/d DFE) | 520.11 (272.0) | 655.08 (305.07) | 464.63 (195.70) | 617.98 (289.04) | 500.43 (194.16) | 387.24 (175.10) | 566.29 (300.63) |
| Dietary Vitamin D (mcg/d) | 3.43 (2.57) | 4.42 (3.00) | 3.30 (2.26) | 4.10 (3.24) | 6.75 (4.14) | 5.73 (3.27) | 4.14 (2.87) |
| Dietary Omega 3 fatty acids (g/d) | 1.55 (0.78) | 1.90 (0.94) | 1.35 (0.78) | 1.95 (0.98) | 1.49 (0.74) | 1.35 (0.59) | 1.14 (0.52) |
| Dietary Omega 6 fatty acids (g/d) | 13.27 (6.90) | 16.91 (8.01) | 11.90 (5.55) | 15.08 (6.86) | 12.72 (5.15) | 11.24 (4.82) | 12.14 (6.07) |
| Dietary Iron (mg/d) | 13.16 (6.47) | 17.15 (7.40) | 11.61 (4.65) | 14.88 (6.0) | 15.37 (6.21) | 17.50 (7.63) | 16.09 (7.62) |
| Dietary Zinc (mg/d) | 9.70 (4.96) | 12.67 (5.33) | 8.92 (3.81) | 11.48 (4.62) | 13.03 (4.55) | 12.78 (5.59) | 11.76 (5.29) |
| Dietary Vitamin B12 (mcg/d) | 4.55 (2.55) | 5.77 (3.14) | 3.91 (2.12) | 5.37 (2.91) | 6.45 (3.21) | 6.65 (4.70) | 4.09 (2.10) |
| Dietary Vitamin B6 (mg/d) | 1.89 (1.03) | 2.27 (0.97) | 1.57 (0.65) | 2.05 (0.96) | 2.24 (0.82) | 2.32 (0.91) | 1.95 (0.96) |
| Dietary Choline (mg/d) | 276.33 (146.32) | 343.67 (164.07) | 243.20 (100.99) | 318.38 (131.93) | 357.79 (134.81) |  |  |
| Dietary Betaine (mg/d) | 178.27 (124.17) | 205.55 (139.55) | 144.74 (89.83) | 189.50 (110.78) | 117.35 (54.11) |  |  |
| Dietary Vitamin A (mcg RAE/d) | 875.48 (537.06) | 971.91 (512.89) | 663.96 (346.33) | 887.69 (462.41) | 1011.39 (457.73)^c^ | 1001.42 (565.01) | 783.19 (433.15) |
| Dietary Vitamin C (mg/d) | 148.05 (95.52) | 167.93 (95.12) | 96.42 (65.51) | 144.11 (85.93) | 125.75 (69.63) | 188.08 (95.46) | 137.97 (107.43) |
| Dietary Vitamin E (mg ATE/d) | 7.64 (4.35) | 8.79 (3.72) | 6.94 (3.34) | 9.11 (4.01) |  | 8.82 (8.69) | 7.42 (3.97) |
| Dietary Methionine (g/d) | 1.32 (0.67) | 1.78 (0.80) | 1.17 (0.48) | 1.56 (0.67) | 1.92 (0.69) | 2.10 (0.72) | 1.68 (0.77) |

^a^ Values in this table are not energy adjusted; however, energy adjustment was performed prior to analysis.

^b^ Included in 14-nutrient subsample

^c^ n=801Supplemental Table 4 – Characteristics of ECHO participants included in the primary analysis of nutrient intake and children’s Social Responsiveness Scale scores, comparing the 5-nutrient and 14-nutrients samples.

|  | **5-nutrient sample^a^**  **(n=2614)** | **14-nutrient sub-sample^b^**  **(n = 952)** |
| --- | --- | --- |
|  | **N (%)** | |
| **Cohort Type** |  |  |
| Autism (familial) enriched risk | 222 (8.49) | 131 (13.76) |
| Non-enriched/population-based | 2392 (91.51) | 821 (86.24) |
| **Maternal Race/Ethnicity** |  |  |
| Non-Hispanic White | 1719 (65.76) | 344 (36.13) |
| Non-Hispanic Black | 536 (20.50) | 434 (45.59) |
| Hispanic | 178 (6.81) | 97 (10.19) |
| Other | 176 (6.73) | 77 (8.09) |
| Missing | 5 (0.19) | 0 |
| **Maternal Smoking** |  |  |
| Yes | 145 (5.55) | 76 (7.98) |
| No | 2448 (93.65) | <900 |
| Missing | 21 (0.8) | <5 |
| **Prenatal Supplement Use (Ever)** |  |  |
| Yes | 2102 (80.41) | 862 (90.55) |
| No | 134 (5.13) | 63 (6.62) |
| Missing | 378 (14.46) | 27 (2.84) |
| **Maternal Education** |  |  |
| <High school | 102 (3.9) | 85 (8.93) |
| High school degree, GED | 427 (16.34) | 306 (32.14) |
| Some college/ Associate’s/ Trade school | 653 (24.98) | 151 (15.86) |
| Bachelor’s degree | 648 (24.79) | 244 (25.63) |
| Master’s, Professional or Doctorate Degree | 704 (26.93) | 150 (15.76) |
| Missing | 80 (3.06) | 16 (1.68) |
| **Household Income** |  |  |
| <$30,000 | 225 (8.61) | 192 (20.17) |
| $30,000-$49,999 | 142 (5.43) | 127 (13.34) |
| $50,000-$74,999 | 188 (7.19) | 99 (10.4) |
| $75,000-$99,999 | 214 (8.19) | 90 (9.45) |
| $100,000 or more | 767 (29.34) | 240 (25.21) |
| Missing | 1078 (41.24) | 204 (21.43) |
| **Child Sex** |  |  |
| Male | 1313 (50.23) | 494 (51.89) |
| Female | 1301 (49.77) | 458 (48.11) |
| **Preterm Birth** |  |  |
| Term (≥37 weeks gestation) | 2451 (93.76) | 884 (92.86) |
| Preterm (<37 gestation) | <165 | 68 (7.14) |
| Missing | <5 |  |
| **Child birth year** |  |  |
| 1998-2004 | 574 (21.96) | 0 |
| 2005-2009 | 453 (17.333) | 353 (37.08) |
| 2010-2014 | 1261 (48.24) | 471 (49.47) |
| 2015+ | 326 (12.47) | 128 (13.45) |
| **Breastfed (Ever)** |  |  |
| Yes | 1546 (59.14) | 343 (36.03) |
| No | 24 (0.92) | 21 (2.21) |
| Missing | 1044 (39.94) | 588 (61.76) |
| **Autism Diagnosis** |  |  |
| Autism | 107 (4.09) | 56 (5.88) |
| No autism | 2285 (87.41) | 873 (91.7) |
| Missing | 222 (8.49) | 23 (2.42) |
| **SRS Instrument** |  |  |
| 16 items | 380 (14.54) | 380 (39.92) |
| 65 items | 2234 (85.46) | 572 (60.08) |
| **SRS Form Type** |  |  |
| School Age | 2123 (81.22) | 809 (84.98) |
| Preschool | 491 (18.78) | 143 (15.02) |
| **Diet Form Type** |  |  |
| Block | 952 (36.42) | 952 (36.42) |
| DHQ2 | 91 (3.48) | - |
| Harvard | 1571 (60.10) | - |
|  | **Mean (SD)** | |
| Maternal Age | 31.30 (5.42) | 29.19 (5.85) |
| Birthweight | 3393.05 (541.70) | 3275.65 (535.49) |
| Pre-pregnancy BMI | 26.08 (6.37) | 27.69 (7.53) |
| Parity | 0.95 (1.03) | 1.11 (1.12) |
| SRS Total Raw Score | 25.17 (20.62) | 23.03 (22.86) |
| SRS Total T score | 46.60 (8.03) | 45.91 (8.74) |

SRS – Social Responsiveness Scale

^a^Cohorts: Atlanta Echo Emory, Conditions Affecting Neurocognitive Development and Learning in Early Childhood (CANDLE), Markers of Autism Risk in Babies (MARBLES), Programming of Intergenerational Stress Mechanisms (PRISM), New Hampshire Birth Cohort Study (NHBCS), Project Viva, Early Autism Risk Longitudinal Investigation (EARLI)

^b^Cohorts: Atlanta Echo Emory, Conditions Affecting Neurocognitive Development and Learning in Early Childhood (CANDLE), Markers of Autism Risk in Babies (MARBLES), Programming of Intergenerational Stress Mechanisms (PRISM)

Supplemental Table 5 – Characteristics of ECHO participants in the secondary analyses of nutrient intake and autism diagnosis, comparing the 5-nutrient and 14-nutrient samples.

|  | **5-nutrient sample**^a^  **(n = 3280)** | **14-nutrient sub-sample** ^b^  **(n=1596)** |
| --- | --- | --- |
|  | **N (%)** | |
| **Cohort Type** |  |  |
| Autism (familial) enriched risk | 289 (8.81) | 189 (11.84) |
| Non-enriched/population-based | 2991 (91.19) | 1407 (88.16) |
| **Maternal Race/Ethnicity** |  |  |
| Non-Hispanic White | 1900 (57.93) | 524 (32.83) |
| Non-Hispanic Black | 912 (27.80) | 806 (50.50) |
| Hispanic | 250 (7.62) | 158 (9.90) |
| Other | 213 (6.49) | 108 (6.77) |
| Missing | 5 (0.15) | 0 |
| **Maternal Smoking** |  |  |
| Yes | 203 (6.19) | 132 (8.27) |
| No | 3055 (93.14) | <1500 |
| Missing | 22 (0.67) | <5 |
| **Prenatal Supplement Use (Ever)** |  |  |
| Yes | 2711 (82.65) | 1462 (91.6) |
| No | 165 (5.03) | 95 (5.95) |
| Missing | 404 (12.32) | 39 (2.44) |
| **Maternal Education** |  |  |
| <High school | 178 (5.43) | 158 (9.9) |
| High school degree, GED | 686 (20.91) | 553 (34.65) |
| Some college/ Associate’s/ Trade school | 812 (24.76) | 262 (16.42) |
| Bachelor’s degree | 747 (22.77) | 372 (23.31) |
| Master’s, Professional or Doctorate Degree | 779 (23.75) | 230 (14.41) |
| Missing | 78 (2.38) | 21 (1.32) |
| **Household Income** |  |  |
| <$30,000 | 349 (10.64) | 316 (19.8) |
| $30,000-$49,999 | 180 (5.49) | 165 (10.34) |
| $50,000-$74,999 | 235 (7.16) | 144 (9.02) |
| $75,000-$99,999 | 245 (7.47) | 120 (7.52) |
| $100,000 or more | 875 (26.68) | 326 (20.43) |
| Missing | 1396 (42.56) | 525 (32.89) |
| **Child Sex** |  |  |
| Male | 1644 (50.12) | 818 (51.25) |
| Female | 1636 (49.88) | 778 (48.75) |
| **Preterm Birth** |  |  |
| Term (≥37 weeks gestation) | 3054 (93.11) | 1474 (92.36) |
| Preterm (<37 gestation) | <225 | 122 (7.64) |
| Missing | <5 |  |
| **Child birth year** |  |  |
| 1998-2004 | 670 (20.43) | 0 |
| 2005-2009 | 714 (21.77) | 615 (38.53) |
| 2010-2014 | 1502 (45.79) | 746 (46.74) |
| 2015+ | 394 (12.01) | 235 (14.72) |
| **Breastfed (Ever)** |  |  |
| Yes | 1761 (53.69) | 584 (36.59) |
| No | 44 (1.34) | 41 (2.57) |
| Missing | 1475 (44.97) | 971 (60.84) |
| **Child Autism Diagnosis** |  |  |
| Autism | 137 (4.18) | 80 (5.01) |
| No autism | 3143 (95.82) | 1516 (94.99) |
| Missing |  |  |
| **Diet Form Type** |  |  |
| Block | 1596 (48.66) | 1596 (48.66) |
| DHQ2 | 100 (3.05) | - |
| Harvard | 1584 (48.29) | - |
|  | **Mean (SD)** | |
| Maternal Age | 30.60 (5.65) | 28.67 (5.83) |
| Birthweight | 3368.23 (549.87) | 3266.13 (539.91) |
| Pre-pregnancy BMI | 26.39 (6.66) | 27.73 (7.61) |
| Parity | 0.97 (1.08) | 1.08 (1.15) |
| SRS Total Raw Score | 24.80 (20.59) | 22.49 (22.22) |
| SRS Total T score | 46.64 (8.03) | 45.76 (8.55) |

SRS – Social Responsiveness Scale

^a^Cohorts: Atlanta Echo Emory, Conditions Affecting Neurocognitive Development and Learning in Early Childhood (CANDLE), Markers of Autism Risk in Babies (MARBLES), Programming of Intergenerational Stress Mechanisms (PRISM), New Hampshire Birth Cohort Study (NHBCS), Project Viva, Early Autism Risk Longitudinal Investigation (EARLI)

^b^Cohorts: Atlanta Echo Emory, Conditions Affecting Neurocognitive Development and Learning in Early Childhood (CANDLE), Markers of Autism Risk in Babies (MARBLES), Programming of Intergenerational Stress Mechanisms (PRISM)

Supplemental Table 6 – Comparison of nutrient intake values of ECHO participants included in the secondary analyses of nutrient intake and autism diagnosis, comparing the 5-nutrient and 14-nutrient samples.

|  | **Recommended Dietary Allowance or Adequate Intake (*) for Pregnancy** | **5-nutrient sample** ^a^  **(n = 3280)** | **14-nutrient sub-sample** ^b^  **(n=1596)** |
| --- | --- | --- | --- |
| **Nutrient** |  | **Mean (SD)** | |
| Dietary Folate (mcg/d DFE) | 600 | 537.63 (273.98) | 622.49 (308.89) |
| Dietary Vitamin D (mcg/d) | 15* | 5.19 (3.52) | 4.17 (2.88) |
| Dietary Omega 3 fatty acids (g/d) |  | 1.61 (0.84) | 1.84 (0.95) |
| Dietary Omega 6 fatty acids (g/d) |  | 13.96 (6.88) | 16.03 (7.88) |
| Dietary Iron (mg/d) | 27 | 16.11 (7.24) | 16.01 (7.39) |
| Dietary Zinc (mg/d) | 11 | 12.40 (5.32) | 11.98 (5.47) |
| Dietary Vitamin B12 (mcg/d) | 2.6 | 5.92 (3.54) | 5.48 (3.13) |
| Dietary Vitamin B6 (mg/d) | 1.9 | 2.20 (0.94) | 2.15 (1.02) |
| Dietary Choline (mg/d) | 450* | 336.18 (148.63)^c^ | 326.14 (155.75) |
| Dietary Betaine (mg/d) |  | 168.19 (125.04) ^d^ | 198.18 (143.22) |
| Dietary Vitamin A (mcg RAE/d) | 770 | 950.74 (501.57) ^e^ | 913.22 (501.80) |
| Dietary Vitamin C (mg/d) | 85 | 152.75 (92.85) | 155.31 (97.49) |
| Dietary Vitamin E (mg ATE/d) | 15 | 8.63 (5.99) ^f^ | 8.61 (4.06) |
| Dietary Methionine (g/d) |  | 1.82 (0.76) | 1.66 (0.79) |

^a^Cohorts: Atlanta Echo Emory, Conditions Affecting Neurocognitive Development and Learning in Early Childhood (CANDLE), Markers of Autism Risk in Babies (MARBLES), Programming of Intergenerational Stress Mechanisms (PRISM), New Hampshire Birth Cohort Study (NHBCS), Project Viva, Early Autism Risk Longitudinal Investigation (EARLI)

^b^Cohorts: Atlanta Echo Emory, Conditions Affecting Neurocognitive Development and Learning in Early Childhood (CANDLE), Markers of Autism Risk in Babies (MARBLES), Programming of Intergenerational Stress Mechanisms (PRISM)

^c^n=2510

^d^n=2510

^e^n=3127

^f^n=2366

Supplemental Table 7 - Posterior Inclusion Probabilities from adjusted, hierarchical BKMR with child autism diagnosis in 5 nutrient and 14 nutrient analyses.^a^

|  | **5 nutrient analysis**  **(n = 3280)** | | | **14 nutrient analysis**  **(n = 1596)** | | |
| --- | --- | --- | --- | --- | --- | --- |
| **Nutrient** | **Group** | **Group PIP** | **Conditional PIP** | **Group** | **Group PIP** | **Conditional PIP** |
| Omega 6 fatty acids | Inflammatory | 0.27 | 0.54 | Inflammatory | 0.23 | 0.25 |
| Vitamin D | Inflammatory | 0.27 | 0.21 | Inflammatory | 0.23 | 0.12 |
| Omega 3 fatty acids | Inflammatory | 0.27 | 0.15 | Inflammatory | 0.23 | 0.10 |
| Iron | Inflammatory | 0.27 | 0.10 | Inflammatory | 0.23 | 0.22 |
| Folate | 1-C metab | 0.12 | 1 | 1-C metab | 0.20 | 0.33 |
| Vitamin E |  |  |  | Inflammatory | 0.23 | 0.25 |
| Zinc |  |  |  | Inflammatory | 0.23 | 0.07 |
| Vitamin B6 |  |  |  | 1-C metab | 0.20 | 0.25 |
| Betaine |  |  |  | 1-C metab | 0.20 | 0.14 |
| Vitamin B12 |  |  |  | 1-C metab | 0.20 | 0.13 |
| Methionine |  |  |  | 1-C metab | 0.20 | 0.08 |
| Choline |  |  |  | 1-C metab | 0.20 | 0.06 |
| Vitamin A |  |  |  | Antioxidant | 0.14 | 0.54 |
| Vitamin C |  |  |  | Antioxidant | 0.14 | 0.46 |

1-C metab= one-carbon metabolism pathway; BKMR = Bayesian kernel machine regression

^a^ PIPs are from BKMR models of the association of prenatal nutrient intake with child autism diagnosis, adjusted for maternal age, maternal pre-pregnancy BMI, child sex (male, female), cohort type (enriched familial autism probability, general population), maternal ethnicity/race (Non-Hispanic White, Non-Hispanic Black, Hispanic, Other), maternal education (less than high school, high school/GED, some college/associates degree/trade school, bachelor’s degree, graduate degree), maternal smoking (yes, no), and child year of birth (1998-2004, 2005-2009, 2010-2014, 2015+). Posterior inclusion probabilities (PIPs) are the probabilities for inclusion of a nutrient in the Markov Chain Monte Carlo (MCMC) sampler, representing the relative importance of each exposure in the model. PIPs range from 0-1, with higher values representing higher importance. In the same way, group PIPs describe the relative importance of a group of nutrients. Conditional PIPs describe the relative strength of each nutrient within a group, as given by the percentage of iterations in which the MCMC sampler identifies each nutrient as the most important when the group is included in the model.

Supplemental Table 8 - Association between maternal intake of 5 nutrients during pregnancy and child SRS raw scores using quantile regression at the 50th percentile of SRS scores (n=2,614).^a^

| **Nutrient** | **Quartile of nutrient intake** | **n** | **Median nutrient intake** | **Median SRS raw score** | **Crude** | **Adjusted^b^** |
| --- | --- | --- | --- | --- | --- | --- |
| Vitamin D  (mcg/d) | 1 | 653 | 2.13 | 20.0 | REF | REF |
|  | 2 | 654 | 3.69 | 20.0 | 0.00 (-13.72, 9.72) | -0.76 (-2.84, 1.55) |
|  | 3 | 654 | 5.41 | 21.0 | 1.00 (-12.72, 12.22) | -0.08 (-1.69, 2.07) |
|  | 4 | 653 | 9.16 | 22.0 | 2.00 (-12.22, 12.22) | **2.43 (0.73, 4.56)** |
| Folate  (mcg/d DFE) | 1 | 653 | 308.29 | 22.0 | REF | REF |
|  | 2 | 654 | 419.74 | 21.0 | -1.00 (-4.22, 15.22) | -1.55 (-3.88, 0.45) |
|  | 3 | 654 | 510.66 | 21.0 | -1.00 (-18.22, 15.22) | -1.98 (-4.37, 0.59) |
|  | 4 | 653 | 667.54 | 20.0 | -2.00 (-16.22, 16.22) | -2.53 (-4.60, 0.03) |
| Iron  (mg/d) | 1 | 653 | 11.0 | 22.0 | REF | REF |
|  | 2 | 654 | 13.33 | 20.0 | -2.00 (-10.22, 10.72) | -0.93 (-3.11, 1.40) |
|  | 3 | 654 | 15.22 | 22.0 | 0.00 (-17.22, 12.45) | 0.78 (-1.63, 2.49) |
|  | 4 | 653 | 18.69 | 20.0 | -2.00 (-10.22, 12.22) | 0.15 (-2.08, 2.07) |
| Omega 3 fatty acids  (g/d) | 1 | 653 | 0.95 | 22.0 | REF | REF |
|  | 2 | 654 | 1.24 | 20.0 | -2.00 (-15.72, 11.22) | **-3.20 (-5.00, -1.29)** |
|  | 3 | 654 | 1.49 | 20.0 | -2.00 (-15.45, 14.22) | **-3.47 (-5.41, -0.86)** |
|  | 4 | 653 | 1.97 | 21.0 | -1.00 (-15.22, 6.22) | **-2.95 (-5.04, -1.11)** |
| Omega 6 fatty acids  (g/d) | 1 | 653 | 8.60 | 21.0 | REF | REF |
|  | 2 | 654 | 11.21 | 22.0 | 1.00 (-16.72, 7.22) | -1.63 (-3.51, 0.17) |
|  | 3 | 654 | 13.13 | 21.0 | 0.00 (-16.72, 9.22) | **-2.35 (-4.33, -0.70)** |
|  | 4 | 653 | 16.16 | 20.0 | -1.00 (-20.22, 6.22) | **-3.91 (-5.77, -1.47)** |

SRS – Social Responsiveness Scale

^a^ Quantile regression models of SRS raw score (at the 50^th^ percentile) and dietary nutrient intake quartile. Each nutrient was included in a separate model.

^b^ Adjusted for maternal age, maternal pre-pregnancy BMI, child sex (male, female), cohort type (enriched familial autism probability, general population), maternal ethnicity/race (Non-Hispanic White, Non-Hispanic Black, Hispanic, Other), maternal education (less than high school, high school/GED, some college/associates degree/trade school, bachelor’s degree, graduate degree), maternal smoking (yes, no), and child year of birth (1998-2004, 2005-2009, 2010-2014, 2015+)

Supplemental Table 9- Association between maternal nutrient intake during pregnancy and child SRS raw scores using quantile regression at the 50th percentile of SRS scores in the 14-nutrient subsample (n=952).**^a^**

| **Nutrient** | **Quartile of nutrient intake** | **n** | **Median nutrient intake** | **Median SRS raw score** | **Crude** | **Adjusted^b^** |  |
| --- | --- | --- | --- | --- | --- | --- | --- |
| Vitamin D  (mcg/d) | 1 | 238 | 1.29 | 17.0 | REF | REF |  |
|  | 2 | 238 | 2.77 | 17.0 | 0.00 (-11.70, 11.70) | -2.74 (-6.88, 0.25) |  |
|  | 3 | 238 | 3.97 | 17.0 | 0.00 (-12.70, 12.70) | -2.27 (-6.29, 0.73) |  |
|  | 4 | 238 | 6.55 | 12.5 | -4.00 (NA^c^, 6.70) | **-5.40 (-8.62, -1.78)** |  |
| Folate  (mcg/d DFE) | 1 | 238 | 396.58 | 18.5 | REF | REF |  |
|  | 2 | 238 | 495.62 | 16.0 | -2.00 (-15.70, 8.70) | -1.77 (-4.10, 1.43) |  |
|  | 3 | 238 | 587.55 | 16.0 | -2.00 (-15.70, 7.70) | -2.14 (-4.42, 0.39) |  |
|  | 4 | 238 | 759.44 | 14.0 | -4.00 (-18.70, 9.70) | -0.09 (-2.67, 2.50) |  |
| Iron  (mg/d) | 1 | 238 | 11.06 | 20.0 | REF | REF |  |
|  | 2 | 238 | 13.37 | 15.0 | -5.00 (-14.70, 7.70) | -1.27 (-4.02, 1.06) |  |
|  | 3 | 238 | 14.94 | 17.0 | -3.00 (-12.70, 9.70) | -1.44 (-4.13, 1.18) |  |
|  | 4 | 238 | 17.77 | 13.0 | -7.00 (-18.70, 5.70) | -1.44 (-4.75, 1.52) |  |
| Omega 3 fatty acids  (g/d) | 1 | 238 | 1.19 | 14.0 | REF | REF |  |
|  | 2 | 238 | 1.43 | 16.5 | 2.00 (-12.70, 15.70) | 0.13 (-3.58, 2.52) |  |
|  | 3 | 238 | 1.68 | 15.5 | 2.00 (-13.70, 12.70) | 0.41 (-3.71, 2.50) |  |
|  | 4 | 238 | 2.20 | 18.0 | 4.00 (-6.70, 16.70) | 2.38 (-1.53, 4.82) |  |
| Omega 6 fatty acids  (g/d) | 1 | 238 | 11.14 | 16.5 | REF | REF |  |
|  | 2 | 238 | 13.03 | 17.0 | 1.00 (-14.70, 11.70) | -2.39 (-5.45, 1.16) |  |
|  | 3 | 238 | 14.77 | 15.5 | 0.00 (-14.70, 9.70) | -0.65 (-3.04, 2.80) |  |
|  | 4 | 238 | 17.99 | 16.0 | 0.00 (-11.70, 10.70) | -0.46 (-3.34, 4.08) |  |
| Methionine  (g/d) | 1 | 238 | 1.13 | 20.0 | REF | REF |  |
|  | 2 | 238 | 1.39 | 16.0 | -4.00 (-18.70, 10.70) | -0.96 (-4.42, 2.03) |  |
|  | 3 | 238 | 1.56 | 18.0 | -2.00 (-16.70, 11.70) | -1.92 (-5.90, 0.49) |  |
|  | 4 | 238 | 1.82 | 12.0 | -8.00 (-23.70, 6.70) | -1.73 (-6.22, 0.74) |  |
| Vitamin E  (mg ATE/d) | 1 | 238 | 5.56 | 15.5 | REF | REF |  |
|  | 2 | 238 | 6.89 | 16.5 | 1.00 (-12.70, 16.70) | -1.61 (-4.92, 1.44) |  |
|  | 3 | 238 | 8.07 | 15.0 | 0.00 (-16.70, 12.70) | -2.38 (-6.08, 1.27) |  |
|  | 4 | 238 | 10.14 | 18.0 | 3.00 (-11.70, 11.70) | 1.07 (-2.94, 5.60) |  |
| Vitamin C  (mg/d) | 1 | 238 | 67.94 | 17.5 | REF | REF |  |
|  | 2 | 238 | 116.38 | 18.0 | 1.00 (-17.70, 9.70) | -0.08 (-3.38, 3.02) |  |
|  | 3 | 238 | 152.73 | 13.0 | -4.00 (NA^c^, 9.70) | -2.01 (-5.47, 0.98) |  |
|  | 4 | 238 | 211.20 | 15.0 | -2.00 (-13.70, 10.70) | 0.63 (-2.40, 4.35) |  |
| Betaine  (mg/d) | 1 | 238 | 103.78 | 20.0 | REF | REF |  |
|  | 2 | 238 | 138.80 | 13.5 | -6.00 (-21.70,8.70) | -2.60 (-5.95, 0.24) |  |
|  | 3 | 238 | 174.19 | 14.5 | -5.00 (-20.70, 10.70) | -0.11 (-3.96, 2.67) |  |
|  | 4 | 238 | 251.67 | 18.0 | -2.00 (-16.70, 11.70) | 1.96 (-2.09, 6.00) |  |
| Vitamin A  (mcg RAE/d) | 1 | 238 | 495.55 | 18.5 | REF | REF |  |
|  | 2 | 238 | 712.79 | 18.0 | 0.00 (-13.70, 12.70) | **-2.74 (-5.41, -0.31)** |  |
|  | 3 | 238 | 876.27 | 17.0 | -1.00 (-13.70, 11.70) | **-2.18 (-5.51, -0.06)** |  |
|  | 4 | 238 | 1194.19 | 13.0 | -5.00 (-18.70,7.70) | -2.76 (-6.13,0.39) |  |
| Choline  (mg/d) | 1 | 238 | 214.65 | 18.0 | REF | REF |  |
|  | 2 | 238 | 266.90 | 16.0 | -2.00 (-14.70, 11.70) | -1.63 (-4.51, 1.35) |  |
|  | 3 | 238 | 309.58 | 16.5 | -1.00 (-13.70, 10.70) | -1.30 (-4.67, 2.07) |  |
|  | 4 | 238 | 368.70 | 14.0 | -4.00 (-15.70, 6.70) | -2.33 (-6.85, 0.95) |  |
| Vitamin B6  (mg/d) | 1 | 238 | 1.40 | 18.0 | REF | REF |  |
|  | 2 | 238 | 1.73 | 18.5 | 0.00 (-10.70, 13.70) | -1.20 (-4.34, 1.76) |  |
|  | 3 | 238 | 2.03 | 14.5 | -3.00 (-17.70, 7.70) | **-4.42 (-6.39, -1.07)** |  |
|  | 4 | 238 | 2.49 | 15.0 | -3.00 (-16.70, 7.70) | -1.32 (-4.89, 1.96) |  |
| Vitamin B12  (mcg/d) | 1 | 238 | 3.03 | 19.0 | REF | REF |  |
|  | 2 | 238 | 4.22 | 13.0 | -6.00 (-19.70, 5.70) | **-3.94 (-6.89, -1.37)** |  |
|  | 3 | 238 | 5.18 | 17.5 | -2.00 (-15.70, 13.70) | **-2.80 (-6.43, -0.01)** |  |
|  | 4 | 238 | 6.75 | 16.0 | -3.00 (-16.70, 6.70) | **-3.39 (-6.16, -0.15)** |  |
| Zinc  (mg/d) | 1 | 238 | 8.37 | 19.5 | REF | REF |  |
|  | 2 | 238 | 10.12 | 16.0 | -3.00 (-19.70, 3.70) | -2.07 (-5.66, 0.73) |  |
|  | 3 | 238 | 11.19 | 16.0 | -3.00 (-20.70, 9.70) | -2.10 (-5.39, 1.21) |  |
|  | 4 | 238 | 12.95 | 13.5 | -5.00 (-22.70, 2.70) | **-4.31 (-7.89, -0.51)** |  |

SRS – Social Responsiveness Scale

^a^ Quantile regression models of SRS raw score (at the 50^th^ percentile) and dietary nutrient intake quartile. Each nutrient was included in a separate model.

^b^ Adjusted for maternal age, maternal pre-pregnancy BMI, child sex (male, female), cohort type (enriched familial autism probability, general population), maternal ethnicity/race (Non-Hispanic White, Non-Hispanic Black, Hispanic, Other), maternal education (less than high school, high school/GED, some college/associates degree/trade school, bachelor’s degree, graduate degree), maternal smoking (yes, no), and child year of birth (1998-2004, 2005-2009, 2010-2014, 2015+)

^c^ Model did not provide estimate for lower confidence interval.

Supplemental Table 10 - Association between maternal intake of 5 nutrients during pregnancy and child autism diagnosis using logistic regression (n=3280).^a^

| **Nutrient** | **Quartile of nutrient intake** | **n** | **Autism cases** | **Median nutrient intake** | **Crude** | **Adjusted ^b^** |
| --- | --- | --- | --- | --- | --- | --- |
| Vitamin D  (mcg/d) | 1 | 820 | 37 | 1.94 | REF | REF |
|  | 2 | 820 | 41 | 3.46 | 1.05 (0.88, 1.27) | 1.01 (0.77, 1.32) |
|  | 3 | 820 | 37 | 5.07 | 1.16 (0.93, 1.44) | 1.12 (0.74, 1.7) |
|  | 4 | 820 | 22 | 8.60 | 0.97 (0.77, 1.23) | 0.76 (0.46, 1.26) |
| Folate  (mcg/d DFE) | 1 | 820 | 21 | 318.06 | REF | REF |
|  | 2 | 820 | 27 | 429.37 | 0.93 (0.76, 1.14) | 0.96 (0.53, 1.72) |
|  | 3 | 820 | 49 | 521.48 | **1.21 (1.11, 1.33)** | **1.72 (1.31, 2.28)** |
|  | 4 | 820 | 40 | 692.12 | 1.02 (0.82, 1.28) | 1.47 (0.83, 2.6) |
| Iron  (mg/d) | 1 | 820 | 27 | 11.05 | REF | REF |
|  | 2 | 820 | 34 | 13.33 | 1.00 (0.89, 1.12) | 1.07 (0.79, 1.45) |
|  | 3 | 820 | 44 | 15.21 | 1.07 (0.99, 1.15) | **1.22 (1.03, 1.45)** |
|  | 4 | 820 | 32 | 18.68 | 0.94 (0.81, 1.10) | 1.17 (0.8, 1.72) |
| Omega 3 fatty acids  (g/d) | 1 | 820 | 30 | 0.97 | REF | REF |
|  | 2 | 820 | 45 | 1.28 | 1.17 (0.91, 1.5) | 1.24 (0.73, 2.12) |
|  | 3 | 820 | 30 | 1.53 | 0.90 (0.71, 1.14) | 0.72 (0.45, 1.14) |
|  | 4 | 820 | 32 | 2.03 | 1.06 (0.85, 1.32) | 1.04 (0.61, 1.77) |
| Omega 6 fatty acids  (g/d) | 1 | 820 | 18 | 8.83 | REF | REF |
|  | 2 | 820 | 33 | 11.51 | 1.26 (0.99, 1.61) | 1.63 (0.71, 3.73) |
|  | 3 | 820 | 48 | 13.52 | **1.47 (1.24, 1.74)** | **2.02 (1.13, 3.62)** |
|  | 4 | 820 | 38 | 16.73 | **1.33 (1.08, 1.63)** | 1.79 (0.9, 3.57) |

^a^ Logistic regression models of autism diagnosis and dietary nutrient intake quartile. Each nutrient was included in a separate model.

^b^ Adjusted for maternal age, maternal pre-pregnancy BMI, child sex (male, female), cohort type (enriched familial autism probability, general population), maternal ethnicity/race (Non-Hispanic White, Non-Hispanic Black, Hispanic, Other), maternal education (less than high school, high school/GED, some college/associates degree/trade school, bachelor’s degree, graduate degree), maternal smoking (yes, no), and child year of birth (1998-2004, 2005-2009, 2010-2014, 2015+)

Supplemental Table 11 - Association between maternal intake of 14 nutrients during pregnancy and child autism diagnosis using logistic regression (n= 1596). **^a^**

| **Nutrient** | **Quartile of nutrient intake** | **n** | **Autism Cases** | **Median nutrient intake** | **Crude** | **Adjusted ^b^** |
| --- | --- | --- | --- | --- | --- | --- |
| Vitamin D  (mcg/d) | 1 | 399 | 13 | 1.33 | REF | REF |
|  | 2 | 399 | 21 | 2.77 | 1.12 (0.92, 1.36) | 1.24 (0.81, 1.90) |
|  | 3 | 399 | 24 | 3.93 | **1.26 (1.02, 1.57)** | 1.41 (0.99, 1.99) |
|  | 4 | 399 | 22 | 6.47 | 1.26 (0.8, 1.96) | 1.30 (0.56, 3.03) |
| Folate  (mcg/d DFE) | 1 | 399 | 13 | 389.91 | REF | REF |
|  | 2 | 399 | 24 | 488.33 | 1.43 (0.82, 2.51) | 1.73 (0.68, 4.41) |
|  | 3 | 399 | 23 | 588.09 | **1.36 (1.28, 1.45)** | **1.74 (1.39, 2.18)** |
|  | 4 | 399 | 20 | 767.37 | 1.53 (0.93, 2.53) | 2.28 (0.85, 6.07) |
| Iron  (mg/d) | 1 | 399 | 17 | 11.02 | REF | REF |
|  | 2 | 399 | 25 | 13.27 | **1.16 (1.00, 1.35)** | 1.30 (0.9, 1.89) |
|  | 3 | 399 | 22 | 14.86 | 1.11 (0.91, 1.35) | 1.37 (0.95, 1.98) |
|  | 4 | 399 | 16 | 17.90 | 0.98 (0.71, 1.36) | 1.08 (0.67, 1.74) |
| Omega 3 fatty acids  (g/d) | 1 | 399 | 22 | 1.18 | REF | REF |
|  | 2 | 399 | 21 | 1.43 | 0.85 (0.77, 0.93) | **0.74 (0.65, 0.84)** |
|  | 3 | 399 | 20 | 1.69 | 0.86 (0.64, 1.17) | 0.81 (0.61, 1.07) |
|  | 4 | 399 | 17 | 2.21 | 0.86 (0.54, 1.37) | 0.81 (0.33, 2.00) |
| Omega 6 fatty acids  (g/d) | 1 | 399 | 17 | 11.17 | REF | REF |
|  | 2 | 399 | 24 | 13.11 | 1.22 (0.86, 1.72) | 1.20 (0.69, 2.08) |
|  | 3 | 399 | 23 | 14.92 | 1.17 (0.84, 1.64) | 1.24 (0.66, 2.34) |
|  | 4 | 399 | 16 | 18.17 | 1.07 (0.74, 1.54) | 1.06 (0.55, 2.07) |
| Methionine  (g/d) | 1 | 399 | 22 | 1.13 | REF | REF |
|  | 2 | 399 | 21 | 1.39 | 0.78 (0.51, 1.21) | 0.73 (0.36, 1.52) |
|  | 3 | 399 | 23 | 1.57 | 0.85 (0.55, 1.31) | 0.82 (0.39, 1.71) |
|  | 4 | 399 | 14 | 1.82 | 0.76 (0.53, 1.07) | 0.65 (0.34, 1.23) |
| Vitamin E  (mg ATE/d) | 1 | 399 | 21 | 5.48 | REF | REF |
|  | 2 | 399 | 22 | 6.85 | 0.83 (0.46, 1.5) | 0.68 (0.29, 1.59) |
|  | 3 | 399 | 20 | 8.01 | 0.73 (0.48, 1.11) | 0.64 (0.37, 1.12) |
|  | 4 | 399 | 17 | 10.14 | **0.65 (0.44, 0.98)** | **0.63 (0.42, 0.95)** |
| Vitamin C  (mg/d) | 1 | 399 | 19 | 69.19 | REF | REF |
|  | 2 | 399 | 19 | 113.57 | 0.90 (0.78, 1.04) | 0.85 (0.66, 1.09) |
|  | 3 | 399 | 26 | 149.54 | **1.31 (1.12, 1.53)** | **1.52 (1.34, 1.72)** |
|  | 4 | 399 | 16 | 216.83 | 1.07 (0.92, 1.25) | 1.17 (0.94, 1.45) |
| Betaine  (mg/d) | 1 | 399 | 24 | 102.77 | REF | REF |
|  | 2 | 399 | 18 | 137.64 | **0.67 (0.47, 0.96)** | **0.60 (0.37, 0.98)** |
|  | 3 | 399 | 18 | 172.69 | 0.72 (0.47, 1.11) | 0.75 (0.40, 1.41) |
|  | 4 | 399 | 20 | 255.99 | 0.87 (0.62, 1.23) | 1.04 (0.58, 1.85) |
| Vitamin A  (mcg RAE/d) | 1 | 399 | 23 | 473.50 | REF | REF |
|  | 2 | 399 | 19 | 695.88 | **0.65 (0.50, 0.85)** | **0.55 (0.37, 0.8)** |
|  | 3 | 399 | 20 | 862.80 | **0.74 (0.62, 0.89)** | **0.64 (0.51, 0.82)** |
|  | 4 | 399 | 18 | 1174.15 | 0.83 (0.63, 1.08) | 0.82 (0.48, 1.39) |
| Choline  (mg/d) | 1 | 399 | 21 | 212.88 | REF | REF |
|  | 2 | 399 | 22 | 265.32 | 0.89 (0.51, 1.55) | 0.80 (0.37, 1.71) |
|  | 3 | 399 | 13 | 308.82 | **0.59 (0.50, 0.69)** | **0.45 (0.31, 0.65)** |
|  | 4 | 399 | 24 | 374.77 | 1.09 (0.68, 1.73) | 1.16 (0.61, 2.20) |
| Vitamin B6  (mg/d) | 1 | 399 | 17 | 1.38 | REF | REF |
|  | 2 | 399 | 23 | 1.73 | 1.06 (0.71, 1.58) | 1.07 (0.72, 1.57) |
|  | 3 | 399 | 21 | 2.02 | 1.04 (0.56, 1.91) | 1.03 (0.42, 2.55) |
|  | 4 | 399 | 19 | 2.54 | 1.05 (0.66, 1.69) | 1.34 (0.62, 2.9) |
| Vitamin B12  (mcg/d) | 1 | 399 | 11 | 2.94 | REF | REF |
|  | 2 | 399 | 23 | 4.20 | **1.54 (1.18, 2.02)** | **2.06 (1.47, 2.88)** |
|  | 3 | 399 | 27 | 5.11 | **1.60 (1.15, 2.23)** | **2.11 (1.38, 3.25)** |
|  | 4 | 399 | 19 | 6.78 | 1.41 (0.91, 2.19) | 1.78 (0.85, 3.73) |
| Zinc  (mg/d) | 1 | 399 | 17 | 8.29 | REF | REF |
|  | 2 | 399 | 23 | 10.08 | 1.03 (0.96, 1.11) | 1.08 (0.91, 1.28) |
|  | 3 | 399 | 24 | 11.12 | 1.08 (0.78, 1.50) | 1.24 (0.75, 2.08) |
|  | 4 | 399 | 16 | 13.02 | 0.89 (0.79, 1.00) | 0.93 (0.74, 1.18) |

^a^ Logistic regression models of autism diagnosis and dietary nutrient intake quartile. Each nutrient was included in a separate model.

^b^ Adjusted for maternal age, maternal pre-pregnancy BMI, child sex (male, female), cohort type (enriched familial autism probability, general population), maternal ethnicity/race (Non-Hispanic White, Non-Hispanic Black, Hispanic, Other), maternal education (less than high school, high school/GED, some college/associates degree/trade school, bachelor’s degree, graduate degree), maternal smoking (yes, no), and child year of birth (1998-2004, 2005-2009, 2010-2014, 2015+)

Supplemental Table 12 – Intake from supplements among nutrients included in sensitivity analysis of total nutrient intake with SRS scores (n=952).

|  | **Mean (SD)** |
| --- | --- |
| Supplemental Folic Acid (mcg/d DFE) | 841.04 (457.61) |
| Supplemental Vitamin D (mcg/d) | 4.80 (3.25) |
| Supplemental Omega 3 fatty acids (g/d) | 0.07 (0.17) |
| Supplemental Iron (mg/d) | 28.47 (24.04) |
| Supplemental Zinc (mg/d) | 9.71 (6.97) |
| Supplemental Vitamin B12 (mcg/d) | 2.65 (2.35) |
| Supplemental Vitamin B6 (mg/d) | 1.65 (1.05) |
| Supplemental Vitamin A (mcg RAE/d) | 1265.00 (716.20) |
| Supplemental Vitamin C (mg/d) | 90.66 (131.99) |
| Supplemental Vitamin E (mg ATE/d) | 6.83 (20.80) |

Supplemental Table 13 – Association between maternal intake of 5 nutrients during pregnancy and child SRS raw scores using quantile regression at the 50th percentile, with additional adjustment for covariates.

| **Nutrient** | **Quartile of nutrient intake** | **Basic model^a^**  (n=2614) | **+ food frequency questionnaire type**  (n=2,614) | **+ prenatal vitamins (ever)^b^**  (n=2,614) | **+ prenatal vitamins (ever)^c^**  (n=2,614) | **+ parity^b^**  (n=2614) | **+ pregnancy complications (hypertension, diabetes, preeclampsia)^b^** (n=2,614) | **+preterm birth^b^**  (n=2,614) | **+ breastfeeding (ever)^c^**  (n=2,614) | **+ income^c^**  (n=2,614) |
| --- | --- | --- | --- | --- | --- | --- | --- | --- | --- | --- |
| Vitamin D | 1 | REF | REF | REF | REF | REF | REF | REF | REF | REF |
|  | 2 | -0.76 (-2.84, 1.55) | -1.66 (-3.47, 0.15) | -0.61 (-2.80, 1.50) | -0.47 (-2.84), 1.91) | -0.62 (-2.77, 1.50) | -0.46 (-2.77, 1.51) | -0.58 (-2.93, 1.32) | -1.66 (-3.73, 0.41) | -0.90 (-3.02, 1.22) |
|  | 3 | -0.08 (-1.69, 2.07) | -1.60 (-3.55, 0.34) | 0.11 (-1.68, 1.91) | 0.46 (-1.53, 2.45) | 0.03 (-1.36, 1.68) | 0.21 (-1.88, 2.02) | 0.32 (-1.55, 1.86) | -0.97 (-3.03, 1.08) | -0.33 (-2.29, 1.63) |
|  | 4 | **2.43 (0.73, 4.56)** | 0.09 (-1.88, 2.05) | **2.63 (0.50, 4.53)** | **2.39 (0.43, 4.36)** | **2.60 (0.65, 4.32)** | **2.72 (0.72, 4.48)** | **2.63 (0.45, 4.44)** | 1.38 (-0.45, 3.21) | **2.23 (0.33, 4.14)** |
| Folate | 1 | REF | REF | REF | REF | REF | REF | REF | REF | REF |
|  | 2 | -1.55 (-3.88, 0.45) | **-2.02 (-3.82, -0.22)** | -1.88 (-3.96, 0.55) | -1.34 (-3.42, 0.73) | -1.57 (-3.70, 0.44) | -1.39 (-3.99, 0.57) | -1.29 (-3.95, 0.71) | -1.72 (-3.89, 0.45) | -1.12 (-3.20, 0.96) |
|  | 3 | -1.98 (-4.37, 0.59) | -0.90 (-2.94, 1.14) | -2.33 (-4.35, 0.50) | -1.62 (-3.88, 0.65) | -2.15 (-4.28, 0.54) | -1.88 (-4.40, 0.54) | -1.70 (-4.27, 0.38) | -1.99 (-4.34, 0.37) | -1.68 (-3.79, 0.42) |
|  | 4 | -2.53 (-4.60, 0.03) | 0.30 (-1.86, 2.46) | **-2.96 (-4.73, -0.32)** | **-2.42 (-4.73, -0.10)** | -2.56 (-4.63, 0.07) | -2.47 (-4.93, 0.13) | **-2.09 (-4.59, -0.15)** | **-2.90 (-5.08, -0.73)** | -1.97 (-4.16, 0.23) |
| Iron | 1 | REF | REF | REF | REF | REF | REF | REF | REF | REF |
|  | 2 | -0.93 (-3.11, 1.40) | -0.36 (-2.30, 1.58) | -1.04 (-2.94, 1.48) | -0.98 (-3.02, 1.06) | -0.73 (-2.76, 1.30) | -0.68 (-3.24, 1.43) | -1.02 (-2.91, 1.29) | -1.68 (-3.69, 0.33) | -0.86 (-2.95, 1.23) |
|  | 3 | 0.78 (-1.63, 2.49) | 0.64 (-1.30, 2.57) | 0.22 (-1.82, 2.38) | 0.16 (-1.65, 1.97) | 0.63 (-1.66, 2.57) | 0.81 (-1.54, 2.40) | 0.38 (-1.67, 2.48) | -0.13 (-2.09, 1.83) | 0.35 (-1.58, 2.27) |
|  | 4 | 0.15 (-2.08, 2.07) | 0.07 (-2.04, 2.18) | 0.06 (-2.04, 2.01) | -0.06 (-2.01, 1.89) | 0.20 (-1.99, 2.11) | 0.30 (-2.22, 2.22) | -0.16 (-2.32, 1.72) | -1.10 (-2.98, 0.79) | 0.18 (-1.89, 2.25) |
| Omega 3 fatty acids | 1 | REF | REF | REF | REF | REF | REF | REF | REF | REF |
|  | 2 | **-3.20 (-5.00, -1.29)** | **-2.02 (-3.82, -0.22)** | **-3.18 (-5.09, -1.45)** | **-2.86 (-4.63, -1.08)** | **-3.22 (-4.85 ,-1.27)** | **-3.17 (-5.12, -1.28)** | **-3.09 (-4.98, -1.14)** | **-2.86 (-4.89, -0.83)** | **-3.01 (-4.96, -1.06)** |
|  | 3 | **-3.47 (-5.41, -0.86)** | -0.90 (-2.94, 1.14) | **-3.51 (-5.35, -1.13)** | **-3.10 (-4.99, -1.22)** | **-3.46 (-5.27, -1.34)** | **-3.30 (-5.40, -0.95)** | **-3.45 (-5.46, -1.12)** | **-2.96 (-5.18, -0.75)** | **-2.35 (-4.35, -0.35)** |
|  | 4 | **-2.95 (-5.04, -1.11)** | 0.30 (-1.86, 2.46) | **-2.91 (-4.75, -1.01)** | **-2.79 (-4.88, -0.70)** | **-3.06 (-4.92, -1.30)** | **-2.94 (-4.99, -1.09)** | **-2.86 (-4.96, -0.98)** | **-2.70 (-4.79, -0.61)** | **-2.32 (-4.50, -0.15)** |
| Omega 6 fatty acids | 1 | REF | REF | REF | REF | REF | REF | REF | REF | REF |
|  | 2 | -1.63 (-3.51, 0.17) | -0.41 (-2.43, 1.61) | **-1.50 (-3.46, -0.05)** | -1.23 (-3.09, 0.62) | -1.59 (-3.38, 0.29) | -1.67 (-3.54, 0.17) | -1.49 (-3.56, 0.29) | -1.71 (-3.79, 0.37) | -0.76 (-2.78, 1.26) |
|  | 3 | **-2.35 (-4.33,-0.70)** | -0.20 (-2.42, 2.02) | **-2.42 (-4.56, -0.65)** | **-2.38 (-4.40, -0.35)** | **-2.56 (-4.30, -0.40)** | **-2.28 (-4.48, -0.58)** | **-2.45 (-4.44, -0.57)** | -2.16 (-4.39, 0.06) | -1.44 (-3.42, 0.53) |
|  | 4 | **-3.91 (-5.77,-1.47)** | 0.57 (-1.57, 2.70) | **-3.68 (-5.99, -1.50)** | **-3.37 (-5.76, -0.98)** | **-3.78 (-5.86, -1.42)** | **-3.82 (-6.07, -1.53)** | **-3.65 (-6.09, -1.08)** | **-2.73 (-5.09, -0.37)** | **-2.74 (-4.94, -0.55)** |

^a^ Adjusted for maternal age, maternal pre-pregnancy BMI (<18.5, 18.5-24.9, 25-29.9, >=30), child sex (male, female), cohort type (enriched familial autism probability, general population), maternal ethnicity/race (Non-Hispanic White, Non-Hispanic Black, Hispanic, Other), maternal education (less than high school, high school/GED, some college/associates degree/trade school, bachelor’s degree, graduate degree), maternal smoking (yes, no), and child year of birth (1998-2004, 2005-2009, 2010-2014, 2015+)

^b^ Missing values imputed with multiple imputation

^c^ Missing values treated as separate category

Supplemental Table 14 – Association between maternal nutrient intake during pregnancy and child SRS raw scores in the 5-nutrient sample using adjusted quantile regression (SRS at 50^th^ percentile), stratified by food frequency questionnaire (FFQ).^a^

|  |  |  | **Full sample**  **(n=2614)** | | **Block FFQ**  **(n=952)** | | **Harvard FFQ**  **(n=1571)** | |
| --- | --- | --- | --- | --- | --- | --- | --- | --- |
| **Nutrient** | **Quartile of nutrient intake** | **n** | **Median nutrient intake** | **Estimate (CIs)** | **Median nutrient intake** | **Estimate (CIs)** | **Median nutrient intake** | **Estimate (CIs)** |
| Vitamin D (mcg/d) | 1 | 653 | 2.13 | REF | 1.99 | REF | 2.34 | REF |
|  | 2 | 654 | 3.69 | -0.76 (-2.84, 1.55) | 3.65 | -3.30 (-5.48, -0.94) | 3.73 | -0.02 (-2.13, 3.30) |
|  | 3 | 654 | 5.41 | -0.08 (-1.69, 2.07) | 5.31 | -3.63 (-6.74, -0.67) | 5.49 | 0.06 (-1.72, 3.00) |
|  | 4 | 653 | 9.16 | 2.43 (0.73, 4.56) | 8.31 | -3.31 (-6.97, 0.98) | 9.38 | 1.18 (-0.83, 3.81) |
| Folate (mcg/d DFE) | 1 | 653 | 308.29 | REF | 327.12 | REF | 305.73 | REF |
|  | 2 | 654 | 419.74 | -1.55 (-3.88, 0.45) | 424.22 | -0.76 (-4.03, 4.17) | 416.55 | -1.49 (-4.01, 0.78) |
|  | 3 | 654 | 510.66 | -1.98 (-4.37, 0.59) | 514.23 | -1.40 (-5.70, 1.54) | 508.20 | -1.41 (-3.69, 1.18) |
|  | 4 | 653 | 667.54 | -2.53 (-4.60, 0.03) | 679.84 | -0.72 (-4.40, 2.46) | 650.52 | -0.73 (-3.65, 1.34) |
| Iron (mg/d) | 1 | 653 | 11.0 | REF | 11.05 | REF | 10.99 | REF |
|  | 2 | 654 | 13.33 | -0.93 (-3.11, 1.40) | 13.37 | -1.10 (-3.73, 1.54) | 13.27 | -0.06 (-2.03, 3.05) |
|  | 3 | 654 | 15.22 | 0.78 (-1.63, 2.49) | 15.10 | -1.23 (-3.96, 2.49) | 15.32 | 1.07 (-0.82, 3.56) |
|  | 4 | 653 | 18.69 | 0.15 (-2.08, 2.07) | 18.15 | -1.62 (-4.87, 1.25) | 19.15 | 0.75 (-1.38, 3.28) |
| Omega 3 fatty acids (g/d) | 1 | 653 | 0.95 | REF | 0.99 | REF | 0.94 | REF |
|  | 2 | 654 | 1.24 | -3.20 (-5.00, -1.29) | 1.26 | -2.88 (-7.08, 2.27) | 1.23 | -1.44 (-3.64, 0.30) |
|  | 3 | 654 | 1.49 | -3.47 (-5.41, -0.86) | 1.49 | -3.28 (-6.98, 1.85) | 1.50 | 0.29 (-2.05, 1.87) |
|  | 4 | 653 | 1.97 | -2.95 (-5.04, -1.11) | 2.00 | -1.35 (-5.46, 3.88) | 1.95 | 0.80 (-2.13, 3.06) |
| Omega 6 fatty acids (g/d) | 1 | 653 | 8.60 | REF | 9.12 | REF | 8.54 | REF |
|  | 2 | 654 | 11.21 | -1.63 (-3.51, 0.17) | 11.45 | 2.49 (-2.22, 6.70) | 11.12 | -1.36 (-3.21, 0.48) |
|  | 3 | 654 | 13.13 | -2.35 (-4.33, -0.70) | 13.17 | 0.99 (-3.10, 4.11) | 13.10 | -0.52 (-3.35, 1.70) |
|  | 4 | 653 | 16.16 | -3.91 (-5.77, -1.47) | 16.34 | 1.22 (-3.04, 4.17) | 16.00 | 0.07 (-2.66, 2.62) |

^a^ Adjusted for maternal age, maternal pre-pregnancy BMI, child sex (male, female), cohort type (enriched familial autism probability, general population), maternal ethnicity/race (Non-Hispanic White, Non-Hispanic Black, Hispanic, Other), maternal education (less than high school, high school/GED, some college/associates degree/trade school, bachelor’s degree, graduate degree), maternal smoking (yes, no), and child year of birth (1998-2004, 2005-2009, 2010-2014, 2015+)

Supplemental Table 15 – Association between maternal nutrient intake during pregnancy and child autism diagnosis in the 5-nutrient sample using adjusted logistic regression, stratified by food frequency questionnaire (FFQ). ^a^

|  |  |  |  | **Full sample**  **(n=3280)** | | **Block FFQ**  **(n=** **1596)** | | **Harvard FFQ**  **(n=1584)**^b^ | |
| --- | --- | --- | --- | --- | --- | --- | --- | --- | --- |
| **Nutrient** | **Quartile of nutrient intake** | **n** | **Autism cases** | **Median nutrient intake** | **Estimate (CIs)** | **Median nutrient intake** | **Estimate (CIs)** | **Median nutrient intake** | **Estimate (CIs)** |
| Vitamin D | 1 | 820 | 37 | 1.94 | REF | 1.80 | REF | 2.23 | REF |
|  | 2 | 820 | 41 | 3.46 | 1.01 (0.77, 1.32) | 3.40 | 1.03 (0.77, 1.37) | 3.50 | 0.57 (0.18, 1.85) |
|  | 3 | 820 | 37 | 5.07 | 1.12 (0.74, 1.70) | 5.04 | 1.43 (0.96, 2.13) | 5.14 | 0.57 (0.19, 1.71) |
|  | 4 | 820 | 22 | 8.60 | 0.76 (0.46, 1.26) | 7.92 | 0.94 (0.39, 2.24) | 8.91 | 0.35 (0.11, 1.13) |
| Folate | 1 | 820 | 21 | 318.06 | REF | 340.27 | REF | 11.04 | REF |
|  | 2 | 820 | 27 | 429.37 | 0.96 (0.53, 1.72) | 431.44 | 0.99 (0.52, 1.89) | 13.27 | 0.57 (0.18, 1.78) |
|  | 3 | 820 | 49 | 521.48 | **1.72 (1.31, 2.28)** | 521.83 | **2.04 (1.48, 2.81)** | 15.32 | 1.47 (0.49, 4.39) |
|  | 4 | 820 | 40 | 692.12 | 1.47 (0.83, 2.60) | 697.89 | **1.91 (1.19, 3.06)** | 19.07 | 1.29 (0.27, 6.17) |
| Iron | 1 | 820 | 27 | 11.05 | REF | 11.05 | REF | 309.26 | REF |
|  | 2 | 820 | 34 | 13.33 | 1.07 (0.79, 1.45) | 13.36 | 1.13 (0.87, 1.47) | 426.37 | 0.81 (0.22, 2.94) |
|  | 3 | 820 | 44 | 15.21 | **1.22 (1.03, 1.45)** | 15.12 | 1.16 (0.91, 1.49) | 517.72 | 1.34 (0.42, 4.26) |
|  | 4 | 820 | 32 | 18.68 | 1.17 (0.80, 1.72) | 18.37 | 1.04 (0.60, 1.82) | 667.48 | 0.95 (0.29, 3.14) |
| Omega 3 fatty acids | 1 | 820 | 30 | 0.97 | REF | 1.01 | REF | 0.96 | REF |
|  | 2 | 820 | 45 | 1.28 | 1.24 (0.73, 2.12) | 1.29 | 0.82 (0.38, 1.76) | 1.27 | 0.99 (0.37, 2.64) |
|  | 3 | 820 | 30 | 1.53 | 0.72 (0.45, 1.14) | 1.53 | **0.48 (0.31, 0.75)** | 1.52 | 0.54 (0.14, 2.01) |
|  | 4 | 820 | 32 | 2.03 | 1.04 (0.61, 1.77) | 2.05 | **0.65 (0.48, 0.89)** | 2.02 | 1.19 (0.38, 3.75) |
| Omega 6 fatty acids | 1 | 820 | 18 | 8.83 | REF | 9.35 | REF | 8.66 | REF |
|  | 2 | 820 | 33 | 11.51 | 1.63 (0.71, 3.73) | 11.69 | 1.47 (0.33, 6.44) | 11.35 | 0.83 (0.28, 2.47) |
|  | 3 | 820 | 48 | 13.52 | **2.02 (1.13, 3.62)** | 13.58 | 1.59 (0.38, 6.62) | 13.42 | 2.35 (0.85, 6.50) |
|  | 4 | 820 | 38 | 16.73 | 1.79 (0.90, 3.57) | 16.77 | 1.38 (0.50, 3.82) | 16.72 | 1.41 (0.36, 5.52) |

^a^Adjusted for maternal age, maternal pre-pregnancy BMI, child sex (male, female), maternal ethnicity/race (Non-Hispanic White, Non-Hispanic Black, Hispanic, Other), maternal education (less than high school, high school/GED, some college/associates degree/trade school, bachelor’s degree, graduate degree), maternal smoking (yes, no), and child year of birth (1998-2004, 2005-2009, 2010-2014, 2015+).

^b^Model did not include adjustment for education due to limited variability in this subset.

Supplemental Figure 1 – Participant selection flow chart for this analysis of nutrient and autism outcomes in the ECHO cohort.

All ECHO cohorts

(68 cohorts, 71,508 pregnancies, 66,612 children)

Matched mother-child dyads

(68 cohorts, 62,626 pregnancies)

Any prenatal dietary data

(17 cohorts, 10,484 pregnancies)

Child autism outcome data

(17 cohorts, 6,429 children)

Excluding twins and randomly selecting one child per family

(17 cohorts, 6,243 children)

Primary 5 nutrient analysis

(7 cohorts, 3,502 children)

**Autism diagnosis**

**(7 cohorts, 3,280 children, 137 cases)**

**SRS score**

**(7 cohorts, 2,614 children)**

Secondary 14 nutrient analysis

(4 cohorts, 1,619 children)

**Autism diagnosis**

**(4 cohorts, 1,596 children, 80 cases)**

**SRS score**

**(4 cohorts, 952 pregnancies)**

Supplemental Figure 2 – Heat maps of Spearman correlations of nutrients in the a) 5-nutrient sample and b) 14-nutrient sample of ECHO participants.

**A**


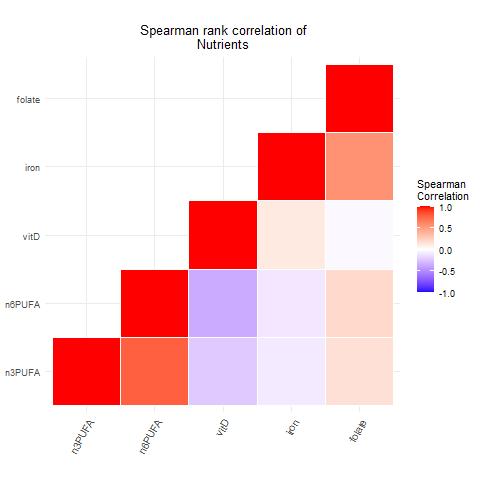


**B**


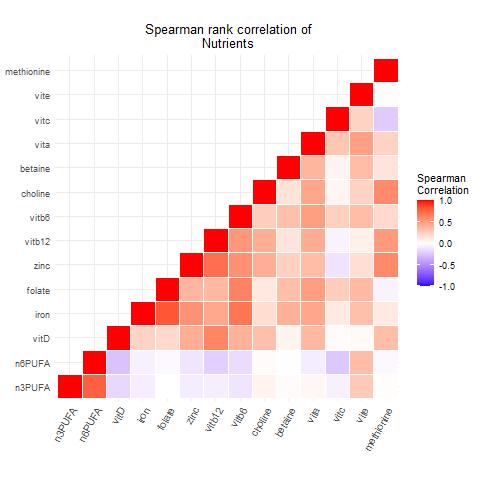


Supplemental Figure 3 - Adjusted associations between prenatal intake of 5 nutrients and child autism diagnosis using hierarchical BKMR (n=3280).

**A B**

**
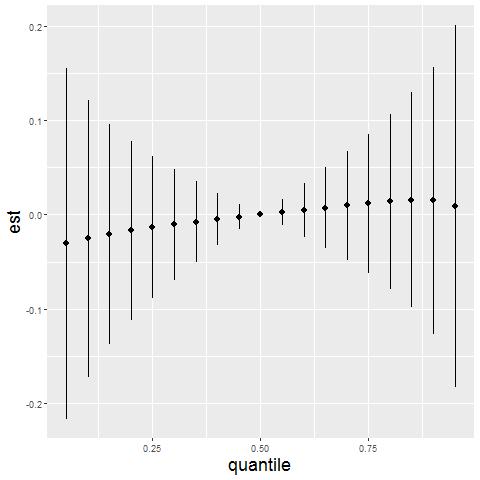

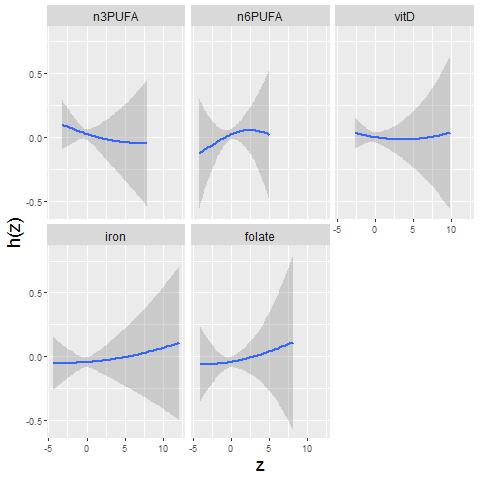
**

***Figure legend:*** Results of probit Bayesian kernel machine regression (BKMR) analyses in ECHO including energy-adjusted nutrient intake for omega 3 fatty acids, omega 6 fatty acids, iron, vitamin D, folate, adjusted for maternal age, maternal pre-pregnancy BMI, child sex (male, female), cohort type (enriched familial autism probability, general population), maternal ethnicity/race (Non-Hispanic White, Non-Hispanic Black, Hispanic, Other), maternal education (less than high school, high school/GED, some college/associates degree/trade school, bachelor’s degree, graduate degree), maternal smoking (yes, no), and child year of birth (1998-2004, 2005-2009, 2010-2014, 2015+). Plots show: (A) the overall mixture effect, or the relationship between the nutrient mixture and child autism diagnosis. (B) Individual nutrient associations with autism diagnosis, holding all other nutrients at their 50^th^ percentile. The set of nutrients included here was selected as described in text based on a priori interest for neurodevelopmental relevance and grouped by shared pathway.

Supplemental Figure 4 - Adjusted associations between prenatal intake of 14 nutrients and child autism diagnosis using hierarchical BKMR (n=1596).

**A B**

**
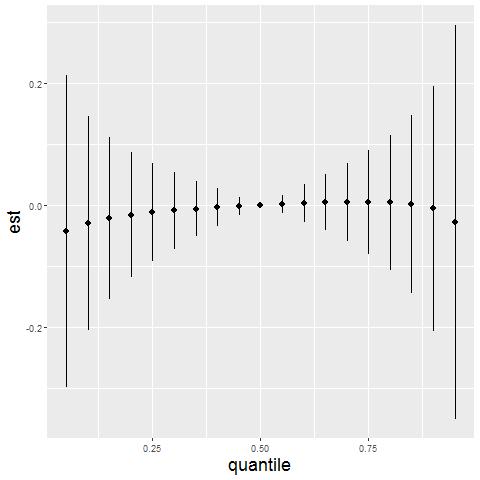

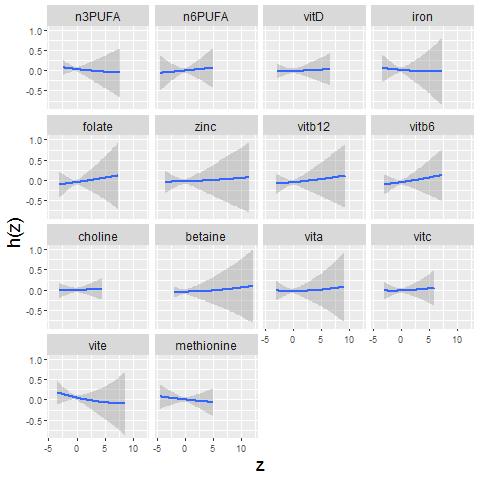
**

***Figure legend:*** Results of probit Bayesian kernel machine regression (BKMR) analyses in ECHO including energy-adjusted nutrient intake for omega 3 and omega 6 fatty acids, iron, vitamin D, folate, zinc, vitamin B12, vitamin B6, choline, betaine, vitamin A, vitamin C, vitamin E, and methionine, adjusted for maternal age, maternal pre-pregnancy BMI, child sex (male, female), cohort type (enriched familial autism probability, general population), maternal ethnicity/race (Non-Hispanic White, Non-Hispanic Black, Hispanic, Other), maternal education (less than high school, high school/GED, some college/associates degree/trade school, bachelor’s degree, graduate degree), maternal smoking (yes, no), and child year of birth (1998-2004, 2005-2009, 2010-2014, 2015+). Plots show: (A) the overall mixture effect, or the relationship between the nutrient mixture and child autism diagnosis. (B) Individual nutrient associations with autism diagnosis, holding all other nutrients at their 50^th^ percentile. The set of nutrients included here was selected as described in text based on a priori interest for neurodevelopmental relevance and grouped by shared pathway.

Supplemental Figure 5 - Adjusted associations between prenatal intake of 5 nutrients and child SRS scores using hierarchical BKMR, limited to the subsample with complete data on 14 nutrients (n=952).

**A B**

**
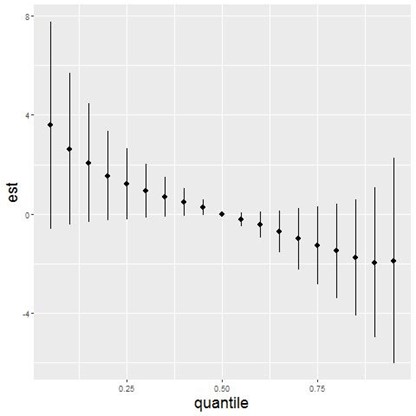

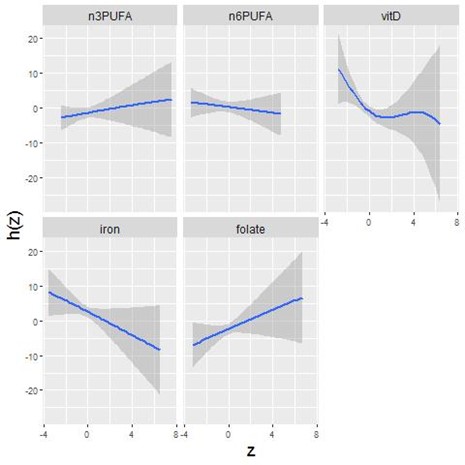
**

***Figure legend:*** Results of Bayesian kernel machine regression (BKMR) analyses in ECHO including energy-adjusted nutrient intake for omega 3 and omega 6 fatty acids, iron, vitamin D, folate, adjusted for maternal age, maternal pre-pregnancy BMI, child sex (male, female), cohort type (enriched familial autism probability, general population), maternal ethnicity/race (Non-Hispanic White, Non-Hispanic Black, Hispanic, Other), maternal education (less than high school, high school/GED, some college/associates degree/trade school, bachelor’s degree, graduate degree), maternal smoking (yes, no), and child year of birth (1998-2004, 2005-2009, 2010-2014, 2015+). Plots show: (A) the overall mixture effect, or the relationship between the nutrient mixture and child SRS scores. (B) Individual nutrient associations with SRS scores, holding all other nutrients at their 50^th^ percentile. The set of nutrients included here was selected as described in text based on a priori interest for neurodevelopmental relevance and grouped by shared pathway.

Supplemental Figure 6 – Adjusted associations between prenatal intake of 5 nutrients (from diet and supplements) and child SRS scores using hierarchical BKMR (n=952).

**A B**

**
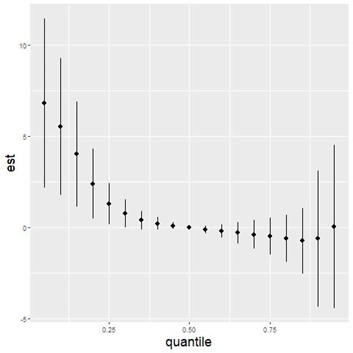

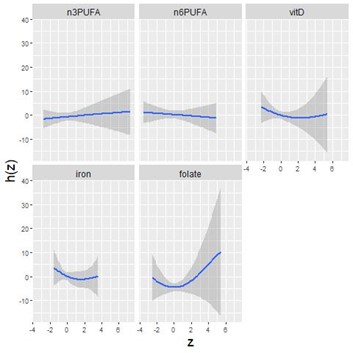
**

***Figure legend:*** Results of Bayesian kernel machine regression (BKMR) analyses in ECHO including energy-adjusted nutrient intake from diet and supplements for omega 3 fatty acids, omega 6 fatty acids (dietary only), iron, vitamin D, and folate, adjusted for maternal age, maternal pre-pregnancy BMI, child sex (male, female), cohort type (enriched familial autism probability, general population), maternal ethnicity/race (non-Hispanic white, non-Hispanic Black, Hispanic, Other), maternal education (less than high school, high school/GED, some college/associates degree/trade school, bachelor’s degree, graduate degree), maternal smoking (yes, no), and child year of birth (1998-2004, 2005-2009, 2010-2014, 2015+). Plots show: (A) the overall mixture effect, or the relationship between the nutrient mixture and child SRS scores. (B) Individual nutrient associations with SRS scores, holding all other nutrients at their 50^th^ percentile. The primary set of nutrients included here was selected as described in text based on a priori interest for neurodevelopmental relevance and grouped by shared pathway.

Supplemental Figure 7 – Leave-one-out analyses examining the influence of excluding individual ECHO cohorts on the association between maternal intake of 5 nutrients during pregnancy and child SRS raw scores using quantile regression at the 50th percentile of SRS scores.

**A B**

**
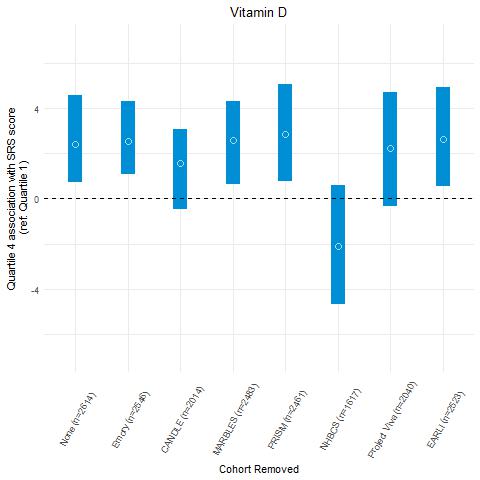

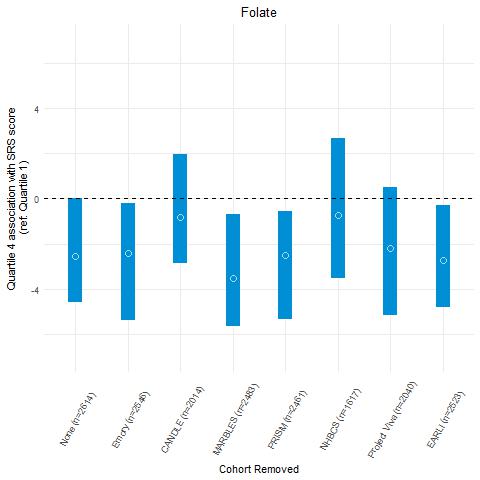
**

**C D**

**
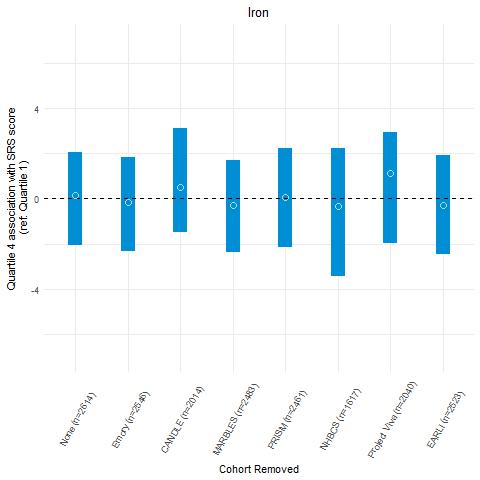

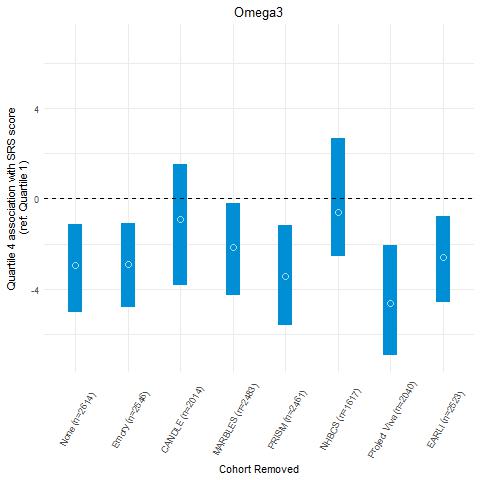
**

**E**

***
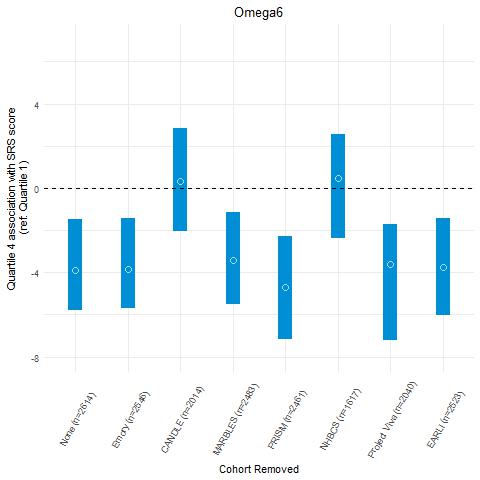
***

***Figure legend:*** Plots show estimate and confidence intervals from quantile regression models examining the association of nutrient intake at the highest quartile (vs the lowest) with SRS scores, modeled at the 50^th^ quantile of SRS scores. The far-left bar in each plot shows the association in the full analytic sample of 7 ECHO cohorts; each remaining bar shows the association when a given cohort is removed from analysis. Estimates are provided for each nutrient separately: a) vitamin D, b) folate, c) iron, d) omega 3 fatty acids, e) omega 6 fatty acids.

Supplemental Figure 8 - Adjusted associations between prenatal intake of 5 nutrients and child SRS scores using hierarchical BKMR, with additional adjustment for food frequency questionnaire type (n= 2614).

**A B**

**
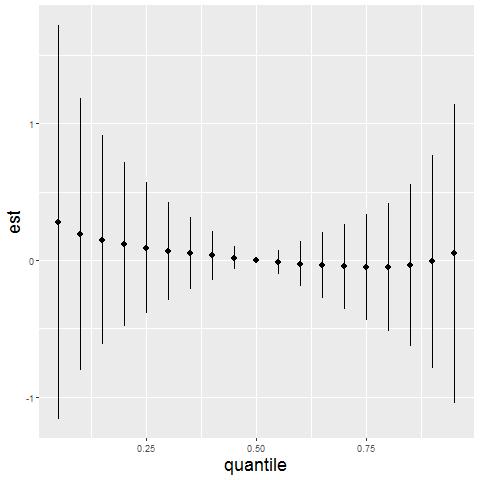

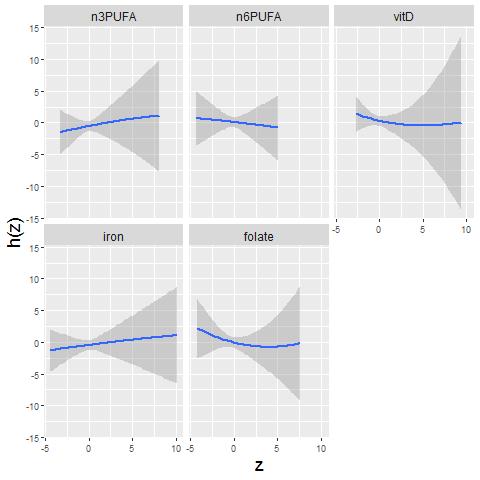
**

***Figure legend:*** Results of Bayesian kernel machine regression (BKMR) analyses in ECHO including energy-adjusted nutrient intake for omega 3 and omega 6 fatty acids, iron, vitamin D, folate, adjusted for maternal age, maternal pre-pregnancy BMI, child sex (male, female), cohort type (enriched familial autism probability, general population), maternal ethnicity/race (Non-Hispanic White, Non-Hispanic Black, Hispanic, Other), maternal education (less than high school, high school/GED, some college/associates degree/trade school, bachelor’s degree, graduate degree), maternal smoking (yes, no), child year of birth (1998-2004, 2005-2009, 2010-2014, 2015+), and food frequency questionnaire type (Block, Willett, or Diet History Questionnaire). Plots show: (A) the overall mixture effect, or the relationship between the nutrient mixture and child SRS scores. (B) Individual nutrient associations with SRS scores, holding all other nutrients at their 50^th^ percentile. The set of nutrients included here was selected as described in text based on a priori interest for neurodevelopmental relevance and grouped by shared pathway.

Appendix A. ECHO Collaborators

| **First Name and Middle Initial(s)** | **Last Name** | **Suffix (e.g., Jr, III)** | **Academic Degrees** | **Department** | **Institution** | **Location (city, state/province, country)** | **Role or Contribution** | **ECHO Cohort Study Site or Core Name and Grant Number** | **Email Address** |
| --- | --- | --- | --- | --- | --- | --- | --- | --- | --- |
| P Brian | Smith |  | MD, MPH, MHS | Division of Neonatology, Department of Pediatrics | Duke Clinical Research Institute, Duke University School of Medicine | Durham, North Carolina, USA | ECHO Coordinating Center Principal Investigator | U2COD023375 (Coordinating Center) | [brian.smith@duke.edu](mailto:brian.smith@duke.edu) |
| L Kristin | Newby |  | MD, MHS | Division of Cardiology, Department of Medicine | Duke Clinical Research Institute, Duke University School of Medicine | Durham, North Carolina, USA | ECHO Coordinating Center Principal Investigator | U2COD023375 (Coordinating Center) | [kristin.newby@duke.edu](mailto:kristin.newby@duke.edu) |
| Linda | Adair |  | PhD | Department of Nutrition | Gillings School of Global Public Health, University of North Carolina at Chapel Hill | Chapel Hill, North Carolina, USA | ECHO Coordinating Center Principal Investigator | U2COD023375 (Coordinating Center) | [linda_adair@unc.edu](mailto:linda_adair@unc.edu) |
| Lisa P. | Jacobson |  | ScD | Department of Epidemiology | Johns Hopkins University, Bloomberg School of Public Health | Baltimore, Maryland, USA | ECHO Data Analysis Center Principal Investigator | U24OD023382 (Data Analysis Center) | ljacobs1@jhu.edu |
| Diane | Catellier |  | DrPH | N/A | Research Triangle Institute | Research Triangle Park, North Carolina, USA | ECHO Data Analysis Center Principal Investigator | U24OD023382 (Data Analysis Center) | dcatellier@rti.org |
| Monica | McGrath |  | ScD | Department of Epidemiology | Johns Hopkins University, Bloomberg School of Public Health | Baltimore, Maryland, USA | ECHO Johns Hopkins University Data Analysis Center Director Co-Investigator | U24OD023382 (Data Analysis Center) | mmcgrat4@jhu.edu |
| Christian | Douglas |  | DrPH | N/A | Research Triangle Institute | Research Triangle Park, North Carolina, USA | ECHO RTI Data Analysis Center Director Co-Investigator | U24OD023382 (Data Analysis Center) | christiand@rti.org |
| Priya | Duggal |  | PhD | Department of Epidemiology | Johns Hopkins University, Bloomberg School of Public Health | Baltimore, Maryland, USA | ECHO Data Analysis Center Genetics Methods Lead, Co-Investigator | U24OD023382 (Data Analysis Center) | pduggal@jhu.edu |
| Emily | Knapp |  | PhD | Department of Epidemiology | Johns Hopkins University, Bloomberg School of Public Health | Baltimore, Maryland, USA | ECHO Data Analysis Center Co-Investigator | U24OD023382 (Data Analysis Center) | eknapp2@jhu.edu |
| Amii | Kress |  | PhD | Department of Epidemiology | Johns Hopkins University, Bloomberg School of Public Health | Baltimore, Maryland, USA | ECHO Data Analysis Center General Methods Co-Investigator | U24OD023382 (Data Analysis Center) | akress1@jhu.edu |
| Courtney K. | Blackwell |  | PhD | Department of Medical Social Sciences | Feinberg School of Medicine, Northwestern University | Chicago, Illinois, USA | Measurement Core Co-Investigator | U24OD023319 with co-funding from the Office of Behavioral and Social Science Research (Measurement Core) | Ckblackwell@northwestern.edu |
| Maxwell A. | Mansolf |  | PhD | Department of Medical Social Sciences | Feinberg School of Medicine, Northwestern University | Chicago, Illinois, USA | Measurement Core Co-Investigator | U24OD023319 with co-funding from the Office of Behavioral and Social Science Research (Measurement Core) | maxwell.mansolf@northwestern.edu |
| Jin-Shei | Lai |  | PhD | Department of Medical Social Sciences | Feinberg School of Medicine, Northwestern University | Chicago, Illinois, USA | Measurement Core Co-Investigator | U24OD023319 with co-funding from the Office of Behavioral and Social Science Research (Measurement Core) | js-lai@northwestern.edu |
| Emily | Ho |  | PhD | Department of Medical Social Sciences | Feinberg School of Medicine, Northwestern University | Chicago, Illinois, USA | Measurement Core Co-Investigator | U24OD023319 with co-funding from the Office of Behavioral and Social Science Research (Measurement Core) | emily-ho@northwestern.edu |
| David | Cella |  | PhD | Department of Medical Social Sciences | Feinberg School of Medicine, Northwestern University | Chicago, Illinois, USA | Measurement Core Principal Investigator | U24OD023319 with co-funding from the Office of Behavioral and Social Science Research (Measurement Core) | d-cella@northwestern.edu |
| Richard | Gershon |  | PhD | Department of Medical Social Sciences | Feinberg School of Medicine, Northwestern University | Chicago, Illinois, USA | Measurement Core Principal Investigator | U24OD023319 with co-funding from the Office of Behavioral and Social Science Research (Measurement Core) | gershon@northwestern.edu |
| Michelle L. | Macy |  | MD | Department of Pediatrics | Feinberg School of Medicine, Northwestern University and Ann & Robert H. Lurie Children's Hospital of Chicago | Chicago, Illinois, USA | Measurement Core Co-Investigator | U24OD023319 with co-funding from the Office of Behavioral and Social Science Research (Measurement Core) | [mmacy@luriechildrens.org](mailto:mmacy@luriechildrens.org) |
| Suman R. | Das |  | PhD | Division of Infectious Diseases, Department of Medicine | Vanderbilt University Medical Center | Nashville, Tennessee, USA | ECHO Laboratory Core Principal Investigator | U24OD035523 (Lab Core) | suman.r.das@vumc.org |
| Jane E. | Freedman |  | MD | Division of Cardiovascular Medicine, Department of Medicine | Vanderbilt University Medical Center | Nashville, Tennessee, USA | ECHO Laboratory Core Principal Investigator | U24OD035523 (Lab Core) | jane.freedman@vumc.org |
| Simon A. | Mallal |  | MBBS | Division of Infectious Diseases, Department of Medicine | Vanderbilt University Medical Center | Nashville, Tennessee, USA | ECHO Laboratory Core Principal Investigator | U24OD035523 (Lab Core) | s.mallal@vumc.org |
| John A. | McLean |  | PhD | Department of Chemistry | Vanderbilt University | Nashville, Tennessee, USA | ECHO Laboratory Core Principal Investigator | U24OD035523 (Lab Core) | john.a.mclean@vanderbilt.edu |
| Ravi V. | Shah |  | MD | Division of Cardiovascular Medicine, Department of Medicine | Vanderbilt University Medical Center | Nashville, Tennessee, USA | ECHO Laboratory Core Principal Investigator | U24OD035523 (Lab Core) | ravi.shah@vumc.org |
| Meghan H. | Shilts |  | MHS | Division of Infectious Diseases, Department of Medicine | Vanderbilt University Medical Center | Nashville, Tennessee, USA | ECHO Laboratory Core Principal Investigator Admin Designee | U24OD035523 (Lab Core) | [meghan.h.shilts@vumc.org](mailto:meghan.h.shilts@vumc.org) |
| Akram N. | Alshawabkeh |  | PhD | College of Engineering | Northeastern University | Boston, Massachusetts, USA | ECHO Cohort Study Site Principal Investigator | UG3/UH3OD023251 (Akram Alshawabkeh) | [a.alshawabkeh@northeastern.edu](mailto:a.alshawabkeh@northeastern.edu) |
| Jose F. | Cordero |  | MD | College of Public Health, Department of Epidemiology & Biostatistics | University of Georgia | Athens, Georgia; USA | ECHO Cohort Study Site Co-Director | UG3/UH3OD023251 (Akram Alshawabkeh) | [jcordero@uga.edu](mailto:jcordero@uga.edu) |
| John | Meeker |  | ScD | Environmental Health Sciences, School of Public Health | University of Michigan | Ann Arbor, Michigan; USA | ECHO Cohort Study Site Co-Director | UG3/UH3OD023251 (Akram Alshawabkeh) | [meekerj@umich.edu](mailto:meekerj@umich.edu) |
| Leonardo | Trasande |  | MD, MPP | Departments of Pediatrics and Population Health | NYU Grossman School of Medicine | New York, New York, USA | ECHO Cohort Study Site Principal Investigator | UG3/UH3OD023305 (Leonardo Trasande) | [leonardo.trasande@nyulangone.org](mailto:leonardo.trasande@nyulangone.org) |
| Carlos A. | Camargo | Jr. | MD, DrPH | Department of Emergency Medicine | Massachusetts General Hospital, Harvard Medical School | Boston, Massachusetts, USA | ECHO Cohort Study Site Principal Investigator | UG3/UH3OD023253 (Carlos Camargo) | [ccamargo@mgb.org](mailto:ccamargo@mgb.org) |
| Kohei | Hasegawa |  | MD, PhD | Department of Emergency Medicine | Massachusetts General Hospital, Harvard Medical School | Boston, Massachusetts, USA | ECHO Cohort Study Site Co-Investigator | UG3/UH3OD023253 (Carlos Camargo) | [khasegawa@mgh.harvard.edu](mailto:khasegawa@mgh.harvard.edu) |
| Zhaozhong | Zhu |  | ScD | Department of Emergency Medicine | Massachusetts General Hospital, Harvard Medical School | Boston, Massachusetts, USA | ECHO Cohort Study Site Co-Investigator | UG3/UH3OD023253 (Carlos Camargo) | [zzhu5@mgh.harvard.edu](mailto:zzhu5@mgh.harvard.edu) |
| Ashley F. | Sullivan |  | MS, MPH | Department of Emergency Medicine | Massachusetts General Hospital, Harvard Medical School | Boston, Massachusetts, USA | ECHO Cohort Study Site Award Project Director | UG3/UH3OD023253 (Carlos Camargo) | [afsullivan@mgb.org](mailto:afsullivan@mgb.org) |
| Dana | Dabelea |  | MD, PhD | Lifecourse Epidemiology of Adiposity and Diabetes (LEAD) Center | University of Colorado Anschutz Medical Campus | Aurora, Colorado, USA | ECHO Cohort Study Site Principal Investigator | UG3/UH3OD023248 and UG3OD035526 (Dana Dabelea) | [dana.dabelea@cuanschutz.edu](mailto:dana.dabelea@cuanschutz.edu) |
| Wei | Perng |  | PhD, MPH | Lifecourse Epidemiology of Adiposity and Diabetes (LEAD) Center | University of Colorado Anschutz Medical Campus | Aurora, Colorado, USA | ECHO Cohort Study Site Principal Investigator | UG3/UH3OD023248 (Dana Dabelea) | [wei.perng@cuanschutz.edu](mailto:wei.perng@cuanschutz.edu) |
| Traci A. | Bekelman |  | PhD, MPH | Lifecourse Epidemiology of Adiposity and Diabetes (LEAD) Center | University of Colorado Anschutz Medical Campus | Aurora, Colorado, USA | ECHO Cohort Study Site Principal Investigator | UG3/UH3OD023248 (Dana Dabelea) | [traci.bekelman@cuanschutz.edu](mailto:traci.bekelman@cuanschutz.edu) |
| Greta | Wilkening |  | PhD, MPH | Lifecourse Epidemiology of Adiposity and Diabetes (LEAD) Center | University of Colorado Anschutz Medical Campus | Aurora, Colorado, USA | ECHO Cohort Study Site Co-Investigator | UG3/UH3OD023248 (Dana Dabelea) | [Greta.Wilkening@childrenscolorado.org](mailto:Greta.Wilkening@childrenscolorado.org) |
| Sheryl | Magzamen |  | PhD | Environmental and Radiological Health Sciences | Colorado School of Public Health, Colorado State University | Fort Collins, Colorado, USA | ECHO Cohort Study Site Co-Investigator | UG3OD035526 (Dana Dabelea) | [Sheryl.Magzamen@colostate.edu](mailto:Sheryl.Magzamen@colostate.edu) |
| Brianna F. | Moore |  | PhD, MS | Lifecourse Epidemiology of Adiposity and Diabetes (LEAD) Center | University of Colorado Anschutz Medical Campus | Aurora, Colorado, USA | ECHO Cohort Study Site Principal Investigator | UG3OD035526 (Dana Dabelea) | [brianna.f.moore@cuanschutz.edu](mailto:brianna.f.moore@cuanschutz.edu) |
| Anne P. | Starling |  | PhD | Epidemiology | University of North Carolina at Chapel Hill | Chapel Hill, North Carolina, USA | ECHO Cohort Study Site Principal Investigator | UG3OD035526 (Dana Dabelea) | [anne.starling@unc.edu](mailto:anne.starling@unc.edu) |
| Deborah J. | Rinehart |  | PhD | Center for Health Systems Research | Denver Health and Hospital Authority | Denver, Colorado, USA | ECHO Cohort Study Site Co-Investigator | UG3OD035526 (Dana Dabelea) | [deborah.rinehart@dhha.org](mailto:deborah.rinehart@dhha.org) |
| Daphne | Koinis Mitchell |  | Ph.D | Department of Pediatrics | Rhode Island Hospital, The Alpert Medical School of Brown University | Providence, Rhode Island, USA | ECHO Cohort Study Site Principal Investigator | UG3/UH3OD023313 (Daphne Koinis Mitchell) | [dkoinismitchell@lifespan.org](mailto:dkoinismitchell@lifespan.org) |
| Viren | D'Sa |  | MD | Department of Pediatrics | Rhode Island Hospital, The Alpert Medical School of Brown University | Providence, Rhode Island, USA | ECHO Cohort Study Site Principal Investigator | UG3/UH3OD023313 (Daphne Koinis Mitchell) | [viren_Dsa@brown.edu](mailto:viren_Dsa@brown.edu) |
| Sean C.L. | Deoni |  | PhD | Division of Gender Equality, Maternal, Newborn & Child Health Discovery & Tools Team | Bill & Melinda Gates Foundation | Seattle, Washington, USA | ECHO Cohort Study Site Principal Investigator | UG3/UH3OD023313 (Daphne Koinis Mitchell) | [Sean.Deoni@gatesfoundation.org](mailto:Sean.Deoni@gatesfoundation.org) |
| Hans-Georg | Mueller |  | PhD | Department of Statistics | University of California, Davis | Davis, California, USA | ECHO Cohort Study Site Co-Investigator | UG3/UH3OD023313 (Daphne Koinis Mitchell) | [hgmueller@ucdavis.edu](mailto:hgmueller@ucdavis.edu) |
| Cristiane S. | Duarte |  | PhD, MPH | Division of Child and Adolescent Psychiatry | Columbia University - NYSPI | New York, New York, USA | ECHO Cohort Study Site Principal Investigator | UH3OD023328 (Cristiane Duarte) | [Cristiane.Duarte@nyspi.columbia.edu](mailto:Cristiane.Duarte@nyspi.columbia.edu) |
| Catherine | Monk |  | PhD | Department of Obstetrics & Gynecology | Columbia University - NYSPI | New York, New York, USA | ECHO Cohort Study Site Principal Investigator | UH3OD023328 (Cristiane Duarte) | [cem31@cumc.columbia.edu](mailto:cem31@cumc.columbia.edu) |
| Glorisa | Canino |  | PhD | Behavioral Sciences Research Institute | University of Puerto Rico, School of Medicine | Rio Piedras, Puerto Rico | ECHO Cohort Study Site Principal Investigator | UH3OD023328 (Cristiane Duarte) | [glorisa.canino@upr.edu](mailto:glorisa.canino@upr.edu) |
| Jonathan | Posner |  | MD | Child & Family Mental Health & Community Psychiatry Division | Duke University School of Medicine, Duke Psychiatry & Behavioral Sciences | Durham, North Carolina, USA | ECHO Cohort Study Site Principal Investigator | UH3OD023328 (Cristiane Duarte) | [jonathan.posner@duke.edu](mailto:jonathan.posner@duke.edu) |
| Tenneill | Murray |  | MPH | Division of Child and Adolescent Psychiatry | Columbia University - NYSPI | New York, New York, USA | ECHO Cohort Study Site Co-Director | UH3OD023328 (Cristiane Duarte) | [tenneill.murray@nyspi.columbia.edu](mailto:tenneill.murray@nyspi.columbia.edu) |
| Claudia | Lugo-Candelas |  | PhD | Division of Child and Adolescent Psychiatry | Columbia University - NYSPI | New York, New York, USA | ECHO Cohort Study Site Principal Investigator | UH3OD023328 (Cristiane Duarte) | [claudia.lugo@nyspi.columbia.edu](mailto:claudia.lugo@nyspi.columbia.edu) |
| Anne L. | Dunlop |  | MD, MPH | Department of Gynecology and Obstetrics | Emory University School of Medicine | Atlanta, Georgia, USA | ECHO Cohort Study Site Principal Investigator | UH3OD023318 (Anne Dunlop) | [amlang@emory.edu](mailto:amlang@emory.edu) |
| Patricia A. | Brennan |  | PhD | Department of Psychology | Emory University | Atlanta, Georgia, USA | ECHO Cohort Study Site Principal Investigator | UH3OD023318 (Anne Dunlop) | [pbren01@emory.edu](mailto:pbren01@emory.edu) |
| Christine | Hockett |  | PhD | N/A; Department of Pediatrics | Avera Research Institute; University of South Dakota School of Medicine | Rapid City, South Dakota, USA; Sioux Falls, South Dakota, USA | ECHO Cohort Study Site Principal Investigator | UG3/UH3OD023279 (Amy Elliott) | [christine.hockett@avera.org](mailto:christine.hockett@avera.org) |
| Amy | Elliott |  | PhD | N/A; Department of Pediatrics | Avera Research Institute ; University of South Dakota School of Medicine | Sioux Falls, South Dakota, USA | ECHO Cohort Study Site Principal Investigator | UG3/UH3OD023279 (Amy Elliott) | [amy.elliott@avera.org](mailto:amy.elliott@avera.org) |
| Assiamira | Ferrara |  | MD, PhD | Division of Research | Kaiser Permanente Northern California | Oakland, California, USA | ECHO Cohort Study Site Principal Investigator | UG3/UH3OD023289 (Assiamira Ferrara) | [assiamira.ferrara@kp.org](mailto:assiamira.ferrara@kp.org) |
| Lisa A. | Croen |  | PhD | Division of Research | Kaiser Permanente Northern California | Oakland, California, USA | ECHO Cohort Study Site Principal Investigator | UG3/UH3OD023342 (Kristen Lyall), UG3/UH3OD023290 (Julie Herbstman) | [Lisa.A.Croen@kp.org](mailto:Lisa.A.Croen@kp.org) |
| Monique M. | Hedderson |  | PhD | Division of Research | Kaiser Permanente Northern California | Oakland, California, USA | ECHO Cohort Study Site Principal Investigator | UG3/UH3OD023289 (Assiamira Ferrara), UG3OD035540 (Monique Marie Hedderson) | [Monique.M.Hedderson@kp.org](mailto:Monique.M.Hedderson@kp.org) |
| John | Ainsworth |  | PhD | Centre for Health Informatics | University of Manchester | Manchester, United Kingdom | ECHO Cohort Study Site Principal Investigator | UG3/UH3OD023282 (James Gern) | [John.Ainsworth@manchester.ac.uk](mailto:John.Ainsworth@manchester.ac.uk) |
| Leonard B. | Bacharier |  | MD | Department of Pediatrics, Monroe Carell Jr Children’s Hospital at Vanderbilt | Vanderbilt University Medical Center | Nashville, Tennessee, USA | ECHO Cohort Study Site Principal Investigator | UG3/UH3OD023282 (James Gern) | leonard.bacharier@vumc.org |
| Casper G. | Bendixsen |  | PhD | National Farm Medicine Center | Marshfield Clinic Research Institute | Marshfield, Wisconsin, USA | ECHO Cohort Study Site Principal Investigator | UG3/UH3OD023282 (James Gern) | [Bendixsen.casper@marshfieldresearch.org](mailto:Bendixsen.casper@marshfieldresearch.org) |
| James E. | Gern |  | MD | Department of Pediatrics | University of Wisconsin School of Medicine and Public Health | Madison, Wisconsin, USA | ECHO Cohort Study Site Principal Investigator | UG3/UH3OD023282 (James Gern), UG3OD035509 (Anne Marie Singh) | gern@medicine.wisc.edu |
| Diane R. | Gold |  | MD | The Channing Division of Network Medicine; Department of Medicine | Brigham and Women’s Hospital; Harvard Medical School | Boston, Massachusetts, USA | ECHO Cohort Study Site Principal Investigator | UG3/UH3OD023282 (James Gern) | [redrg@channing.harvard.edu](mailto:redrg@channing.harvard.edu) |
| Tina V. | Hartert |  | MD, MPH | Division of Pediatric Allergy, Immunology, and Pulmonary Medicine, Department of Medicine, Department of Pediatrics | Vanderbilt University Medical Center | Nashville, Tennessee, USA | ECHO Cohort Study Site Principal Investigator | UG3/UH3OD023282 (James Gern), UG3OD035516 and UG3OD035517 (Tina Hartert) | [tina.hartert@vumc.org](mailto:tina.hartert@vumc.org) |
| Daniel J. | Jackson |  | MD | Department of Pediatrics | University of Wisconsin School of Medicine and Public Health | Madison, Wisconsin, USA | ECHO Cohort Study Site Principal Investigator | UG3/UH3OD023282 (James Gern) | [djj@medicine.wisc.edu](mailto:djj@medicine.wisc.edu) |
| Christine C. | Johnson |  | PhD | Department of Public Health Sciences | Henry Ford Health | Detroit, Michigan, USA | ECHO Cohort Study Site Principal Investigator | UG3/UH3OD023282 (James Gern), UG3OD035518 (Jennifer Straughen) | [CJOHNSO1@hfhs.org](mailto:CJOHNSO1@hfhs.org) |
| Christine L.M. | Joseph |  | PhD | Department of Public Health Sciences | Henry Ford Health | Detroit, Michigan, USA | ECHO Cohort Study Site Principal Investigator | UG3/UH3OD023282 (James Gern) | [cjoseph1@hfhs.org](mailto:cjoseph1@hfhs.org) |
| Meyer | Kattan |  | MD | Department of Pediatrics | Columbia University Medical Center | New York, New York, USA | ECHO Cohort Study Site Principal Investigator | UG3/UH3OD023282 (James Gern) | mk2833@cumc.columbia.edu |
| Gurjit K. | Khurana Hershey |  | MD, PhD | Division of Asthma Research | Cincinnati Children’s Hospital Medical Center | Cincinnati, Ohio, USA | ECHO Cohort Study Site Principal Investigator | UG3/UH3OD023282 (James Gern) | gurjit.hershey@cchmc.org |
| Robert F. | Lemanske, Jr. |  | MD | Department of Pediatrics | University of Wisconsin School of Medicine and Public Health | Madison, Wisconsin, USA | ECHO Cohort Study Site Principal Investigator | UG3/UH3OD023282 (James Gern) | [lemanske@wisc.edu](mailto:lemanske@wisc.edu) |
| Susan V. | Lynch |  | PhD | Department of Medicine | University of California | San Francisco, California, USA | ECHO Cohort Study Site Principal Investigator | UG3/UH3OD023282 (James Gern) | [Susan.Lynch@ucsf.edu](mailto:Susan.Lynch@ucsf.edu) |
| Rachel L. | Miller |  | MD | Department of Medicine; Division of Clinical Immunology | Icahn School of Medicine at Mount Sinai | New York, New York, USA | ECHO Cohort Study Site Principal Investigator | UG3/UH3OD023282 (James Gern), UG3/UH3OD023290 (Julie Herbstman) | [Rachel.miller2@mssm.edu](mailto:Rachel.miller2@mssm.edu) |
| George T. | O’Connor |  | MD | Department of Pediatrics | Boston University School of Medicine | Boston, Massachusetts, USA | ECHO Cohort Study Site Principal Investigator | UG3/UH3OD023282 (James Gern) | goconnor@bu.edu |
| Carole | Ober |  | PhD | Department of Human Genetics | University of Chicago | Chicago, Illinois, USA | ECHO Cohort Study Site Principal Investigator | UG3/UH3OD023282 (James Gern), UG3OD035509 (Anne Marie Singh) | [c-ober@genetics.uchicago.edu](mailto:c-ober@genetics.uchicago.edu) |
| Dennis | Ownby |  | MD | Department of Public Health Sciences | Henry Ford Health | Detroit, Michigan, USA | ECHO Cohort Study Site Principal Investigator | UG3/UH3OD023282 (James Gern) | downby@augusta.edu |
| Katherine | Rivera-Spoljaric |  | MD | Department of Pediatrics | Washington University School of Medicine | St Louis, Missouri, USA | ECHO Cohort Study Site Principal Investigator | UG3/UH3OD023282 (James Gern), UG3OD035521 (Katherine Rivera-Spoljaric) | [rivera_k@wustl.edu](mailto:rivera_k@wustl.edu) |
| Patrick H. | Ryan |  | PhD | Department of Pediatrics and College of Medicine; Division of Biostatistics and Epidemiology | University of Cincinnati | Cincinnati, Ohio, USA | ECHO Cohort Study Site Principal Investigator | UG3/UH3OD023282 (James Gern), UG3OD035509 (Anne Marie Singh) | patrick.ryan@cchmc.org |
| Christine M. | Seroogy |  | MD | Department of Pediatrics | University of Wisconsin School of Medicine and Public Health | Madison, Wisconsin, USA | ECHO Cohort Study Site Principal Investigator | UG3/UH3OD023282 (James Gern) | [cmseroogy@wisc.edu](mailto:cmseroogy@wisc.edu) |
| Anne Marie | Singh |  | MD | Department of Pediatrics | University of Wisconsin School of Medicine and Public Health | Madison, Wisconsin, USA | ECHO Cohort Study Site Principal Investigator | UG3/UH3OD023282 (James Gern), UG3OD035509 (Anne Marie Singh) | [amsingh@wisc.edu](mailto:amsingh@wisc.edu) |
| Robert A. | Wood |  | MD | Department of Pediatrics | Johns Hopkins University School of Medicine | Baltimore, Maryland, USA | ECHO Cohort Study Site Principal Investigator | UG3/UH3OD023282 (James Gern) | [rwood@jhmi.edu](mailto:rwood@jhmi.edu) |
| Edward M. | Zoratti |  | MD | Division of Allergy and Clinical Immunology | Henry Ford Health | Detroit, Michigan, USA | ECHO Cohort Study Site Principal Investigator | UG3/UH3OD023282 (James Gern), UG3OD035518 (Jennifer Straughen) | ezoratt1@hfhs.org |
| Rima | Habre |  | ScD, MSc | Department of Population and Public Health Sciences | University of Southern California | Los Angeles, California, USA | ECHO Cohort Study Site Principal Investigator | UH3OD023287 (Carrie Breton) | [habre@usc.edu](mailto:habre@usc.edu) |
| Shohreh | Farzan |  | PhD | Department of Population and Public Health Sciences | University of Southern California | Los Angeles, California, USA | ECHO Cohort Study Site Principal Investigator | UH3OD023287 (Carrie Breton) | [sffarzan@usc.edu](mailto:sffarzan@usc.edu) |
| Frank D. | Gilliland |  | MD, MPH, PhD | Department of Population and Public Health Sciences | University of Southern California | Los Angeles, California, USA | ECHO Cohort Study Site Principal Investigator | UH3OD023287 (Carrie Breton) | [gillilan@usc.edu](mailto:gillilan@usc.edu) |
| Irva | Hertz-Picciotto |  | PhD | MIND Institute and Department of Public Health Sciences | University of California, Davis | Davis, California, USA | ECHO Cohort Study Site Principal Investigator | UG3/UH3OD023365 (Irva Hertz-Picciotto), UG3OD035550 (Rebecca Schmidt) | [iher@ucdavis.edu](mailto:iher@ucdavis.edu) |
| Deborah H. | Bennett |  | Ph.D | Department of Public Health Sciences | University of California, Davis | Davis, California, USA | ECHO Cohort Study Site Principal Investigator | UG3/UH3OD023365 (Irva Hertz-Picciotto), UG3OD035550 (Rebecca Schmidt) | [dhbennett@ucdavis.edu](mailto:dhbennett@ucdavis.edu) |
| Julie B. | Schweitzer |  | Ph.D | Department of Psychiatry and Behavioral Science and the MIND Institute | University of California, Davis | Davis, California, USA | ECHO Cohort Study Site Principal Investigator | UG3/UH3OD023365 (Irva Hertz-Picciotto) | [jschweitzer@ucdavis.edu](mailto:jschweitzer@ucdavis.edu) |
| Rebecca J. | Schmidt |  | Ph.D | MIND Institute and Department of Public Health Sciences | University of California, Davis | Davis, California, USA | ECHO Cohort Study Site Principal Investigator | UG3/UH3OD023365 (Irva Hertz-Picciotto), UG3/UH3OD023342 (Kristen Lyall), UG3OD035550 (Rebecca Schmidt) | [rjschmidt@ucdavis.edu](mailto:rjschmidt@ucdavis.edu) |
| Janine M. | LaSalle |  | PhD | Medical Microbiology and Immunology; MIND Institute | University of California, Davis | Davis, California, USA | ECHO Cohort Study Site Co-Investigator | UG3/UH3OD023365 (Irva Hertz-Picciotto), UG3OD035550 (Rebecca Schmidt) | [jmlasalle@ucdavis.edu](mailto:jmlasalle@ucdavis.edu) |
| Alison E. | Hipwell |  | PhD, ClinPsyD | Psychiatry and Psychology | University of Pittsburgh | Pittsburgh, Pennsylvania, USA | ECHO Cohort Study Site Principal Investigator | UG3/UH3OD023244 (Alison Hipwell) | [hipwae@upmc.edu](mailto:hipwae@upmc.edu) |
| Kate E. | Keenan |  | PhD | Psychiatry and Behavioral Neuroscience | University of Chicago | Chicago, Illinois, USA | ECHO Cohort Study Site Principal Investigator | UG3/UH3OD023244 (Alison Hipwell) | [kekeenan@uchicago.edu](mailto:kekeenan@uchicago.edu) |
| Catherine J. | Karr |  | MD, MS, PhD | Department of Pediatrics, School of Medicine; Department of Environmental and Occupational Health Sciences; School of Public Health | University of Washington | Seattle, Washington, USA | ECHO Cohort Study Site Principal Investigator | UH3OD023271 and UG3OD035528 (Catherine Karr) | [ckarr@uw.edu](mailto:ckarr@uw.edu) |
| Nicole R. | Bush |  | PhD | Department of Psychiatry and Behavioral Sciences and Department of Pediatrics, School of Medicine | University of California, San Francisco | San Francisco, California, USA | ECHO Cohort Study Site Principal Investigator | UH3OD023271 (Catherine Karr), UG3OD035519 (Qi Zhao) | nicole.bush@ucsf.edu |
| Kaja Z. | LeWinn |  | ScD | Department of Psychiatry and Behavioral Sciences, School of Medicine | University of California, San Francisco | San Francisco, California, USA | ECHO Cohort Study Site Principal Investigator | UH3OD023271 (Catherine Karr), UG3OD035519 (Qi Zhao) | kaja.lewinn@ucsf.edu |
| Sheela | Sathyanarayana |  | MD, MPH | Department of Pediatrics, School of Medicine; Department of Environmental and Occupational Health Sciences, School of Public Health | University of Washington and Seattle Children's Research Institute | Seattle, Washington, USA | ECHO Cohort Study Site Principal Investigator | UH3OD023271 (Catherine Karr), UG3OD035508 (Sheela Sathyanarayana) | sheela.sathyanarayana@seattlechildrens.org |
| Qi | Zhao |  | MD, PhD | Department of Preventive Medicine | University of Tennessee Health Science Center | Memphis, Tennessee, USA | ECHO Cohort Study Site Principal Investigator | UH3OD023271 (Catherine Karr), UG3OD035519 (Qi Zhao) | qzhao11@uthsc.edu |
| Frances | Tylavsky |  | DrPH, MS | Department of Preventive Medicine | University of Tennessee Health Science Center | Memphis, Tennessee, USA | ECHO Cohort Study Site Principal Investigator | UH3OD023271 (Catherine Karr) | ftylavsk@uthsc.edu |
| Kecia N. | Carroll |  | MD, MPH | Department of Pediatrics, Department of Environmental Medicine & Public Health | Icahn School of Medicine at Mount Sinai | New York, New York, USA | ECHO Cohort Study Site Principal Investigator | UH3OD023271 (Catherine Karr), UG3/UH3OD023337 (Rosalind Wright) | kecia.carroll@mssm.edu |
| Christine T. | Loftus |  | MS MPH PhD | Department of Environmental and Occupational Health Sciences; School of Public Health | University of Washington | Seattle, Washington, USA | ECHO Cohort Study Site Principal Investigator | UH3OD023271 (Catherine Karr) | [cloftus@uw.edu](mailto:cloftus@uw.edu) |
| Leslie D. | Leve |  | PhD | Department of Counseling Psychology and Human Services & Prevention Science Institute | University of Oregon | Eugene, Oregon, USA | ECHO Cohort Study Site Principal Investigator | UG3/UH3OD023389 (Leslie Leve) | [leve@uoregon.edu](mailto:leve@uoregon.edu) |
| Jody M. | Ganiban |  | PhD | Department of Psychological and Behavioral Sciences | George Washington University | Washington, DC, USA | ECHO Cohort Study Site Principal Investigator | UG3/UH3OD023389 (Leslie Leve) | [ganiban@gwu.edu](mailto:ganiban@gwu.edu) |
| Jenae M. | Neiderhiser |  | PhD | Department of Psychology | Penn State University | University Park, Pennsylvania, USA | ECHO Cohort Study Site Principal Investigator | UG3/UH3OD023389 (Leslie Leve) | [jenaemn@psu.edu](mailto:jenaemn@psu.edu) |
| Scott T. | Weiss |  | MD | Channing Division of Network Medicine, Department of Medicine | Brigham and Women's Hospital and Harvard Medical School | Boston, Massachusetts, USA | ECHO Cohort Study Site Principal Investigator | UH3OD023268 (Scott Weiss) | [scott.weiss@channing.harvard.edu](mailto:scott.weiss@channing.harvard.edu) |
| Augusto A. | Litonjua |  | MD | Pediatric Pulmonary Division, Department of Pediatrics | Golisano Children's Hospital, University of Rochester | Rochester, New York, USA | ECHO Cohort Study Site Principal Investigator | UH3OD023268 (Scott Weiss) | augusto_litonjua@urmc.rochester.edu |
| Cindy T. | McEvoy |  | MD, MCR | Division of Neonatology, Department of Pediatrics | Oregon Health & Science University | Portland, Oregon, USA | ECHO Cohort Study Site Principal Investigator | UG3/UH3OD023288 (Cynthia McEvoy) | [mcevoyc@ohsu.edu](mailto:mcevoyc@ohsu.edu) |
| Eliot R. | Spindel |  | MD, PhD | Division of Neuroscience | Oregon National Primate Research Center | Beaverton, Oregon, USA | ECHO Cohort Study Site Principal Investigator | UG3/UH3OD023288 (Cynthia McEvoy) | [spindele@ohsu.edu](mailto:spindele@ohsu.edu) |
| Robert S. | Tepper |  | MD, PhD | Division of Pediatric Pulmonology, Department of Pediatrics | Indiana School of Medicine | Indianapolis, Indiana, USA | ECHO Cohort Study Site Co-Investigator | UG3/UH3OD023288 (Cynthia McEvoy) | [rtepper@iu.edu](mailto:rtepper@iu.edu) |
| Craig J. | Newschaffer |  | PhD | College of Health and Human Development | Penn State | State College, Pennsylvania, USA | ECHO Cohort Study Site Principal Investigator | UG3/UH3OD023342 (Kristen Lyall) | [newschaffer@psu.edu](mailto:newschaffer@psu.edu) |
| Kristen | Lyall |  | ScD | AJ Drexel Autism Institute | Drexel University | Philadelphia, Pennsylvania, USA | ECHO Cohort Study Site Principal Investigator | UG3/UH3OD023342 (Kristen Lyall) | [kld98@drexel.edu](mailto:kld98@drexel.edu) |
| Heather E. | Volk |  | PhD | Mental Health | Johns Hopkins University | Baltimore, Maryland, USA | ECHO Cohort Study Site Principal Investigator | UG3/UH3OD023342 (Kristen Lyall) | [hvolk1@jhu.edu](mailto:hvolk1@jhu.edu) |
| Rebecca | Landa |  | PhD | Department of Psychiatry and Behavioral Sciences | Center for Autism and Related Disorders, Kennedy Krieger Institute, Johns Hopkins University | Baltimore, Maryland, USA | ECHO Cohort Study Site Co-Investigator | UG3/UH3OD023342 (Kristen Lyall) | [landa@kennedykrieger.org](mailto:landa@kennedykrieger.org) |
| Sally | Ozonoff |  | PhD | MIND Institute, Department of Psychiatry | University of California Davis | Sacramento, California, USA | ECHO Cohort Study Site Co-Investigator | UG3/UH3OD023342 (Kristen Lyall) | [sozonoff@ucdavis.edu](mailto:sozonoff@ucdavis.edu) |
| Joseph | Piven |  | MD | Department of Psychiatry | University of North Carolina | Chapel Hill, North Carolina, USA | ECHO Cohort Study Site Co-Investigator | UG3/UH3OD023342 (Kristen Lyall) | [jpiven@med.unc.edu](mailto:jpiven@med.unc.edu) |
| Heather | Hazlett |  | PhD | Department of Psychiatry | University of North Carolina | Chapel Hill, North Carolina, USA | ECHO Cohort Study Site Co-Investigator | UG3/UH3OD023342 (Kristen Lyall) | [heather_cody@med.unc.edu](mailto:heather_cody@med.unc.edu) |
| Juhi | Pandey |  | PhD | Center for Autism Research | Children's Hospital of Philadelphia | Philadelphia, Pennsylvania, USA | ECHO Cohort Study Site Co-Investigator | UG3/UH3OD023342 (Kristen Lyall) | [pandeyj@chop.edu](mailto:pandeyj@chop.edu) |
| Robert | Schultz |  | PhD | Center for Autism Research | Children's Hospital of Philadelphia | Philadelphia, Pennsylvania, USA | ECHO Cohort Study Site Co-Investigator | UG3/UH3OD023342 (Kristen Lyall) | [schultzrt@chop.edu](mailto:schultzrt@chop.edu) |
| Steven | Dager |  | PhD | Department of Radiology | University of Washington | Seattle, Washington, USA | ECHO Cohort Study Site Co-Investigator | UG3/UH3OD023342 (Kristen Lyall) | [srd@uw.edu](mailto:srd@uw.edu) |
| Kelly | Botteron |  | PhD | Department of Psychiatry | Washington University | St Louis, Missouri, USA | ECHO Cohort Study Site Co-Investigator | UG3/UH3OD023342 (Kristen Lyall) | [botteronk@wustl.edu](mailto:botteronk@wustl.edu) |
| Daniel | Messinger |  | PhD | Department of Psychology | University of Miami | Miami, Florida, USA | ECHO Cohort Study Site Co-Investigator | UG3/UH3OD023342 (Kristen Lyall) | [dmessinger@miami.edu](mailto:dmessinger@miami.edu) |
| Wendy | Stone |  | PhD | Department of Psychology | University of Washington | Seattle, Washington, USA | ECHO Cohort Study Site Co-Investigator | UG3/UH3OD023342 (Kristen Lyall) | [stonew@uw.edu](mailto:stonew@uw.edu) |
| Jennifer | Ames |  | PhD | Kaiser Permanente Division of Research | Kaiser Permanente | Oakland, California, USA | ECHO Cohort Study Site Co-Investigator | UG3/UH3OD023342 (Kristen Lyall) | [Jennifer.L.Ames@kp.org](mailto:Jennifer.L.Ames@kp.org) |
| Thomas G. | O'Connor |  | PhD | Departments of Psychiatry, Neuroscience, Obstetrics and Gynecology | University of Rochester | Rochester, New York, USA | ECHO Cohort Study Site Principal Investigator | UG3/UH3OD023349 (Thomas O’Connor) | [tom_oconnor@urmc.Rochester.edu](mailto:tom_oconnor@urmc.Rochester.edu) |
| Richard K. | Miller |  | PhD | Departments of Obstetrics and Gynecology | University of Rochester | Rochester, New York, USA | ECHO Cohort Study Site Principal Investigator | UG3/UH3OD023349 (Thomas O’Connor) | richardk_miller@urmc.rochester.edu |
| Emily | Oken |  | MD, MPH | Division of Chronic Disease Research Across the Lifecourse, Department of Population Medicine | Harvard Pilgrim Health Care Institute and Harvard Medical School | Boston, Massachusetts, USA | ECHO Cohort Study Site Principal Investigator | UH3OD023286 and UG3OD035533 (Emily Oken) | [emily_oken@hms.harvard.edu](mailto:emily_oken@hms.harvard.edu) |
| Michele R. | Hacker |  | ScD | Department of Obstetrics and Gynecology | Beth Israel Deaconess Medical Center | Boston, Massachusetts, USA | ECHO Cohort Study Site Principal Investigator | UG3OD035533 (Emily Oken) | [mhacker@bidmc.harvard.edu](mailto:mhacker@bidmc.harvard.edu) |
| Tamarra | James-Todd |  | PhD | Department of Environmental Health | Harvard Chan School of Public Health | Boston, Massachusetts, USA | ECHO Cohort Study Site Principal Investigator | UG3OD035533 (Emily Oken) | [tjtodd@hsph.harvard.edu](mailto:tjtodd@hsph.harvard.edu) |
| T. Michael | O'Shea | Jr | MD, MPH | Division of Neonatology, Department of Pediatrics | University of North Carolina School of Medicine | Chapel Hill, North Carolina, USA | ECHO Cohort Study Site Principal Investigator | UG3/UH3OD023348 (Mike O’Shea), UH3OD023347 (Barry Lester) | [moshea52@email.unc.edu](mailto:moshea52@email.unc.edu) |
| Rebecca C. | Fry |  | PhD | Department of Environmental Sciences and Engineering | University of North Carolina Gillings School of Global Public Health | Chapel Hill, North Carolina, USA | ECHO Cohort Study Site Principal Investigator | UG3/UH3OD023348 (Mike O’Shea) | [rfry@unc.edu](mailto:rfry@unc.edu) |
| Jean A. | Frazier |  | MD | EK Shriver Center and Psychiatry | UMASS Chan Medical School | Worcster, Massachusetts, USA | ECHO Cohort Study Site Co-Investigator | UG3/UH3OD023348 (Mike O’Shea) | [jean.frazier@umassmed.edu](mailto:jean.frazier@umassmed.edu) |
| Rachana | Singh |  | MD, MS | Department of Pediatrics | Tufts University School of Medicine | Boston, Massachusetts, USA | ECHO Cohort Study Site Co-Investigator | UG3/UH3OD023348 (Mike O’Shea) | [jean.frazier@umassmed.edu](mailto:jean.frazier@umassmed.edu) |
| Caitlin | Rollins |  | MD, SM | Department of Neurology | Harvard Medical School | Boston, Massachusetts, USA | ECHO Cohort Study Site Co-Investigator | UG3/UH3OD023348 (Mike O’Shea) | [Rachana.Singh1@tuftsmedicine.org](mailto:Rachana.Singh1@tuftsmedicine.org) |
| Angela | Montgomery |  | MD | Division of Neonatology, Department of Pediatrics | Yale School of Medicine | New Haven, Connecticut, USA | ECHO Cohort Study Site Co-Investigator | UG3/UH3OD023348 (Mike O’Shea) | [angela.montgomery@yale.edu](mailto:angela.montgomery@yale.edu) |
| Ruben | Vaidya |  | MD | Department of Pediatrics | University of Massachusetts Chan Medical School-Baystate | Springfield, Massachusetts, USA | ECHO Cohort Study Site Co-Investigator | UG3/UH3OD023348 (Mike O’Shea) | [Ruben.VaidyaMD@baystatehealth.org](mailto:Ruben.VaidyaMD@baystatehealth.org) |
| Robert M. | Joseph |  | PhD | Department of Anatomy & Neurobiology | Boston University Chobanian & Avedisian School of Medicine | Boston, Massachusetts, USA | ECHO Cohort Study Site Co-Investigator | UG3/UH3OD023348 (Mike O’Shea) | [rmjoseph@bu.edu](mailto:rmjoseph@bu.edu) |
| Lisa K. | Washburn |  | MD | Pediatrics | Wake Forest School of Medicine | Winston-Salem, North Carolina, USA | ECHO Cohort Study Site Co-Investigator | UG3/UH3OD023348 (Mike O’Shea) | [lwcadmus@gmail.com](mailto:lwcadmus@gmail.com) |
| Semsa | Gogcu |  | MD, MPH | Section of Neonatology, Department of Pediatrics; Department of Pediatrics | Wake Forest School of Medicine; Wake Forest University School of Medicine/Atrium Health Wake Forest | Winston-Salem, North Carolina, USA | ECHO Cohort Study Site Co-Investigator | UG3/UH3OD023348 (Mike O’Shea), UG3OD035513 (Annemarie Stroustrup), UH3OD023320 (Judy Aschner) | [sgogcu@wakehealth.edu](mailto:sgogcu@wakehealth.edu) |
| Kelly | Bear |  | DO | Section of Neonatology, Department of Pediatrics | ECU Health | Greenville, North Carolina, USA | ECHO Cohort Study Site Co-Investigator | UG3/UH3OD023348 (Mike O’Shea) | [BEARK17@ECU.EDU](mailto:BEARK17@ECU.EDU) |
| Julie V. | Rollins |  | MA | Division of Neonatology, Department of Pediatrics | University of North Carolina School of Medicine | Chapel Hill, North Carolina, USA | ECHO Cohort Study Site Award Project Director | UG3/UH3OD023348 (Mike O’Shea) | [julie.rollins@unc.edu](mailto:julie.rollins@unc.edu) |
| Stephen R. | Hooper |  | PhD | Department of Health Sciences | School of Medicine, University of North Carolina at Chapel Hill | Chapel Hill, North Carolina, USA | ECHO Cohort Study Site Co-Investigator | UG3/UH3OD023348 (Mike O’Shea) | [stephen_hooper@med.unc.edu](mailto:stephen_hooper@med.unc.edu) |
| Genevieve | Taylor |  | MD | Pediatrics | School of Medicine, University of North Carolina at Chapel Hill | Chapel Hill, North Carolina, USA | ECHO Cohort Study Site Co-Investigator | UG3/UH3OD023348 (Mike O’Shea) | [gtaylor@med.unc.edu](mailto:gtaylor@med.unc.edu) |
| Wesley | Jackson |  | MD, MPH | Division of Neonatology, Department of Pediatrics | University of North Carolina School of Medicine | Chapel Hill, North Carolina, USA | ECHO Cohort Study Site Co-Investigator | UG3/UH3OD023348 (Mike O’Shea) | [wesley.jackson@unc.edu](mailto:wesley.jackson@unc.edu) |
| Amanda | Thompson |  | PhD | Department of Anthropology, Department of Nutrition | University of North Carolina at Chapel Hill; Gillings School of Global Public Health, University of North Carolina at Chapel Hill | Chapel Hill, North Carolina, USA | ECHO Cohort Study Site Co-Investigator | UG3/UH3OD023348 (Mike O’Shea) | [althomps@email.unc.edu](mailto:althomps@email.unc.edu) |
| Julie | Daniels |  | PhD | Epidemiology and Maternal and Child Health | University of North Carolina at Chapel Hill; Gillings School of Global Public Health, University of North Carolina at Chapel Hill | Chapel Hill, North Carolina, USA | ECHO Cohort Study Site Co-Investigator | UG3/UH3OD023348 (Mike O’Shea) | [julie_daniels@unc.edu](mailto:julie_daniels@unc.edu) |
| Michelle | Hernandez |  | MD | Pediatrics | School of Medicine, University of North Carolina at Chapel Hill | Chapel Hill, North Carolina, USA | ECHO Cohort Study Site Co-Investigator | UG3/UH3OD023348 (Mike O’Shea) | [michelle_hernandez@med.unc.edu](mailto:michelle_hernandez@med.unc.edu) |
| Kun | Lu |  | PhD | Environmental Sciences and Engineering | Gillings School of Global Public Health, University of North Carolina at Chapel Hill | Chapel Hill, North Carolina, USA | ECHO Cohort Study Site Co-Investigator | UG3/UH3OD023348 (Mike O’Shea) | kunlu@unc.edu |
| Michael | Msall |  | MD | Kennedy Research Center on Intellectual and Neurodevelopmental Disabilities | University of Chicago Medicine: Comer Children's Hospital | Chicago Illinois, USA | ECHO Cohort Study Site Co-Investigator | UG3/UH3OD023348 (Mike O’Shea) | [mmsall@peds.bsd.uchicago.edu](mailto:mmsall@peds.bsd.uchicago.edu) |
| Madeleine | Lenski |  | MSPH | Department of Epidemiology and Biostatistics | Michigan State University | East Lansing, Michigan, USA | ECHO Cohort Study Site Co-Investigator | UG3/UH3OD023348 (Mike O’Shea) | [lenskim@msu.edu](mailto:lenskim@msu.edu) |
| Rawad | Obeid |  | MD | Pediatrics | Beaumont Hospital | Royal Oak, Michigan, USA | ECHO Cohort Study Site Co-Investigator | UG3/UH3OD023348 (Mike O’Shea) | [Rawad.Obeid@beaumont.org](mailto:Rawad.Obeid@beaumont.org) |
| Steven L. | Pastyrnak |  | PhD | Pediatrics | Corewell Health, Helen DeVos Children's Hospital | Grand Rapids, Michigan, USA | ECHO Cohort Study Site Co-Investigator | UG3/UH3OD023348 (Mike O’Shea), UH3OD023347 (Barry Lester) | [Steve.Pastyrnak@helendevoschildrens.org](mailto:Steve.Pastyrnak@helendevoschildrens.org) |
| Elizabeth | Jensen |  | PhD | Epidemiology and Prevention | Wake Forest University School of Medicine | Winston-Salem, North Carolina, USA | ECHO Cohort Study Site Co-Investigator | UG3/UH3OD023348 (Mike O’Shea) | [ejensen@wakehealth.edu](mailto:ejensen@wakehealth.edu) |
| Christina | Sakai |  | MD | Pediatrics | Mass General Hospital for Children | Boston, Massachusetts, USA | ECHO Cohort Study Site Co-Investigator | UG3/UH3OD023348 (Mike O’Shea) | [Christina.sakai@gmail.com](mailto:Christina.sakai@gmail.com) |
| Hudson | Santos |  | RN, PhD | Dean's Office Graduate School, School of Nursing and Health Studies | University of Miami | Coral Gables, Florida, USA | ECHO Cohort Study Site Principal Investigator | UG3/UH3OD023348 (Mike O’Shea), UG3OD035542 (Hudson Santos) | [hsantos@miami.edu](mailto:hsantos@miami.edu) |
| Jean M. | Kerver |  | PhD, MSc, RD | Departments of Epidemiology & Biostatistics, and Pediatrics & Human Development | Michigan State University, College of Human Medicine | East Lansing, Michigan, USA | ECHO Cohort Study Site Principal Investigator | UG3/UH3OD023285 (Jean Kerver) | [kerverje@msu.edu](mailto:kerverje@msu.edu) |
| Nigel | Paneth |  | MD, MPH | Departments of Epidemiology & Biostatistics, and Pediatrics & Human Development | Michigan State University, College of Human Medicine | East Lansing, Michigan, USA | ECHO Cohort Study Site Principal Investigator | UG3/UH3OD023285 (Jean Kerver) | [paneth@msu.edu](mailto:paneth@msu.edu) |
| Charles J. | Barone | II | MD, FAAP | Department of Pediatrics | Henry Ford Health | Detroit, Michigan, USA | ECHO Cohort Study Site Principal Investigator | UG3/UH3OD023285 (Jean Kerver), UG3/UH3OD023282 (James Gern) | [cbarone1@hfhs.org](mailto:cbarone1@hfhs.org) |
| Michael R. | Elliott |  | PhD | Department of Biostatistics | University of Michigan | Ann Arbor, Michigan, USA | ECHO Cohort Study Site Principal Investigator | UG3/UH3OD023285 (Jean Kerver) | [mrelliot@umich.edu](mailto:mrelliot@umich.edu) |
| Douglas M. | Ruden |  | PhD | Department of Obstetrics and Gynecology, Institute of Environmental Health Sciences (IEHS), C.S. Mott Center for Human Health and Development | Wayne State University | Detroit, Michigan, USA | ECHO Cohort Study Site Principal Investigator | UG3/UH3OD023285 (Jean Kerver) | [douglasr@wayne.edu](mailto:douglasr@wayne.edu) |
| Chris | Fussman |  | MS | Lifecourse Epidemiology and Genomics Division | Michigan Department of Health and Human Services (MDHHS) | Lansing, Michigan, USA | ECHO Cohort Study Site Principal Investigator | UG3/UH3OD023285 (Jean Kerver) | [fussmanc@michigan.gov](mailto:fussmanc@michigan.gov) |
| Julie B. | Herbstman |  | PhD | Department of Environmental Health Sciences | Columbia University Mailman School of Public Health | New York, New York, USA | ECHO Cohort Study Site Principal Investigator | UG3/UH3OD023290 (Julie Herbstman) | [jh2678@cumc.columbia.edu](mailto:jh2678@cumc.columbia.edu) |
| Amy | Margolis |  | PhD | Department of Psychiatry | Columbia University Irving Medical Center | New York, New York, USA | ECHO Cohort Study Site Principal Investigator | UG3/UH3OD023290 (Julie Herbstman) | amy.margolis@nyspi.columbia.edu |
| Susan L. | Schantz |  | PhD | Beckman Institute for Advanced Science and Technology; Department of Comparative Biosciences | University of Illinois Urbana-Champaign | Urbana, Illinois, USA | ECHO Cohort Study Site Principal Investigator | UG3/UH3OD023272 (Susan Schantz) | [schantz@illinois.edu](mailto:schantz@illinois.edu) |
| Sarah Dee | Geiger |  | PhD | Beckman Institute for Advanced Science and Technology; Department of Kinesiology and Community Health | University of Illinois Urbana-Champaign | Urbana, Illinois, USA | ECHO Cohort Study Site Co-Investigator | UG3/UH3OD023272 (Susan Schantz) | [smurphy7@illinois.edu](mailto:smurphy7@illinois.edu) |
| Andrea | Aguiar |  | PhD | Beckman Institute for Advanced Science and Technology; Department of Comparative Biosciences | University of Illinois Urbana-Champaign | Urbana, Illinois, USA | ECHO Cohort Study Site Co-Investigator | UG3/UH3OD023272 (Susan Schantz) | [aaguiar@illinois.edu](mailto:aaguiar@illinois.edu) |
| Karen | Tabb |  | PhD, MSW | Beckman Institute for Advanced Science and Technology; Department of Social Work | University of Illinois Urbana-Champaign | Urbana, Illinois, USA | ECHO Cohort Study Site Co-Investigator | UG3/UH3OD023272 (Susan Schantz) | [ktabb@illinois.edu](mailto:ktabb@illinois.edu) |
| Rita | Strakovsky |  | PhD | Department of Food Science and Human Nutrition | Michigan State University | East Lansing, Michigan, USA | ECHO Cohort Study Site Co-Investigator | UG3/UH3OD023272 (Susan Schantz) | [strakovs@msu.edu](mailto:strakovs@msu.edu) |
| Tracey | Woodruff |  | PhD, MPH | Program on Reproductive Health and the Environment | University of California, San Francisco | San Francisco, California, USA | ECHO Cohort Study Site Principal Investigator | UG3/UH3OD023272 (Susan Schantz) | [tracey.woodruff@ucsf.edu](mailto:tracey.woodruff@ucsf.edu) |
| Rachel | Morello-Frosch |  | PhD, MPH | Department of Environmental Science, Policy and Management and School of Public Health | University of California, Berkeley | Berkeley, California, USA | ECHO Cohort Study Site Principal Investigator | UG3/UH3OD023272 (Susan Schantz) | [rmf@berkeley.edu](mailto:rmf@berkeley.edu) |
| Amy | Padula |  | PhD | Program on Reproductive Health and the Environment | University of California, San Francisco | San Francisco, California, USA | ECHO Cohort Study Site Co-Investigator | UG3/UH3OD023272 (Susan Schantz) | [amy.padula@ucsf.edu](mailto:amy.padula@ucsf.edu) |
| Joseph B. | Stanford |  | MD, MSPH | Department of Family and Preventive Medicine | Spencer Fox Eccles School of Medicine, University of Utah | Salt Lake City, Utah, USA | ECHO Cohort Study Site Principal Investigator | UG3/UH3OD023249 (Joseph Stanford) | [joseph.stanford@utah.edu](mailto:joseph.stanford@utah.edu) |
| Christina A. | Porucznik |  | PhD, MSPH | Department of Family and Preventive Medicine | Spencer Fox Eccles School of Medicine, University of Utah | Salt Lake City, Utah, USA | ECHO Cohort Study Site Principal Investigator | UG3/UH3OD023249 (Joseph Stanford) | [christy.porucznik@utah.edu](mailto:christy.porucznik@utah.edu) |
| Angelo P. | Giardino |  | MD, PhD | Department of Pediatrics | Spencer Fox Eccles School of Medicine, University of Utah | Salt Lake City, Utah, USA | ECHO Cohort Study Site Principal Investigator | UG3/UH3OD023249 (Joseph Stanford) | [giardino@hsc.utah.edu](mailto:giardino@hsc.utah.edu) |
| Rosalind J. | Wright |  | MD, MPH | Department of Environmental Medicine & Public Health | Icahn School of Medicine at Mount Sinai | New York, New York, USA | ECHO Cohort Study Site Principal Investigator | UG3/UH3OD023337 (Rosalind Wright) | [rosalind.wright@mssm.edu](mailto:rosalind.wright@mssm.edu) |
| Robert O. | Wright |  | MD, MPH | Department of Environmental Medicine & Public Health | Icahn School of Medicine at Mount Sinai | New York, New York, USA | ECHO Cohort Study Site Principal Investigator | UG3/UH3OD023337 (Rosalind Wright) | [robert.wright@mssm.edu](mailto:robert.wright@mssm.edu) |
| Brent | Collett |  | PhD | Department of Psychiatry and Behavioral Medicine | University of Washington, Seattle Children's Research Institute | Seattle, Washington, USA | ECHO Cohort Study Site Principal Investigator | UG3OD035508 (Sheela Sathyanarayana) | [brent.collett@seattlechildrens.org](mailto:brent.collett@seattlechildrens.org) |
| Nicole | Baumann-Blackmore |  | MD | Department of Pediatrics | University of Wisconsin School of Medicine and Public Health | Madison, Wisconsin, USA | ECHO Cohort Study Site Co-Investigator | UG3OD035509 (Anne Marie Singh) | nlbaumann@wisc.edu |
| Ronald | Gangnon |  | PhD | Department of Population Health Sciences | University of Wisconsin | Madison, Wisconsin, USA | ECHO Cohort Study Site Co-Investigator | UG3OD035509 (Anne Marie Singh) | [ronald@biostat.wisc.edu](mailto:ronald@biostat.wisc.edu) |
| Daniel J. | Jackson |  | MD | Department of Pediatrics | University of Wisconsin School of Medicine and Public Health | Madison, Wisconsin, USA | ECHO Cohort Study Site Co-Investigator | UG3OD035509 (Anne Marie Singh) | [djj@medicine.wisc.edu](mailto:djj@medicine.wisc.edu) |
| Chris G. | McKennan |  | PhD | Department of Statistics | University of Pittsburgh | Pittsburgh, Pennsylvania, USA | ECHO Cohort Study Site Co-Investigator | UG3OD035509 (Anne Marie Singh) | [CHM195@pitt.edu](mailto:CHM195@pitt.edu) |
| Jo | Wilson |  | MD | Department of Pediatrics | University of Wisconsin School of Medicine and Public Health | Madison, Wisconsin, USA | ECHO Cohort Study Site Co-Investigator | UG3OD035509 (Anne Marie Singh) | wilson54@wisc.edu |
| Matt | Altman |  | MD | Department of Medicine | University of Washington | Seattle, Washington, USA | ECHO Cohort Study Site Co-Investigator | UG3OD035509 (Anne Marie Singh) | maltman@benaroyaresearch.org |
| Judy L. | Aschner |  | MD | Department of Pediatrics | Albert Einstein College of Medicine; Hackensack Meridian School of Medicine; Center for Discovery and Innovation | Bronx, New York, USA; Nutley, New Jersey, USA | ECHO Cohort Study Site Principal Investigator | UH3OD023320 and UG3OD035546 (Judy Aschner), UG3OD035513 (Annemarie Stroustrup) | [judy.aschner@einsteinmed.edu; judy.aschner@hmhn.org](mailto:judy.aschner@einsteinmed.edu) |
| Annemarie | Stroustrup |  | MD, MPH | Department of Pediatrics | Northwell Health, Cohen Children's Medical Center, and the Zucker School of Medicine at Hofstra / Northwell | New Hyde Park, New York, USA | ECHO Cohort Study Site Principal Investigator | UH3OD023320 (Judy Aschner), UG3OD035513 (Annemarie Stroustrup) | [astroustrup@northwell.edu](mailto:astroustrup@northwell.edu) |
| Stephanie L. | Merhar |  | MD, MS | Department of Pediatrics | Cincinnati Children's | Cincinnati, Ohio, USA | ECHO Cohort Study Site Co-Investigator | UH3OD023320 (Judy Aschner), UG3OD035513 (Annemarie Stroustrup) | [stephanie.merhar@cchmc.org](mailto:stephanie.merhar@cchmc.org) |
| Paul E. | Moore |  | MD | Department of Pediatrics | Vanderbilt University Medical Center | Nashville, Tennessee, USA | ECHO Cohort Study Site Co-Investigator | UH3OD023320 (Judy Aschner), UG3OD035513 (Annemarie Stroustrup) | [paul.moore@vumc.org](mailto:paul.moore@vumc.org) |
| Gloria S. | Pryhuber |  | MD | Department of Pediatrics | University of Rochester Medical Center | Rochester, New York, USA | ECHO Cohort Study Site Co-Investigator | UH3OD023320 (Judy Aschner) | [gloria_pryhuber@urmc.rochester.edu](mailto:gloria_pryhuber@urmc.rochester.edu) |
| Mark | Hudak |  | MD | Department of Pediatrics | University of Florida College of Medicine | Jacksonville, Florida, USA | ECHO Cohort Study Site Co-Investigator | UH3OD023320 (Judy Aschner) | [mark.hudak@jax.ufl.edu](mailto:mark.hudak@jax.ufl.edu) |
| Ann Marie | Reynolds Lyndaker |  | MD, MPH | Department of Pediatrics | University of Buffalo Jacobs School of Medicine and Biomedical Sciences | Buffalo, New York, USA | ECHO Cohort Study Site Co-Investigator | UH3OD023320 (Judy Aschner) | [amr1@buffalo.edu](mailto:amr1@buffalo.edu) |
| Andrea L. | Lampland |  | MD | Department of Pediatrics | Children's Minnesota | Minneapolis, Minnesota, USA | ECHO Cohort Study Site Co-Investigator | UH3OD023320 (Judy Aschner) | [andrea.lampland@childrensms.org](mailto:andrea.lampland@childrensms.org) |
| Burton | Rochelson |  | MD | Department of Obstetrics and Gynecology | Northwell Health and the Zucker School of Medicine at Hofstra / Northwell | New Hyde Park, New York, USA | ECHO Cohort Study Site Principal Investigator | UG3OD035532 (Annemarie Stroustrup) | [brochels@northwell.edu](mailto:brochels@northwell.edu) |
| Sophia | Jan |  | MD, MSHP | Department of Pediatrics | Northwell Health, Cohen Children's Medical Center, and the Zucker School of Medicine at Hofstra / Northwell | New Hyde Park, New York, USA | ECHO Cohort Study Site Co-Investigator | UG3OD035532 (Annemarie Stroustrup) | [sjan1@northwell.edu](mailto:sjan1@northwell.edu) |
| Matthew J. | Blitz |  | MD, MBA | Department of Obstetrics and Gynecology | Northwell Health and the Zucker School of Medicine at Hofstra / Northwell | New Hyde Park, New York, USA | ECHO Cohort Study Site Co-Investigator | UG3OD035532 (Annemarie Stroustrup) | [mblitz@northwell.edu](mailto:mblitz@northwell.edu) |
| Michelle W. | Katzow |  | MD, MS | Department of Pediatrics | Northwell Health, Cohen Children's Medical Center, and the Zucker School of Medicine at Hofstra / Northwell | New Hyde Park, New York, USA | ECHO Cohort Study Site Co-Investigator | UG3OD035532 (Annemarie Stroustrup) | [mkatzow@northwell.edu](mailto:mkatzow@northwell.edu) |
| Zenobia | Brown |  | MD, MPH | Department of Science Education | Northwell Health and the Zucker School of Medicine at Hofstra / Northwell | New Hyde Park, New York, USA | ECHO Cohort Study Site Co-Investigator | UG3OD035532 (Annemarie Stroustrup) | [zbrown2@northwell.edu](mailto:zbrown2@northwell.edu) |
| Codruta | Chiuzan |  | PhD | Institute of Health System Science | Northwell Health, Feinstein Institutes for Medical Research | Manhasset, New York, USA | ECHO Cohort Study Site Co-Investigator | UG3OD035532 (Annemarie Stroustrup) | [cchiuzan@northwell.edu](mailto:cchiuzan@northwell.edu) |
| Timothy | Rafael |  | MD | Department of Obstetrics and Gynecology | Northwell Health and the Zucker School of Medicine at Hofstra / Northwell | New Hyde Park, New York, USA | ECHO Cohort Study Site Co-Investigator | UG3OD035532 (Annemarie Stroustrup) | [trafael@northwell.edu](mailto:trafael@northwell.edu) |
| Dawnette | Lewis |  | MD, MPH | Department of Obstetrics and Gynecology | Northwell Health and the Zucker School of Medicine at Hofstra / Northwell | New Hyde Park, New York, USA | ECHO Cohort Study Site Co-Investigator | UG3OD035532 (Annemarie Stroustrup) | [dlewis@northwell.edu](mailto:dlewis@northwell.edu) |
| Natalie | Meirowitz |  | MD | Department of Obstetrics and Gynecology | Northwell Health and the Zucker School of Medicine at Hofstra / Northwell | New Hyde Park, New York, USA | ECHO Cohort Study Site Co-Investigator | UG3OD035532 (Annemarie Stroustrup) | [nmeirowi@northwell.edu](mailto:nmeirowi@northwell.edu) |
| Brenda | Poindexter |  | MD | Department of Pediatrics | Children's Healthcare of Atlanta Emory University | Atlanta, Georgia, USA | ECHO Cohort Study Site Co-Investigator | UH3OD023320 (Judy Aschner) | [breda.pointdexter@emory.edu](mailto:breda.pointdexter@emory.edu) |
| Tebeb | Gebretsadik |  | MPH | Department of Biostatistics | Vanderbilt University Medical Center | Nashville, Tennessee, USA | ECHO Cohort Study Site Principal Investigator | UG3OD035516 and UG3OD035517 (Tina Hartert) | tebeb.gebretsadik@vumc.org |
| Sarah | Osmundson |  | MD, MSC | Department of Obstetrics and Gynecology | Vanderbilt University Medical Center | Nashville, Tennessee, USA | ECHO Cohort Study Site Principal Investigator | UG3OD035517 (Tina Hartert) | sarah.osmundson@vumc.org |
| Jennifer K. | Straughen |  | PhD | Department of Public Health Sciences | Henry Ford Health | Detroit, Michigan, USA | ECHO Cohort Study Site Principal Investigator | UG3OD035518 (Jennifer Straughen) | [jstraug1@hfhs.org](mailto:jstraug1@hfhs.org) |
| Amy | Eapen |  | MD | Division of Allergy and Clinical Immunology | Henry Ford Health | Detroit, Michigan, USA | ECHO Cohort Study Site Principal Investigator | UG3OD035518 (Jennifer Straughen) | [aeapen1@hfhs.org](mailto:aeapen1@hfhs.org) |
| Andrea | Cassidy-Bushrow |  | PhD | Department of Public Health Sciences | Henry Ford Health | Detroit, Michigan, USA | ECHO Cohort Study Site Co-Investigator | UG3/UH3OD023282 (James Gern) | acassid1@hfhs.org |
| Ganesa | Wegienka |  | PhD | Department of Public Health Sciences | Henry Ford Health | Detroit, Michigan, USA | ECHO Cohort Study Site Co-Investigator | UG3/UH3OD023282 (James Gern) | gwegien1@hfhs.org |
| Alex | Sitarik |  | MPH | Department of Public Health Sciences | Henry Ford Health | Detroit, Michigan, USA | ECHO Cohort Study Site Biostatistician | UG3/UH3OD023282 (James Gern) | asitari1@hfhs.org |
| Kim | Woodcroft |  | PhD | Department of Public Health Sciences | Henry Ford Health | Detroit, Michigan, USA | ECHO Cohort Study Site Co-Investigator | UG3OD035518 (Jennifer Straughen), UG3/UH3OD023282 (James Gern) | kwoodcr1@hfhs.org |
| Audrey | Urquhart |  | MPH | Department of Public Health Sciences | Henry Ford Health | Detroit, Michigan, USA | ECHO Cohort Study Site Epidemiologist | UG3OD035518 (Jennifer Straughen), UG3/UH3OD023282 (James Gern) | [aurquha1@hfhs.org](mailto:aurquha1@hfhs.org) |
| Albert | Levin |  | PhD | Department of Public Health Sciences | Henry Ford Health | Detroit, Michigan, USA | ECHO Cohort Study Site Co-Investigator | UG3OD035518 (Jennifer Straughen) | [alevin1@hfhs.org](mailto:alevin1@hfhs.org) |
| Tisa | Johnson-Hooper |  | MD | Department of Pediatrics | Henry Ford Health | Detroit, Michigan, USA | ECHO Cohort Study Site Co-Investigator | UG3OD035518 (Jennifer Straughen) | tjohnso2@hfhs.org |
| Brent | Davidson |  | MD | Department of Women's Health | Henry Ford Health | Detroit, Michigan, USA | ECHO Cohort Study Site Co-Investigator | UG3/UH3OD023282 (James Gern) | bdavids1@hfhs.org |
| Tengfei | Ma |  | PhD | Department of Public Health Sciences | Henry Ford Health | Detroit, Michigan, USA | ECHO Cohort Study Site Co-Investigator | UG3OD035518 (Jennifer Straughen) | tengfei.ma@hfhs.org |
| Emily S. | Barrett |  | PhD | Department of Biostatistics and Epidemiology | Environmental and Occupational Health Sciences Institute, Rutgers University | Piscataway, New Jersey, USA | ECHO Cohort Study Site Principal Investigator | UG3OD035527 (Emily S Barrett) | [emily.barrett@eoshi.rutgers.edu](mailto:emily.barrett@eoshi.rutgers.edu) |
| Martin J. | Blaser |  | MD | Center for Advanced Biotechnology & Medicine | Rutgers University | Piscataway, New Jersey, USA | ECHO Cohort Study Site Principal Investigator | UG3OD035527 (Emily S Barrett) | [blaser@cabm.rutgers.edu](mailto:blaser@cabm.rutgers.edu) |
| Maria Gloria | Dominguez-Bello |  | PhD | Departments of Biochemistry and Microbiology & Anthropology | Rutgers University | New Brunswick, New Jersey, USA | ECHO Cohort Study Site Principal Investigator | UG3OD035527 (Emily S Barrett) | [mg.dominguez-bello@rutgers.edu](mailto:mg.dominguez-bello@rutgers.edu) |
| Daniel B. | Horton |  | MD | Department of Pediatrics | Robert Wood Johnson Medical School, Rutgers University | New Brunswick, New Jersey, USA | ECHO Cohort Study Site Principal Investigator | UG3OD035527 (Emily S Barrett) | [daniel.horton@rutgers.edu](mailto:daniel.horton@rutgers.edu) |
| Manuel | Jimenez |  | MD | Departments of Pediatrics, Family Medicine, and Community Health | Robert Wood Johnson Medical School, Rutgers University | New Brunswick, New Jersey, USA | ECHO Cohort Study Site Principal Investigator | UG3OD035527 (Emily S Barrett) | [jimenema@rwjms.rutgers.edu](mailto:jimenema@rwjms.rutgers.edu) |
| Todd | Rosen |  | MD | Department of Obstetrics, Gynecology, and Reproductive Sciences | Robert Wood Johnson Medical School, Rutgers University | New Brunswick, New Jersey, USA | ECHO Cohort Study Site Co-Investigator | UG3OD035527 (Emily S Barrett) | [rosentj@rwjms.rutgers.edu](mailto:rosentj@rwjms.rutgers.edu) |
| Kristy | Palomares |  | MD, PhD | Department of Obstetrics and Gynecology | Saint Peter's University Hospital | New Brunswick, New Jersey, USA | ECHO Cohort Study Site Co-Investigator | UG3OD035527 (Emily S Barrett) | [kpalomares@saintpetersuh.com](mailto:kpalomares@saintpetersuh.com) |
| Lyndsay A. | Avalos |  | PhD, MPH | Division of Research | Kaiser Permanente Northern California | Oakland, California, USA | ECHO Cohort Study Site Principal Investigator | UG3OD035540 (Monique Marie Hedderson) | [Lyndsay.A.Avalos@kp.org](mailto:Lyndsay.A.Avalos@kp.org) |
| Yeyi | Zhu |  | PhD, MS | Division of Research | Kaiser Permanente Northern California | Oakland, California, USA | ECHO Cohort Study Site Principal Investigator | UG3OD035540 (Monique Marie Hedderson) | [Yeyi.Zhu@kp.org](mailto:Yeyi.Zhu@kp.org) |
| Kelly J . | Hunt |  | PhD | Department of Public Health Sciences | Medical University of South Carolina | Charleston, South Carolina, USA | ECHO Cohort Study Site Principal Investigator | UG3OD035543 (Kelly J Hunt) | [huntke@musc.edu](mailto:huntke@musc.edu) |
| Roger B. | Newman |  | MD | Department of Obstetrics and Gynecology | Medical University of South Carolina | Charleston, South Carolina, USA | ECHO Cohort Study Site Principal Investigator | UG3OD035543 (Kelly J Hunt) | [newmanr@musc.edu](mailto:newmanr@musc.edu) |
| Michael S. | Bloom |  | PhD | Department of Global and Community Health | George Mason University | Fairfax, Virginia, USA | ECHO Cohort Study Site Principal Investigator | UG3OD035543 (Kelly J Hunt) | [mbloom22@gmu.edu](mailto:mbloom22@gmu.edu) |
| Mallory H. | Alkis |  | MD | Department of Obstetrics and Gynecology | Medical University of South Carolina | Charleston, South Carolina, USA | ECHO Cohort Study Site Co-Investigator | UG3OD035543 (Kelly J Hunt) | [hudsonm@musc.edu](mailto:hudsonm@musc.edu) |
| James R. | Roberts |  | MD, MPH | Department of Pediatrics | Medical University of South Carolina | Charleston, South Carolina, USA | ECHO Cohort Study Site Co-Investigator | UG3OD035543 (Kelly J Hunt) | [robertsj@musc.edu](mailto:robertsj@musc.edu) |
| Sunni L. | Mumford |  | PhD | Department of Biostatistics, Epidemiology and Informatics; Department of Obstetrics and Gynecology | University of Pennsylvania Perelman School of Medicine | Philadelphia, Pennsylvania, USA | ECHO Cohort Study Site Principal Investigator | UG3OD035537 (Sunni L Mumford) | [sunni.mumford@pennmedicine.upenn.edu](mailto:sunni.mumford@pennmedicine.upenn.edu) |
| Heather H. | Burris |  | MD, MPH | Division of Neonatology, Department of Pediatrics | Children's Hospital of Philadelphia; University of Pennsylvania Perelman School of Medicine | Philadelphia, Pennsylvania, USA | ECHO Cohort Study Site Principal Investigator | UG3OD035537 (Sunni L Mumford) | [BURRISH@chop.edu](mailto:BURRISH@chop.edu) |
| Sara B. | DeMauro |  | MD, MSCE | Division of Neonatology, Department of Pediatrics | Children's Hospital of Philadelphia; University of Pennsylvania Perelman School of Medicine | Philadelphia, Pennsylvania, USA | ECHO Cohort Study Site Principal Investigator | UG3OD035537 (Sunni L Mumford) | [DEMAURO@chop.edu](mailto:DEMAURO@chop.edu) |
| Lynn M. | Yee |  | MD, MPH | Division of Maternal-Fetal Medicine, Department of Obstetrics & Gynecology | Feinberg School of Medicine, Northwestern University | Chicago, Illinois, USA | ECHO Cohort Study Site Principal Investigator | UG3OD035546 (Judy Aschner) | [lynn.yee@northwestern.edu](mailto:lynn.yee@northwestern.edu) |
| Aaron | Hamvas |  | MD | Division of Neonatology, Department of Pediatrics | Ann & Robert H. Lurie Children's Hospital, Feinberg School of Medicine, Northwestern University | Chicago, Illinois, USA | ECHO Cohort Study Site Principal Investigator | UG3OD035546 (Judy Aschner) | [ahamvas@luriechildrens.org](mailto:ahamvas@luriechildrens.org) |
| Antonia F. | Olidipo |  | MD, MSCI | Division of Maternal-Fetal Medicine, Department of Obstetrics & Gynecology | Hackensack University Medical Center, Hackensack Meridian School of Medicine | Nutley, New Jersey, USA | ECHO Cohort Study Site Co-Investigator | UG3OD035546 (Judy Aschner) | [antonia.olidipo@hmhn.org](mailto:antonia.olidipo@hmhn.org) |
| Andrew S. | Haddad |  | MD | Division of Maternal-Fetal Medicine, Department of Obstetrics & Gynecology | Hackensack University Medical Center, Hackensack Meridian School of Medicine | Nutley, New Jersey, USA | ECHO Cohort Study Site Co-Investigator | UG3OD035546 (Judy Aschner) | [andrews.haddad@hmhn.org](mailto:andrews.haddad@hmhn.org) |
| Lisa R. | Eiland |  | MD | Division of Neonatology, Department of Pediatrics | Hackensack University Medical Center, Hackensack Meridian School of Medicine | Nutley, New Jersey, USA | ECHO Cohort Study Site Co-Investigator | UG3OD035546 (Judy Aschner) | [lisa.eiland@hmhn.org](mailto:lisa.eiland@hmhn.org) |
| Nicole T. | Spillane |  | MD | Division of Neonatology, Department of Pediatrics | Hackensack University Medical Center, Hackensack Meridian School of Medicine | Nutley, New Jersey, USA | ECHO Cohort Study Site Co-Investigator | UG3OD035546 (Judy Aschner) | [nicole.spillane@hmhn.org](mailto:nicole.spillane@hmhn.org) |
| Kirin N. | Suri |  | MD | Division of Developmental and Behavioral Pediatrics, Department of Pediatrics | Hackensack University Medical Center, Hackensack Meridian School of Medicine | Nutley, New Jersey, USA | ECHO Cohort Study Site Co-Investigator | UG3OD035546 (Judy Aschner) | [kirin.suri@hmhn.org](mailto:kirin.suri@hmhn.org) |
| Stephanie A. | Fisher |  | MD, MPH | Division of Maternal-Fetal Medicine, Department of Obstetrics & Gynecology | Feinberg School of Medicine, Northwestern University | Chicago, Illinois, USA | ECHO Cohort Study Site Co-Investigator | UG3OD035546 (Judy Aschner) | [stephanie.fisher@northwestern.edu](mailto:stephanie.fisher@northwestern.edu) |
| Jeffrey A. | Goldstein |  | MD, PhD | Department of Pathology | Feinberg School of Medicine, Northwestern University | Chicago, Illinois, USA | ECHO Cohort Study Site Co-Investigator | UG3OD035546 (Judy Aschner) | ja.goldstein@northwestern.edu |
| Leena B. | Mithal |  | MD | Division of Infectious Diseases, Department of Pediatrics | Ann & Robert H. Lurie Children's Hospital, Feinberg School of Medicine, Northwestern University | Chicago, Illinois, USA | ECHO Cohort Study Site Co-Investigator | UG3OD035546 (Judy Aschner) | [lmithal@luriechildrens.org](mailto:lmithal@luriechildrens.org) |
| Raye-Ann O. | DeRegnier |  | MD | Division of Neonatology, Department of Pediatrics | Ann & Robert H. Lurie Children's Hospital, Feinberg School of Medicine, Northwestern University | Chicago, Illinois, USA | ECHO Cohort Study Site Co-Investigator | UG3OD035546 (Judy Aschner) | [r-deregnier@northwestern.edu](mailto:r-deregnier@northwestern.edu) |
| Nathalie L. | Maitre |  | MD, PhD | Division of Neonatology, Department of Pediatrics | Emory University School of Medicine and Cerebral Palsy Foundation | Atlanta, Georgia, USA and New York, New York, USA | ECHO Cohort Study Site Co-Investigator | UG3OD035546 (Judy Aschner) | [nathalie.linda.maitre@emory.edu](mailto:nathalie.linda.maitre@emory.edu) |
| Ruby H.N. | Nguyen |  | PhD, MHS | Division of Epidemiology & Community Health | School of Public Health, University of Minnesota | Minneapolis, Minnesota, USA | ECHO award Principal Investigator | UG3OD035529 (Hong-Ngoc Nguyen) | [Nguyen@umn.edu](mailto:Nguyen@umn.edu) |
| Meghan M. | JaKa |  | PhD, MS | Division of Research & Evaluation | HealthPartners Institute | Minneapolis, Minnesota, USA | ECHO site Principal Investigator | UG3OD035529 (Hong-Ngoc Nguyen) | [meghan.m.jaka@healthpartners.com](mailto:meghan.m.jaka@healthpartners.com) |
| Abbey C. | Sidebottom |  | PhD, MPH | Care Delivery Research | Allina Health | Minneapolis, Minnesota, USA | ECHO site Principal Investigator | UG3OD035529 (Hong-Ngoc Nguyen) | [abbey.sidebottom@allina.com](mailto:abbey.sidebottom@allina.com) |
| Michael J. | Paidas |  | MD | Department of Obstetrics and Gynecology | University of Miami Miller School of Medicine | Miami, Florida, USA | ECHO site Principal Investigator | UG3OD035542 (Hudson Santos) | mxp1440@med.miami.edu |
| JoNell E. | Potter |  | APRN, PhD | Department of Obstetrics, Gynecology and Reproductive Sciences | University of Miami Miller School of Medicine | Miami, Florida, USA | ECHO Cohort Study Site Co-Investigator | UG3OD035542 (Hudson Santos) | jpotter2@med.miami.edu |
| Natale | Ruby |  | PhD, PsyD | Mailman Center for Child Development | University of Miami Miller School of Medicine | Miami, Florida, USA | ECHO Cohort Study Site Co-Investigator | UG3OD035542 (Hudson Santos) | rnatale@med.miami.edu |
| Lunthita | Duthely |  | EdD | Department of Obstetrics, Gynecology and Reproductive Sciences and Department of Public Health Sciences | University of Miami School of Medicine | Miami, Florida, USA | ECHO Cohort Study Site Co-Investigator | UG3OD035542 (Hudson Santos) | [LDuthely@med.miami.edu](mailto:LDuthely@med.miami.edu) |
| Arumugam | Jayakumar |  | PhD | Department of Obstetrics, Gynecology and Reproductive Sciences | University of Miami Miller School of Medicine | Miami, Florida, USA | ECHO Cohort Study Site Co-Investigator | UG3OD035542 (Hudson Santos) | ajayakumar@med.miami.edu |
| Karen | Young |  | MD | Department of Pediatrics | University of Miami Miller School of Medicine | Miami, Florida, USA | ECHO Cohort Study Site Co-Investigator | UG3OD035542 (Hudson Santos) | [kyoung3@miami.edu](mailto:rjschmidt@ucdavis.edu) |
| Isabel | Maldonado |  | MPH, BS | School of Nursing and Health Studies | University of Miami | Miami, Florida, USA | ECHO Cohort Study Site Program Director | UG3OD035542 (Hudson Santos) | [icm16@miami.edu](mailto:rjschmidt@ucdavis.edu) |
| Meghan | Miller |  | PhD | Psychiatry and Behavioral Sciences; MIND Institute | University of California Davis | Sacramento, California, USA | ECHO Cohort Study Site Co-Investigator | UG3OD035550 (Rebecca Schmidt) | [mrhmiller@ucdavis.edu](mailto:mrhmiller@ucdavis.edu) |
| Jonathan L. | Slaughter |  | MD, MPH | Center for Perinatal Research, Abigail Wexner Research Institute and Division of Neonatology, Nationwide Children's Hospital and Department of Pediatrics, College of Medicine and Division of Epidemiology, College of Public Health, The Ohio State University | Nationwide Children's Hospital and The Ohio State University | Columbus, Ohio, USA | ECHO Cohort Study Site Principal Investigator | UG3OD035536 (Jonathan Slaughter) | [jonathan.slaughter@nationwidechildrens.org](mailto:jonathan.slaughter@nationwidechildrens.org) |
| Sarah A. | Keim |  | PhD, MS, MA | Center for Biobehavioral Health, Abigail Wexner Research Institute, Nationwide Children's Hospital and Department of Pediatrics, College of Medicine and Division of Epidemiology, College of Public Health, The Ohio State University | Nationwide Children's Hospital and The Ohio State University | Columbus, Ohio, USA | ECHO Cohort Study Site Principal Investigator | UG3OD035536 (Jonathan Slaughter) | [Sarah.Keim@nationwidechildrens.org](mailto:Sarah.Keim@nationwidechildrens.org) |
| Courtney D. | Lynch |  | PhD, MPH | Division of Maternal-Fetal Medicine, Department of Obstetrics and Gynecology, College of Medicine and Division of Epidemiology, College of Public Health, The Ohio State University | The Ohio State University | Columbus, Ohio, USA | ECHO Cohort Study Site Principal Investigator | UG3OD035536 (Jonathan Slaughter) | [Courtney.Lynch@osumc.edu](mailto:Courtney.Lynch@osumc.edu) |
| Kartik K. | Venkatesh |  | MD, PhD | Division of Maternal-Fetal Medicine, Department of Obstetrics and Gynecology, College of Medicine and Division of Epidemiology, College of Public Health, The Ohio State University | The Ohio State University | Columbus, Ohio, USA | ECHO Cohort Study Site Principal Investigator | UG3OD035536 (Jonathan Slaughter) | [kartik.venkatesh@osumc.edu](mailto:kartik.venkatesh@osumc.edu) |
| Kristina W. | Whitworth |  | PhD | Center for Precision Environmental Health and Department of Medicine | Baylor College of Medicine | Houston, Texas, USA | ECHO Cohort Study Site Principal Investigator | UG3OD035544 (Kristina Whitworth) | [kristina.whitworth@bcm.edu](mailto:kristina.whitworth@bcm.edu) |
| Elaine | Symanski |  | PhD | Center for Precision Environmental Health and Department of Medicine | Baylor College of Medicine | Houston, Texas, USA | ECHO Cohort Study Site Principal Investigator | UG3OD035544 (Kristina Whitworth) | [elaine.symanski@bcm.edu](mailto:elaine.symanski@bcm.edu) |
| Thomas F. | Northrup |  | PhD | Department of Family and Community Medicine | University of Texas Health Science Center at Houston (UTHealth Houston) McGovern Medical School | Houston, Texas, USA | ECHO Cohort Study Site Principal Investigator | UG3OD035544 (Kristina Whitworth) | [thomas.f.northrup@uth.tmc.edu](mailto:thomas.f.northrup@uth.tmc.edu) |
| Hector | Mendez-Figueroa |  | MD | Department of Obstetrics, Gynecology and Reproductive Sciences | University of Texas Health Science Center at Houston (UTHealth Houston) McGovern Medical School | Houston, Texas, USA | ECHO Cohort Study Site Co-Investigator | UG3OD035544 (Kristina Whitworth) | [hector.mendezfigueroa@uth.tmc.edu](mailto:hector.mendezfigueroa@uth.tmc.edu) |
| Ricardo A. | Mosquera |  | MD | Department of Pediatrics | University of Texas Health Science Center at Houston (UTHealth Houston) McGovern Medical School | Houston, Texas, USA | ECHO Cohort Study Site Co-Investigator | UG3OD035544 (Kristina Whitworth) | [ricardo.a.mosquera@uth.tmc.edu](mailto:ricardo.a.mosquera@uth.tmc.edu) |
| Margaret R. | Karagas |  | PhD | Department of Epidemiology | Geisel School of Medicine at Dartmouth | Hanover, New Hampshire, USA | ECHO Cohort Study Site Principal Investigator | UG3/UH3OD023275 (Margaret Karagas) | [margaret.karagas@dartmouth.edu](mailto:margaret.karagas@dartmouth.edu) |
| Juliette C. | Madan |  | MD, MS | Departments of Psychiatry, Pediatrics & Epidemiology | Geisel School of Medicine at Dartmouth, Dartmouth Hitchcock Medical Center | Hanover, New Hampshire, USA | ECHO Cohort Study Site Principal Investigator | UG3/UH3OD023275 (Margaret Karagas) | [juliette.madan@dartmouth.edu](mailto:juliette.madan@dartmouth.edu) |
| Debra M. | MacKenzie |  | PhD | Community Environmental Health Program, Department of Pharmaceutical Sciences | College of Pharmacy, University of New Mexico Health Sciences Center | Albuquerque, New Mexico, USA | ECHO Cohort Study Site Principal Investigator | UG3/UH3OD023344 (Debra MacKenzie) | [dmackenzie@salud.unm.edu](mailto:dmackenzie@salud.unm.edu) |
| Johnnye L. | Lewis |  | PhD | Community Environmental Health Program, Department of Pharmaceutical Sciences | College of Pharmacy, University of New Mexico Health Sciences Center | Albuquerque, New Mexico, USA | ECHO Cohort Study Site Principal Investigator | UG3/UH3OD023344 (Debra MacKenzie) | [jlewis@cybermesa.com; jlewis@salud.unm.edu](mailto:jlewis@cybermesa.com) |
| Brandon J. | Rennie |  | PhD | Center for Development and Disability | University of New Mexico | Albuquerque, New Mexico, USA | ECHO Cohort Study Site Co-Investigator | UG3/UH3OD023344 (Debra MacKenzie) | [Brennie@salud.unm.edu](mailto:Brennie@salud.unm.edu) |
| Bennett L. | Leventhal |  | MD | Community Environmental Health Program, Department of Pharmaceutical Sciences UNM | College of Pharmacy, University of New Mexico Health Sciences Center; University of Chicago | Albuquerque, New Mexico, USA; Chicago, Illinois, USA | ECHO Cohort Study Site Co-Investigator | UG3/UH3OD023344 (Debra MacKenzie) | [Bennett.leventhal@outlook.com](mailto:Bennett.leventhal@outlook.com) |
| Young Shin | Kim |  | MD, MS, MPH, PhD | Department of Psychiatry and Behavioral Sciences | University of California, San Francisco | San Francisco, California, USA | ECHO Cohort Study Site Co-Investigator | UG3/UH3OD023344 (Debra MacKenzie) | [Youngshin.Kim@ucsf.edu](mailto:Youngshin.Kim@ucsf.edu) |
| Somer | Bishop |  | PhD | Department of Psychiatry and Behavioral Sciences | University of California, San Francisco | San Francisco, California, USA | ECHO Cohort Study Site Co-Investigator | UG3/UH3OD023344 (Debra MacKenzie) | [Somer.Bishop@ucsf.com](mailto:Somer.Bishop@ucsf.com) |
| Sara S. | Nozadi |  | PhD | Community Environmental Health Program, Department of Pharmaceutical Sciences | College of Pharmacy, University of New Mexico Health Sciences Center | Albuquerque, New Mexico, USA | ECHO Cohort Study Site Co-Investigator | UG3/UH3OD023344 (Debra MacKenzie) | [snozadi@unm.edu](mailto:snozadi@unm.edu) |
| Li | Luo |  | PhD | Department of Internal Medicine | Comprehensive Cancer Center, University of New Mexico Health Sciences Center | Albuquerque, New Mexico, USA | ECHO Cohort Study Site Co-Investigator | UG3/UH3OD023344 (Debra MacKenzie) | [lluo@salud.unm.edu](mailto:lluo@salud.unm.edu) |
| Barry M. | Lester |  | PhD | Department of Pediatrics, Department of Psychiatry and Human Behavior | Warren Alpert Medical School of Brown University | Providence, Rhode Island, USA | ECHO Cohort Study Site Principal Investigator | UH3OD023347 (Barry Lester) | [barry_lester@brown.edu](mailto:barry_lester@brown.edu) |
| Carmen J. | Marsit |  | PhD | Department of Environmental Health | Rollins School of Public Health, Emory University | Atlanta, Georgia, USA | ECHO Cohort Study Site Principal Investigator | UH3OD023347 (Barry Lester) | carmen.j.marsit@emory.edu |
| Todd | Everson |  | PhD | Department of Environmental Health | Rollins School of Public Health, Emory University | Atlanta, Georgia, USA | ECHO Cohort Study Site Principal Investigator | UH3OD023347 (Barry Lester) | todd.m.everson@emory.edu |
| Cynthia M. | Loncar |  | PhD | Department of Psychiatry and Human Behavior | Warren Alpert Medical School of Brown University | Providence, Rhode Island, USA | ECHO Cohort Study Site Principal Investigator | UH3OD023347 (Barry Lester) | cloncar@kentri.org |
| Elisabeth C. | McGowan |  | MD | Department of Pediatrics | Warren Alpert Medical School of Brown University | Providence, Rhode Island, USA | ECHO Cohort Study Site Principal Investigator | UH3OD023347 (Barry Lester) | emcgowan@wihri.org |
| Stephen J. | Sheinkopf |  | PhD | Department of Pediatrics | Thompson Center for Autism & Neurodevelopment, University of Missouri | Columbia, Missouri, USA | ECHO Cohort Study Site Principal Investigator | UH3OD023347 (Barry Lester) | ssheinkopf@health.missouri.edu |
| Brian S. | Carter |  | MD | Department of Pediatrics | Children's Mercy-Kansas City | Kansas City, Missouri, USA | ECHO Cohort Study Site Principal Investigator | UH3OD023347 (Barry Lester) | bscarter@cmh.edu |
| Jennifer | Check |  | MD | Department of Pediatrics | Wake Forest School of Medicine | Winston, Salem North Carolina, USA | ECHO Cohort Study Site Principal Investigator | UH3OD023347 (Barry Lester) | jcheck@wakehealth.edu |
| Jennifer B. | Helderman |  | MD | Department of Pediatrics | Wake Forest School of Medicine | Winston, Salem North Carolina, USA | ECHO Cohort Study Site Principal Investigator | UH3OD023347 (Barry Lester) | jhelderm@wakehealth.edu |
| Charles R. | Neal |  | MD | Department of Pediatrics | University of Hawaii John A Burns School of Medicine | Honolulu, Hawaii, USA | ECHO Cohort Study Site Principal Investigator | UH3OD023347 (Barry Lester) | cneal@hphmg.org |
| Lynne M. | Smith |  | MD | Department of Pediatrics | UCLA Clinical and Translational Science Institute at The Lundquist Institute, Harbor-UCLA Medical Center | Los Angeles, California, USA | ECHO Cohort Study Site Principal Investigator | UH3OD023347 (Barry Lester) | smith@lundquist.org |
